# Engineering Enzyme Substrate Scope Complementarity for Promiscuous Cascade Synthesis of 1,2‐Amino Alcohols

**DOI:** 10.1002/anie.202212637

**Published:** 2022-10-18

**Authors:** Allwin D. McDonald, Samantha K. Bruffy, Aadhishre T. Kasat, Andrew R. Buller

**Affiliations:** ^1^ Department of Chemistry University of Wisconsin-Madison 1101 University Avenue Madison Wisconsin 53706 USA

**Keywords:** Amino Alcohols, Decarboxylase, Directed Evolution, Protein Engineering, Transaldolase

## Abstract

Biocatalytic cascades are uniquely powerful for the efficient, asymmetric synthesis of bioactive compounds. However, high substrate specificity can hinder the scope of biocatalytic cascades because the constituent enzymes may have non‐complementary activity. In this study, we implemented a substrate multiplexed screening (SUMS) based directed evolution approach to improve the substrate scope overlap between a transaldolase (ObiH) and a decarboxylase for the production of chiral 1,2‐amino alcohols. To generate a promiscuous cascade, we engineered a tryptophan decarboxylase to act efficiently on β‐OH amino acids while avoiding activity on l‐threonine, which is needed for ObiH activity. We leveraged this exquisite selectivity with matched substrate scope to produce a variety of enantiopure 1,2‐amino alcohols in a one‐pot cascade from aldehydes or styrene oxides. This demonstration shows how SUMS can be used to guide the development of promiscuous, C−C bond forming cascades.

## Graphical abstract

Biocatalytic cascades can be used for the efficient, asymmetric synthesis of bioactive compounds. A substrate multiplexed screening (SUMS) based directed evolution approach was implemented to improve the substrate scope overlap between a transaldolase and a decarboxylase. The matched substrate scope of the enzymes was leveraged to produce a variety of enantiomerically pure 1,2‐amino alcohols in a one‐pot cascade from aldehydes or styrene oxides.

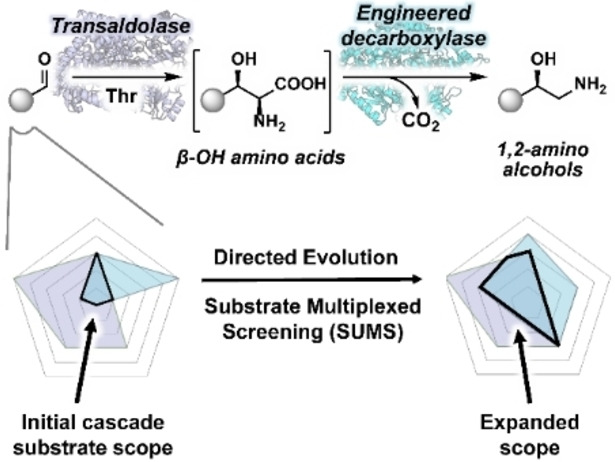

## Introduction

**Figure 1:**
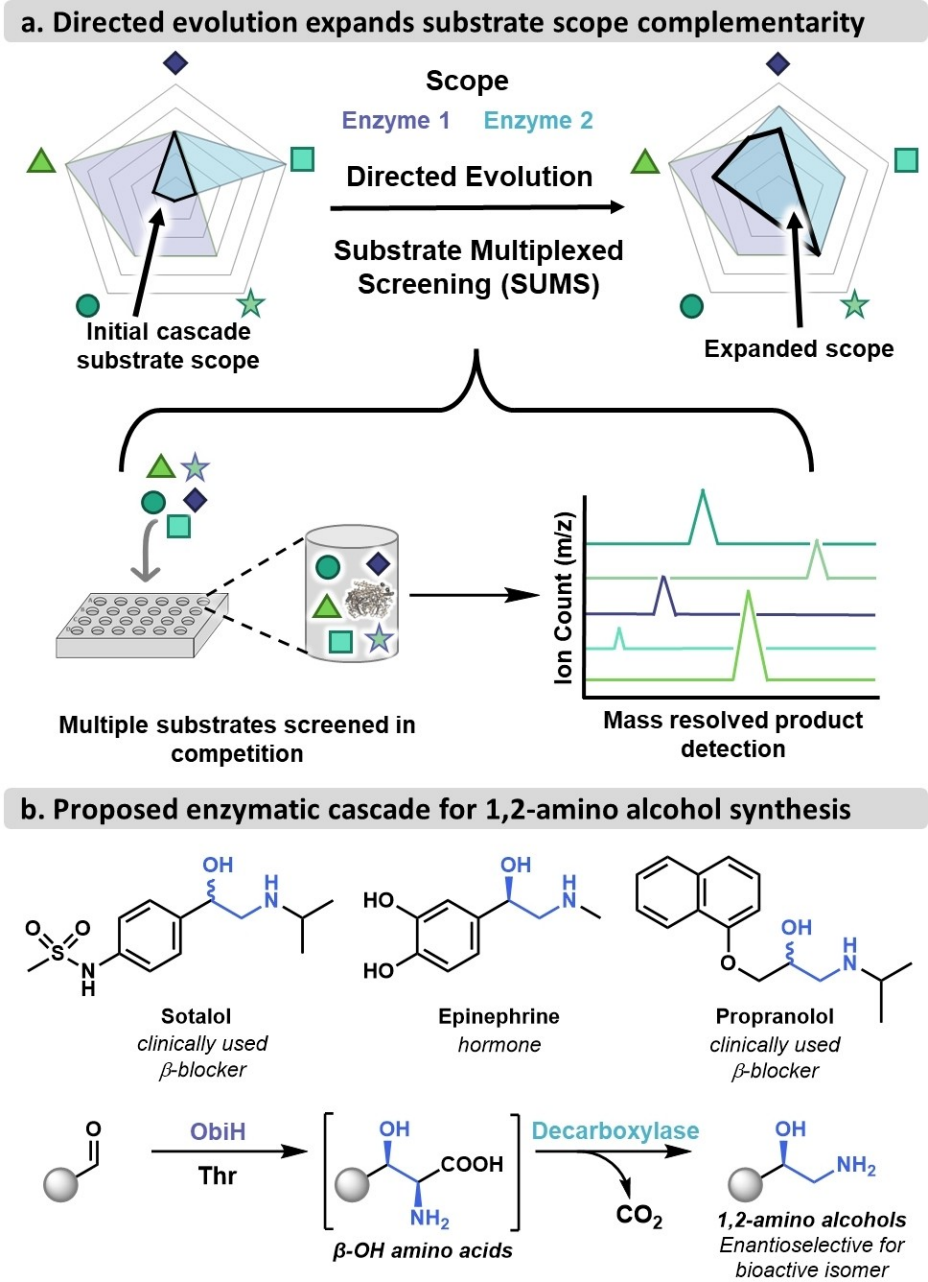
Substrate multiplexed screening (SUMS) has advantages for cascade engineering. a) Screening with multiple substrates can more efficiently expand substrate scopes. b) 1,2‐amino alcohols are a valuable bioactive motif that can be accessed via the proposed ObiH‐decarboxylase cascade.

One‐pot biocatalytic cascades are sought‐after for practical syntheses since they obviate the need for intermediate isolation and reduce waste.^[1]^ Such multi‐catalyst systems can also improve yield by coupling thermodynamically unfavorable reactions to favorable ones and limiting kinetic competition from unfavorable reaction pathways.^[2]^ Due to the high chemoselectivity of enzymes, side reactivity is often low, leading to effective and high‐yielding enzymatic cascades. For these reasons, biocatalytic cascades employing enzymes such as lipases, dehydrogenases, or transaminases have been used successfully for a wide variety of syntheses,[^2^, ^3^] including diverse process scale reactions.[^4^, ^5^, ^6^] However, many of these enzymatic cascades are conspicuously devoid of biocatalytic C−C bond forming steps.[^5^, ^7^, ^8^, ^9^, ^10^, ^11^] Although C−C bond forming reactions are especially valuable targets for biocatalytic cascades, enzymatic cascades to form C−C bonds often suffer from poor substrate scopes.[^12^, ^13^, ^14^] With the notable exception of cofactor regeneration, biocatalytic cascades are seldom deployed as “generalist” catalytic modalities, where a single cascade is capable of making diverse products. Mis‐matched substrate scopes hamper cascade utility, and one highly specific enzyme can be a cascade bottleneck (Figure 1a).[^15^, ^16^] The time and significant resources required to identify enzymes that operate on new substrates means that, often, bespoke enzymes are generated for each target cascade. This limitation in scope hinders the wider adoption of enzymes in organic synthesis. Therefore, development of methods to better control the scope of biocatalysts, particularly those that tailor carbon backbones, would help overcome a major hurdle in biocatalysis.

Recently, we explored a protein engineering screening method, substrate multiplexed screening (SUMS), to monitor changes in substrate scope during protein engineering.^[17]^ The SUMS method involves screening for activity on multiple substrates in competition and measuring each product that is formed, which provides a direct readout on biocatalyst promiscuity (Figure 1a). In this implementation, SUMS relies on selecting substrates that react to give unique product *m*/*z* ratios, enabling simultaneous quantitation via mass spectrometry (MS). Thus, information on both activity on individual substrates and specificity can be derived from a single assay, reducing uncertainties from the guess‐and‐check workflow of engineering promiscuous enzymes. Given the sparsity of examples where SUMS has been used, it remains unclear how substrate promiscuity might shift during multi‐step evolutionary campaigns and whether truly customized substrate scopes could be engineered using this approach.

Due to the wide chemical diversity of clinically used 1,2‐amino alcohols, most notably in β‐adrenergic receptor agonists (β‐blockers, Figure 1b),[^18^, ^19^, ^20^] there is continued motivation to expand routes to this class of compounds.[^21^, ^22^] Further, their relative simplicity serves as a fertile testing ground for promiscuous biocatalytic synthesis. Sehl et al. described a cascade that produced norephedrine stereoisomers in high yields from benzaldehyde using a lyase and transaminase.^[14]^ Other C−C bond forming cascades producing 1,2‐amino alcohols, such as using aminocyclases or imine reductases, have also been successful, but were limited in substrate diversity.[^23^, ^24^, ^25^] Griengl and co‐workers developed an enzymatic cascade to access enantio‐enriched 1,2‐amino alcohols by coupling a promiscuous threonine aldolase with a tyrosine decarboxylase.[^26^, ^27^] However, the utility of the cascade was limited by the activity of the decarboxylase, allowing preparative‐scale access to only a handful of aryl 1,2‐amino alcohols.^[27]^

**Figure 2:**
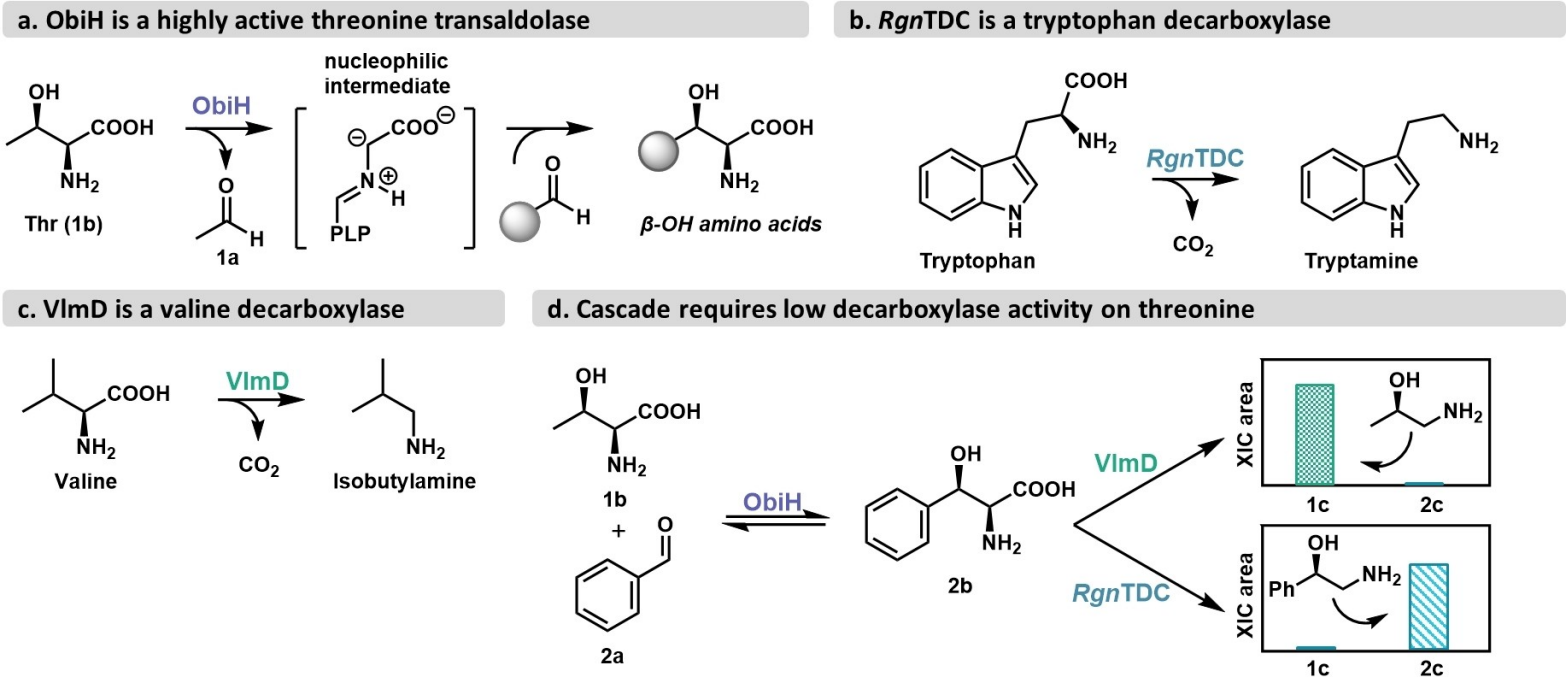
Enzymes under investigation for proposed ObiH‐decarboxylase cascade. a) General reaction of ObiH with aldehyde substrates. b) Native reaction of *Ruminococcus gnavus* tryptophan decarboxylase (*Rgn*TDC). c) Native reaction of *Kitasatospora setae* valine decarboxylase (VlmD). d) Product outcomes from either ObiH‐VlmD (top) or ObiH‐*Rgn*TDC (bottom) cascades from Thr (**1 b**) and benzaldehyde (**2 a**) to produce the corresponding decarboxylated products **1 c** and **2 c**. 10 mM benzaldehyde, 50 mM Thr, 400 μM pyridoxal‐5′‐phosphate (PLP), 50 mM potassium phosphate (KPi) buffer pH=8.0, and 10 μM VlmD/*Rgn*TDC (final volume=100 μL). Reactions were allowed to proceed for 16 h at 37 °C. XIC=extracted ion count; (M+1)/z=76 for **1 c** and (M+1)/z=138 for **2 c**. Full data shown in Figure S2.

We sought to develop a robust and highly generalizable C−C bond forming biocatalytic cascade to form 1,2‐amino alcohols from achiral aldehydes using an l‐threonine transaldolase (LTTA) and an amino acid decarboxylase. Recent work by our lab and others has characterized the activity and specificity of a promiscuous and highly stereoselective LTTA, ObiH.[^28^, ^29^, ^30^, ^31^] ObiH (also known as ObaG) catalyzes the formation of β‐OH amino acids from diverse aryl, α‐aryl, and aliphatic aldehydes (Figure 2a). However, no amino acid decarboxylase has been shown to have a scope that encompasses the wide array of amino acids produced via ObiH. Tyrosine decarboxylase was previously reported to have limited activity on aromatic β‐OH amino acids,[^26^, ^27^] but decarboxylation activity on β‐OH amino acids has not been explored for other classes of decarboxylases. We therefore set out to engineer an amino acid decarboxylase for cascade scope complementarity with ObiH.

We show here how SUMS can be deployed in a multi‐generational evolutionary campaign to efficiently monitor changes in substrate scope during evolution. Over the course of engineering, we identified gain‐of‐function mutations of tryptophan decarboxylase from *Ruminococcus gnavus* (*Rgn*TDC) for activity on aliphatic and benzylic β‐OH amino acids as well as increased activity on aryl β‐OH amino acids. We then showcase the utility of the engineered cascade for the preparative syntheses of diverse 1,2‐amino alcohols with good yields and high e.r.’s and use these products in the efficient total synthesis of β‐blockers.

## Results and Discussion

We first investigated two decarboxylases (DCs) as potential starting points for the ObiH‐DC cascade: *Rgn*TDC (Figure 2b) and the valine decarboxylase from *Kitasatospora setae* (VlmD, Figure 2c). We reasoned that assaying both a large aromatic amino acid decarboxylase and a short‐chain aliphatic amino acid decarboxylase would test distinct limits for active‐site steric tolerance for the β‐OH moiety. We assayed each enzyme for activity with a small set of β‐OH amino acids and found that both decarboxylases tolerate the β‐OH moiety. *Rgn*TDC had high activity on several β‐OH phenylalanine (Phe) analogs but had only trace activity on aliphatic β‐OH amino acids (Figure S1). VlmD showed the opposite activity trend, with high activity on smaller aliphatic β‐OH amino acids, including l‐threonine (Thr, **1 b**), and little to no activity on larger substrates (Figure S1).

To investigate the potential of these decarboxylases for cascade synthesis of 1,2‐amino alcohols, we assayed these enzymes along with ObiH to form (*R*)‐2‐amino‐1‐phenylethan‐1‐ol (**2 b**) from benzaldehyde (**2 a**) and Thr (Figure 2d). The ObiH‐VlmD cascade only produced trace amounts of the desired product (**2 c**). Instead, the decarboxylation product from Thr (**1 c**) was the major product, highlighting the specificity challenge of using these enzymes in concert (Figure 2d). Conversely, the ObiH‐*Rgn*TDC cascade produced **2 c** with negligible Thr decarboxylation. We therefore chose *Rgn*TDC as the decarboxylase of our cascade. This enzyme does not natively possess any activity on the aliphatic or benzylic β‐OH amino acid substrates that can be made by ObiH (Figure S1). Thus, the engineering goal for *Rgn*TDC was clear, and we set out to expand the scope of *Rgn*TDC so that it might compliment the scope of ObiH for cascade synthesis.

**Figure 3:**
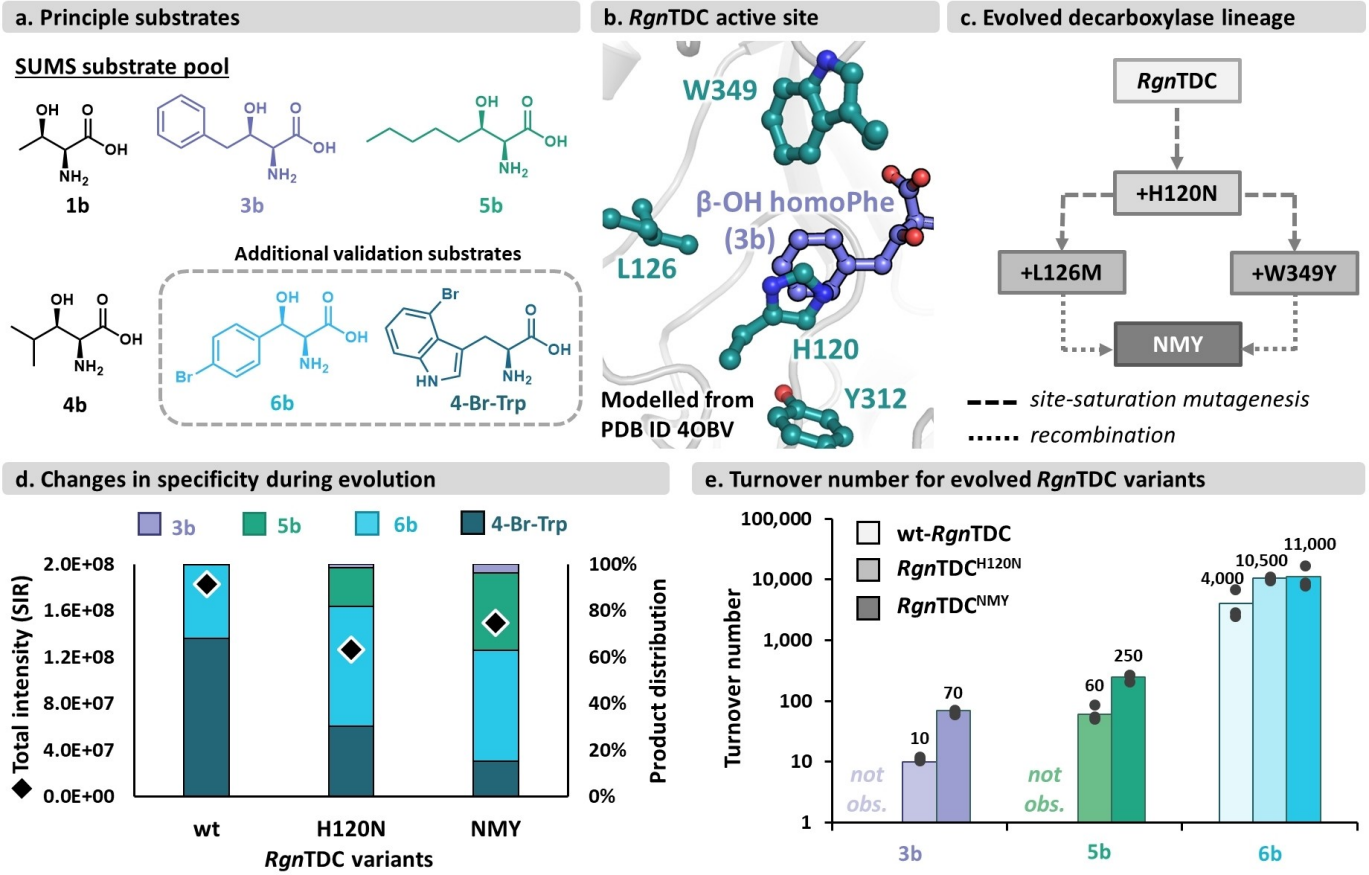
Engineering of *Rgn*TDC. a) Substrates used for engineering and validation (dashed box). b) Depiction of *Rgn*TDC active site with **3 b** modelled in (from PDB ID 4OBV). c) Evolutionary trajectory of *Rgn*TDC engineering. “NMY”=H120N, L126M, and W349Y *Rgn*TDC. d) Specificity for various amino acids over the course of *Rgn*TDC engineering from substrate multiplexed reactions. 2 mM **3 b**, 2 mM **5 b**, 0.2 mM **6 b**, and 2 mM 4‐bromotryptophan (4‐Br‐Trp), 400 μM PLP, 1 μM *Rgn*TDC variant, and 50 mM KPi buffer pH=7.5 (final volume=100 μL). Reactions were conducted in duplicate for 16 h at 37 °C. e) Turnover numbers for β‐OH amino acid for *Rgn*TDC variants. No product was observed for wt‐*Rgn*TDC for **3 b** or **5 b**. Reaction conditions are detailed in Materials and Methods.

Both substrate scope and sequence space must be considered for SUMS. Activity on the aromatic β‐OH amino acids was already high; engineering was therefore focused on identifying gain‐of‐function mutations for alkyl and benzylic substrates. If aromatic β‐OH substrates like **2 b** were included in the screen, they would act as competitive inhibitors and limit our ability to observe low levels of activity on less‐reactive substrates. We therefore opted not to include this class of compounds in the substrate pool for screening. If engineered variants lost activity on aryl β‐OH amino acids, we would still be able to use the wt‐*Rgn*TDC for preparative syntheses. We instead assayed for activity improvements on a substrate space of benzylic and short‐ and long‐chain aliphatic β‐OH substrates. We assayed for activity on: Thr **(1 b)**, β‐OH l‐homophenylalanine (**3 b**) β‐OH l‐leucine (**4 b**), and (2*S*,3*R*)‐2‐amino‐3‐hydroxyoctanoic acid (**5 b**) (Figure 3a), substrates on which the native enzyme does <1 turnover. Since the target cascade requires Thr, this amino acid was included to monitor whether any variant gains activity on this amino acid.

The first site in the protein that we targeted for site‐saturation mutagenesis (SSM) was His120. This residue is one of two that π‐stacks with the native Trp substrate in *Rgn*TDC and bioinformatic analysis showed that this residue is not conserved among decarboxylases. One mutation from the H120X library, H120N, was found to significantly boost activity on both **3 b** and **5 b** (Figure S3) but possessed no activity on **1 b** or **4 b** when screened in competition. This mutation could plausibly increase activity by introducing molecular recognition for the β‐OH group or, alternatively, simply aid in binding of sidechains that are less bulky than indole. To discriminate between these possibilities, we assayed purified *Rgn*TDC^H120N^ against a complementary set of amino acids that lack the β‐OH moiety: Trp, Phe, l‐leucine (Leu), and l‐homophenylalanine (homoPhe). We found significantly increased activity compared to wt‐*Rgn*TDC for both Leu and homoPhe (Figure S4), commensurate with boosts on their β‐OH counterparts. Additionally, we observed only a slight decrease in activity on Trp and Phe. These data suggest that the H120N mutation does not boost activity by specifically engaging the β‐OH moiety, but instead helps to accommodate the smaller side chains.

The H120N mutation gained activity on **3 b** and **5 b**, but the activity was still poor, prompting additional rounds of evolution using *Rgn*TDC^H120N^ as the parent. We targeted the active site with three additional SSM libraries (L126, Y312, W349, Figure 3b, Figure S5–S7). Most variants observed in the screens were deactivating, but the W349Y, W349F, and L126M mutations were further activating for **3 b** and **5 b** (Figure 3c, Figure S5, S7). As with wt‐*Rgn*TDC, no activity was detected on either **1 b** or **4 b**. Recombining these active‐site mutations, we found the triple mutant variant H120N/L126M/W349Y (hereafter called *Rgn*TDC^NMY^) further improved activity on substrates **3 b** and **5 b** (Figure S8).

We next isolated *Rgn*TDC^NMY^ and investigated changes in specificity, that is, activity on substrates in direct competition, in comparison to wt‐*Rgn*TDC and *Rgn*TDC^H120N^ (Figure 3d). For this comparison, we also included 4‐Br‐Trp to represent the native tryptophan substrate class. We observed that *Rgn*TDC^H120N^ and *Rgn*TDC^NMY^ both shifted specificity from 4‐Br‐Trp towards β‐OH amino acids, with *Rgn*TDC^NMY^ possessing the largest shift. To show that these changes in activity on complex mixtures translate to activity on single substrates, we measured the total turnover number (TTN) for each substrate in the DC lineage. These data showed activity on **3 b** and **5 b** was greatly improved (Figure 3e). Surprisingly, the H120N mutation also increased activity on β‐OH, *p*‐Br‐Phe (**6 b**), despite this substrate not being present in the screen. However, the additional L126M and W349Y mutations did not further increase activity with this substrate.

We again queried whether the boosts in decarboxylase activity were conferring specificity for the β‐OH group or were instead more reflective of a general mode of activation. We tested the *Rgn*TDC^NMY^ variant against the same des‐hydroxy amino acids that were assayed previously (Figure S4). We observed that the *Rgn*TDC^NMY^ variant had a 1.9‐fold increase in activity for homoPhe, compared to the 7‐fold activity increase for β‐OH homoPhe (Figure 3e). Unlike the H120 N mutation, therefore, L126M/W349Y seems to increase activity by interacting, either directly or indirectly, with the β‐OH moiety. These data are evidence of evolution producing a better generalist, as the final variant continues to improve with the lowest‐activity substrates and maintains or even slightly improves activity with the previously preferred substrates.

Aromatic amino acid decarboxylases have not previously been engineered for activity on aliphatic or β‐OH amino acids. The H120N mutation was an unexpected find, as His at this site is not conserved among aromatic amino acid decarboxylases, but Asn is present at the corresponding position in VlmD and other aliphatic amino acid decarboxylases (such as methionine decarboxylase) (Figure S9). The exact role of Asn for this substrate specificity is currently unclear. We also observed that this residue plays a role in the relative specificity between Trp and Phe (Figure S4), although to a lesser extent. Additionally, we note that both *Rgn*TDC and VlmD natively tolerate the β‐OH functionality, albeit with reduced turnovers compared to the des‐hydroxy amino acid analogs. Previously, a tyrosine decarboxylase was also reported to possess good activity with β‐OH amino acids.[^26^, ^27^] These data suggest that the β‐OH moiety might be well‐tolerated throughout different decarboxylase classes.

The ultimate test of these engineered DCs is whether the newfound increases in activity can enable productive cascade catalysis with ObiH. To this aim, we measured the effects of buffer, pH, and cosolvent conditions on these enzymes (Figure S10). We observed that ObiH had maximum activity in Tris buffer, while *Rgn*TDC preferred potassium phosphate (KP_i_) buffer. Given the diversity of our intended substrate scope, we envisioned using Tris as a buffer system for substrates challenging for ObiH and using KP_i_ as the buffer for substrates challenging for *Rgn*TDC.

In this cascade, there is the potential that both stereoisomers at Cβ are formed. In control experiments with a diastereomeric mixture of **6 b**, we found *Rgn*TDC^NMY^ possessed little to no selectivity for the configuration of the β‐OH group. (Figure S11). These data are consistent with our observation that engineering did not produce catalysts with high specificity for the β‐OH group. Consequently, we hypothesized that this “generalist” decarboxylase will exert no stereocontrol over the cascade. Instead, the e.r.’s of the products would reflect the stereoselectivity of the aldol addition catalyzed by ObiH. Previously, it was shown that ObiH forms β‐OH amino acids with excellent initial selectivity (>20 : 1 d.r.), and that the d.r. erodes at higher conversion as the products re‐enter the catalytic cycle.^[30]^ By coupling the engineered DC with ObiH, nascent amino acids can be decarboxylated before significant scrambling of the β‐stereocenter.

**Figure 4:**
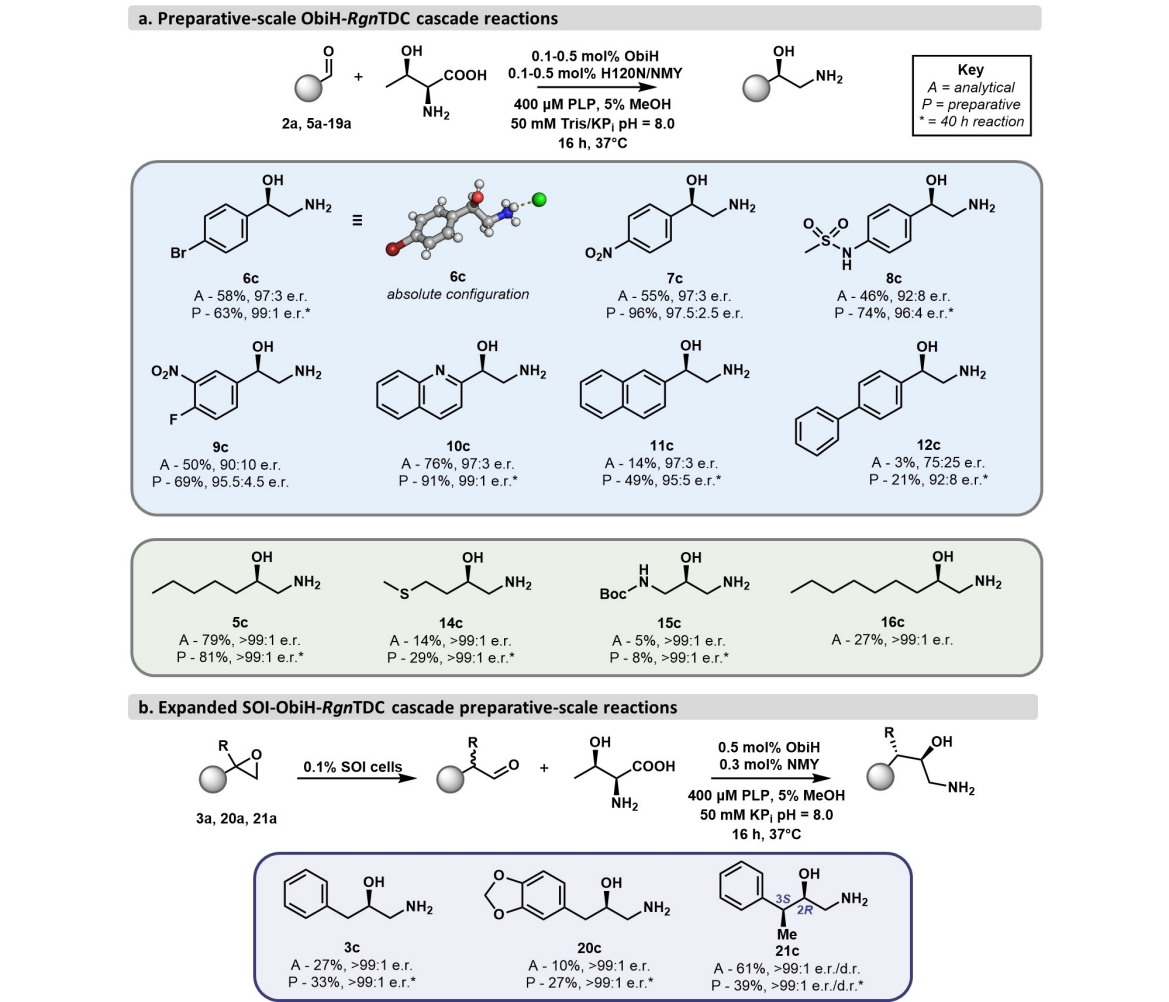
Substrate scope of the engineered ObiH—*Rgn*TDC cascade. a) Analytical (A) and preparative (P, isolated) yields of ObiH‐*Rgn*TDC cascade. Reactions run for 40 h are denoted by an asterisk (*). b) Analytical (A) and preparative (P, isolated) yields for expanded SOI‐ObiH‐*Rgn*TDC cascade. R=H, Me. Reactions used purified ObiH and NMY; SOI was introduced via whole cells overexpressing the enzyme.

With optimized cascade conditions in hand and an understanding of how the stereochemistry of the cascade is controlled, we investigated the scope of the two‐enzyme cascade. Initial reactions were conducted on analytical scale. The cascade produced a variety of aromatic 1,2‐amino alcohols in a range of yields (Figure 4a). We were initially unsure if *Rgn*TDC^NMY^ would be sufficiently active at the modest catalyst loadings used for these reactions (0.1–0.5 mol %) and maintain high product e.r.’s. However, most aromatic products were produced with high e.r.’s of >90 : 10. (**2 c**, **6 c**–**10 c**), indicating little re‐entry into the ObiH active site. The relatively insoluble napthyl (**11 a**) and biphenyl (**12 a**) substrates did not react efficiently in the cascade, and **12 c** was produced with a notably low e.r. of 75 : 25. The furanyl aldehyde (**13 a**) was not reactive with ObiH in this cascade, resulting in no observed product. Through engineering, *Rgn*TDC^NMY^ gained activity on numerous aliphatic β‐OH amino acids with diverse functionalities, such as **5 b** as well as thioether **14 b** and the Boc‐protected amine **15 b**. These analytical scale reactions suggested octanal (**16 a**) was accepted by the cascade as well, producing the long‐chain 1,2‐amino alcohol **16 c**. Short‐chain aliphatic β‐OH amino acids, bearing cyclopentyl, butyl, or isovaleryl groups (**17 b**–**19 b**), however, were not decarboxylated by *Rgn*TDC^NMY^ (Figure S12). All aliphatic products were observed with excellent e.r.’s. Since many of these reactions were observed with low overall conversion, we made several changes upon moving to the preparative scale in order to increase the yields. Reaction times were increased from 16 h to 40 h with most substrates. Additionally, flask shaking was implemented for poorly soluble aldehyde substrates to facilitate mass transfer during the reactions.

On preparative scale, aryl aldehydes remained the most active substrates for the two‐enzyme cascade and reacted to afford a series of epinephrine analogs (Figure 4a). *para‐*Substitutions such as ‐NO_2_, ‐Br, and ‐sulfonamido groups were well‐tolerated (**6 c**–**8 c**), and a *meta*‐substitution such as ‐NO_2_ was accepted as well (**9 c**). The *p*‐Br analog **6 c** was crystallized as the HCl salt and the X‐ray structure confirmed that the major product was the *R* isomer.^[32]^ Amino alcohols bearing heterocyclic and bicyclic groups such as 2‐quinolinyl, naphthyl, and biphenyl (**10 c**–**12 c**) were also produced from the cascade. Next, we applied the engineered cascade to synthesize aliphatic amino alcohols. ObiH was previously noted to synthesize aliphatic β‐OH amino acids with superb selectivity.^[30]^ Cascade reactions with hexanal proceeded smoothly to synthesize **5 c** in good yields and excellent stereochemical purity. Reactions with methional produced **14 c** in lower yields but with exceptional e.r. We additionally probed the reactivity of *N*‐Boc aminoacetaldehyde, which generated an asymmetrical 1,3‐diamino alcohol (**15 c**), albeit in poor yield. We highlight these preparative syntheses of aliphatic 1,2‐amino alcohols as examples of products previously unobtainable via the cascade with the native decarboxylase.

To access benzylic 1,2‐amino alcohols with this cascade, α‐arylaldehydes would be required as substrates. These are a notoriously reactive and unstable class of aldehyde. Therefore, we introduced a third enzyme, styrene oxide isomerase (SOI), to produce these compounds in situ from the corresponding epoxides. We recently reported the cascade synthesis of β‐OH amino acids using SOI and ObiH,^[31]^ which drew on the strong work of Li et al.[^33^, ^34^] With just 0.1 % w/v whole cells bearing SOI, the α‐arylaldehydes successfully entered the ObiH‐*Rgn*TDC^NMY^ cascade on analytical scale (Figure 4b). We found that *Rgn*TDC^NMY^ has relatively low activity on these challenging substrates; nevertheless, the three‐enzyme cascade furnished diverse benzylic 1,2‐amino alcohols with excellent e.r.’s, albeit with lower yields (**3 c**, **20 c**, **21 c**). The SOI‐ObiH cascade was previously characterized to produce **21 c** with a 65 : 35 d.r. with an *anti‐*relationship between the γ‐Me and β‐OH groups.^[31]^ Serendipitously, *Rgn*TDC^NMY^ was highly selective for the minor *syn* diastereomer, exclusively producing the corresponding *syn* 1,2‐amino alcohol (Figure S13). Although the activity with some of these substrates is low, further improvements could be obtained through additional rounds of directed evolution.

**Figure 5:**
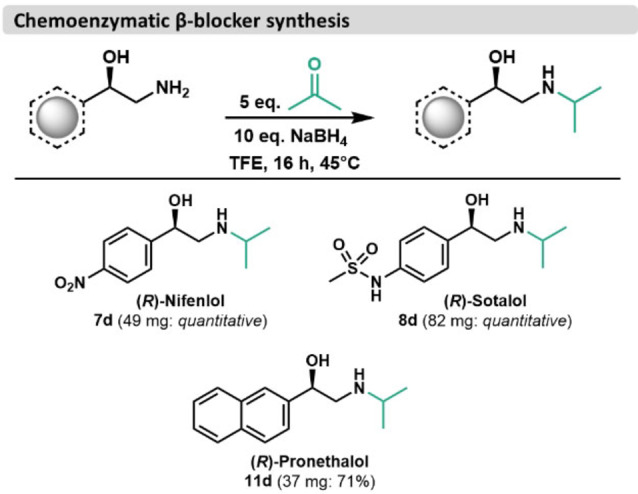
Preparative syntheses of β‐adenergenic receptor agonists (β‐blockers). Products were isolated via reverse‐phase chromatography on C18. Reaction conditions are detailed in Materials and Methods.

Enzymes often catalyze transformations that have no analog in the traditional synthetic repertoire and enable new retrosynthetic approaches to complex molecules.^[35]^ For example, 1,2‐amino alcohols have often been formed through ring‐opening of epoxides with an isopropyl amine nucleophile, relying on a pre‐assembled carbon skeleton.[^36^, ^37^, ^38^] To show how protecting group free biocatalysis can enable new disconnections, we completed the synthesis of some well‐known β‐blockers. Such molecules have been clinically used as racemic mixtures and the biologically active isomers have been identified as the *R*‐isomers.^[39]^ We considered performing the reductive amination reaction as a one‐pot reaction directly from the aldehyde starting materials, but opted against such an approach, considering the plethora of reactive amines present in the starting reaction mixture. Instead, we subjected isolated 1,2‐amino alcohols to reductive amination conditions with acetone following the procedure from Tajbakhsh et al. 2011 (Figure 5).^[40]^ This facile alkylation reaction afforded the bioactive isomers of the β‐blockers nifenlol (**7 d**) and pronethalol (**11 d**) as well as (*R*)‐sotalol (**8 d**) in good yields with retention of enantiopurity.

## Conclusion

Biocatalytic cascades can be a powerful approach to synthesis. While many cascades are efficient and selective, they can be hindered in their general utility by mis‐matched substrate scopes in the constituent enzymes. We report the engineering of a tryptophan decarboxylase for expanded substrate scope such that it complements the broad scope of an LTTA ObiH. The use of substrate multiplexed screening (SUMS) to guide enzyme promiscuity represents a milestone for in vitro directed evolution. We showed how the design of the substrate space with SUMS affords more flexibility than with single substrate screens. We found that SUMS was especially useful when searching for gain‐of‐function mutations, while simultaneously monitoring to ensure no mutation is selected that engenders activity on Thr, which must be preserved for use in the target cascade. Although we did not observe Thr decarboxylation in these screens, SUMS would have allowed us to recognize shifts in specificity towards Thr and avoid such variants. This simultaneous positive and negative selection demonstrates the customizability of MS‐based SUMS. The engineered decarboxylase combined with ObiH to form a versatile two‐enzyme cascade for the synthesis of chiral 1,2‐amino alcohols from achiral aldehydes and Thr. Based on these data, we hypothesize that SUMS will be a generalizable strategy for evolution, and its success here may motivate engineering of new promiscuous biocatalytic cascades for the synthesis of complex molecular scaffolds.

## Conflict of interest

The authors declare no conflict of interest.

1

## Supporting information

As a service to our authors and readers, this journal provides supporting information supplied by the authors. Such materials are peer reviewed and may be re‐organized for online delivery, but are not copy‐edited or typeset. Technical support issues arising from supporting information (other than missing files) should be addressed to the authors.

Supporting Information

## Data Availability

The data that support the findings of this study are available from the corresponding author upon reasonable request.
