# Supplementary material for: Engineering Enzyme Substrate Scope Complementarity for Promiscuous Cascade Synthesis of 1,2‐Amino Alcohols
Source: Angew Chem Int Ed Engl. 2022 Oct 18;61(46):e202212637. doi: 10.1002/anie.202212637 (PMC9643649; doi:10.1002/anie.202212637)
Supplement: Supplementary file 1 — Supporting Information [file ANIE-61-0-s001.pdf]

Angewandte Chemie
Eine Zeitschrift der Gesellschaft Deutscher Chemiker

Supporting Information

Engineering Enzyme Substrate Scope Complementarity for Promiscuous Cascade Synthesis of 1,2-Amino Alcohols

*A. D. McDonald, S. K. Bruffy, A. T. Kasat, A. R. Buller**

Table of Contents

Materials and Methods **S3**

- **Plasmid and protein information** **S3**
- **Cloning, expression, purification, and storage of ObiH/*Rgn*TDC/VlmD/SOI** **S11**
- **General Methods** **S16**
- **Optimization of ObiH-*Rgn*TDC Cascade** **S20**
- **Marfey's Analysis of Yields and e.r.'s/d.r.'s** **S21**

Synthetic Methods **S24**

Small Molecule Crystallography **S48**

Supplemental Figures **S50**

Supporting References **S93**

Materials and Methods

Chemicals and reagents were purchased from commercial suppliers (Sigma-Aldrich, VWR, Chem-Impex International, Alfa Aesar, Combi-blocks, Oakwood Products) at the highest quality available and used without further purification. β-OH amino acids were obtained from Prof. Tyler Doyon as previously prepared[1] or Tony Meza as previously described.[2] Genes were purchased as gBlocks from Integrated DNA Technologies (IDT). E. coli cells were electroporated with a Bio-Rad MicroPulser at 2500 V. New Brunswick I26R, 120 V/60 Hz shaker incubators (Eppendorf) were used for cell growth. Cell disruption via sonication was performed with a Sonic Dismembrator 550 (Fisher Scientific) sonicator. Optical density measurements were collected using an optical density reader (Amersham Biosciences). Ultra-high pressure liquid chromatography-mass spectrometry (UPLC-MS) data were collected on an Acquity UPLC (Waters) equipped with an Acquity PDA and QDA MS detector using either a BEH C18 column (Waters) or an Intrada Amino Acid column (Imtakt). Preparative column separations were performed on an Isolera One Flash Purification system (Biotage). NMR data were collected on a Bruker 500 MHz spectrometer equipped with a DCH cryoprobe. Signal positions were recorded in ppm with the abbreviations s, d, t, q, dd, and m, denoting singlet, doublet, triplet, quartet, doublet of doublets, and multiplet respectively. All coupling constants J are measured in Hz. High resolution mass spectrometry data were collected with a Q Extractive Plus Orbitrap (NIH 1S10OD020022-1) instrument with samples ionized by ESI.

Plasmid and protein information

Protein sequence of *N-His-ObiH* (*Pseudomonas fluorescens;* Uniprot accession code: A0A1X9LWZ7):

MGSSHHHHHHSSMSNVKQQTAQIVDWLSSTLGKDHQYREDSLSLTANENYPSALVRLTSGST
AGAFYHCSFPFEVPAGEWHFPEPGHMNAIADQVRDLGKTLIGAQAFDWRPNGGSTAEQALML

AACKPGEGFVHFAHRDGGHFALESLAQKMGIEIFHLPVNPTSLLIDVAKLDEMVRRNPHIRIVILD
QSFKLRWQPLAEIRSVLPDSCTLTYDMSHDGGLIMGGVFDSPLSCGADIVHGNTHKTIPGPQK
GYIGFKSAQHPLLVDTSLWVCPHLQSNCHAEQLPPMWVAFKEMELFGRDYAAQIVSNAKTLAR
HLHELGLDVTGESFGFTQTHQVHFAVGDLQKALDLCVNSLHAGGIRSTNIEIPGKPGVHGIRLG
VQAMTRRGMKEKDFEVVARFIADLYFKKTEPAKVAQQIKEFLQAFPLAPLAYSFDNYLDEELLA
AVYQGAQR

DNA sequence of *N-His-ObiH*:

ATGTCAAACGTGAAGCAGCAGACGGCGCAAATCGTAGATTGGTTGTCATCAACCTTGGGG
AAAGATCACCAATACCGCGAGGACTCCCTTTCACTTACCGCTAACGAGAACTACCCGTCGG
CATTAGTTCGTTTGACTTCAGGTTCGACCGCCGGCGCATTCTACCATTGTTCTTTCCCCTTC
GAGGTTCCTGCCGGGGAGTGGCACTTCCCGGAGCCCGGTCATATGAATGCAATTGCTGAC
CAGGTTCGTGATTTAGGTAAAACCTTGATTGGTGCCCAGGCATTCGACTGGCGTCCAAATG
GCGGATCAACCGCAGAACAGGCACTTATGCTGGCAGCATGTAAACCGGGAGAGGGGTTC
GTCCATTTTGCTCACCGCGACGGAGGCCATTTCGCTTTAGAATCTCTTGCGCAAAAGATGG
GCATCGAAATTTTCCACTTGCCTGTTAATCCGACCTCTCTGTTAATCGATGTCGCCAAATTG
GATGAAATGGTCCGCCGCAACCCGCATATTCGCATTGTCATTCTTGATCAGAGCTTTAAGC
TGCGCTGGCAACCCCTGGCCGAGATTCGTTCAGTTTTACCAGACTCATGCACGTTGACTTA
TGATATGAGTCATGATGGGGGATTAATTATGGGAGGTGTCTTCGATTCCCCCCTTAGCTGT
GGAGCTGACATCGTCCACGGCAATACTCACAAGACGATTCCTGGACCGCAAAAGGGGTAT
ATCGGTTTCAAGTCCGCGCAACATCCTTTATTAGTCGATACAAGTTTATGGGTATGCCCTCA
CCTTCAAAGTAACTGCCACGCCGAGCAGCTGCCGCCGATGTGGGTTGCCTTCAAGGAAAT
GGAATTATTTGGACGCGATTACGCTGCCCAAATTGTTTCAAACGCAAAAACCTTGGCTCGC
CATCTGCATGAACTGGGATTGGACGTGACCGGAGAATCCTTTGGATTCACACAGACACATC
AGGTCCATTTTGCTGTAGGAGATTTACAGAAAGCGCTTGATCTTTGTGTGAATTCATTACAT
GCTGGAGGTATCCGTTCGACCAATATTGAAATCCCAGGGAAACCAGGAGTACATGGCATTC

GCTTAGGCGTCCAAGCGATGACTCGTCGTGGCATGAAGGAGAAAGACTTTGAGGTGGTCG

CCCGTTTTATCGCCGATCTGTACTTTAAAAAAACGGAACCTGCCAAGGTCGCACAGCAAAT

TAAGGAATTTTTACAGGCGTTTCCGCTTGCACCTTTAGCCTACTCATTTGATAACTATCTTG

ATGAAGAATTATTGGCAGCCGTTTACCAGGGTGCGCAGCGCTGA

Protein sequence of *C-His-RgnTDC* (*Ruminococcus gnavus;* Uniprot accession code: A7B1V0):

MSQVIKKKRNTFMIGTEYILNSTQLEEAIKSFVHDFCAEKHEIHDQPVVVEAKEHQEDKIKQIKIP

EKGRPVNEVVSEMMNEVYRYRGDANHPRFFSFVPGPASSVSWLGDIMTSAYNIHAGGSKLAP

MVNCIEQEVLKWLAKQVGFTENPGGVFVSGGSMANITALTAARDNKLTDINLHLGTAYISDQTH

SSVAKGLRIIGITDSRIRRIPTNSHFQMDTTKLEEAIETDKKSGYIPFVVIGTAGTTNTGSIDPLTEI

SALCKKHDMWFHIDGAYGASVLLSPKYKSLLTGTGLADSISWDAHKWLFQTYGCAMVLVKDIR

NLFHSFHVNPEYLKDLENDIDNVNTWDIGMELTRPARGLKLWLTLQVLGSDLIGSAIEHGFQLA

VWAEEALNPKKDWEIVSPAQMAMINFRYAPKDLTKEEQDILNEKISHRILESGYAAIFTTVLNGK

TVLRICAIHPEATQEDMQHTIDLLDQYGREIYTEMKKALEHHHHHH

DNA sequence of *C-His-RgnTDC*:

ATGTCGCAGGTCATTAAGAAAAAACGCAATACGTTTATGATTGGAACGGAGTACATCCTTAA

TTCGACACAGTTAGAGGAGGCAATTAAGTCTTTCGTGCACGATTTTTGTGCGGAAAAACAT

GAGATCCATGATCAGCCCGTCGTTGTTGAAGCCAAGGAGCACCAGGAAGATAAAATTAAG

CAGATCAAGATCCCTGAAAAAGGACGCCCAGTAAATGAGGTCGTGAGTGAGATGATGAAT

GAAGTTTACCGCTATCGCGGAGATGCGAACCACCCCCGTTTCTTCTCCTTCGTTCCGGGTC

CAGCTTCGAGCGTCTCCTGGCTTGGAGACATCATGACGAGTGCATATAATATCCATGCCGG

AGGCAGTAAATTGGCTCCCATGGTAAACTGTATTGAGCAAGAAGTGCTGAAGTGGTTGGCA

AAGCAAGTGGGATTTACTGAAAATCCCGGCGGGGTGTTCGTCTCAGGTGGCTCGATGGCG

AACATCACGGCGTTAACAGCAGCCCGTGACAATAAACTTACTGACATTAATTTGCATTTAGG

AACGGCGTATATCAGCGACCAAACACACTCCAGTGTAGCCAAGGGGTTACGTATTATTGGC
ATCACCGACAGCCGTATTCGCCGTATTCCCACTAATTCGCACTTCCAAATGGATACGACCA
AGTTGGAGGAGGCCATTGAAACCGATAAAAAGAGTGGCTATATCCCGTTTGTAGTGATCGG
AACCGCTGGCACGACTAATACAGGATCCATTGACCCATTAACGGAAATTTCTGCATTATGTA
AAAAGCACGATATGTGGTTCCACATCGACGGTGCGTATGGTGCCTCCGTATTGCTTAGTCC
AAAATATAAGTCCCTTTTGACAGGAACAGGATTAGCAGATAGTATTTCTTGGGATGCTCACA
AATGGTTATTCCAGACGTATGGGTGCGCCATGGTATTGGTGAAGGACATCCGCAACCTGTT
CCATTCCTTTCACGTTAACCCCGAATATCTGAAAGACCTTGAGAATGACATTGATAATGTCA
ATACGTGGGATATTGGGATGGAGTTAACACGTCCGGCACGTGGGCTTAAACTTTGGCTGA
CCTTGCAGGTTCTTGGGTCCGACCTTATTGGGTCTGCAATTGAGCACGGTTTCCAATTAGC
GGTATGGGCGGAAGAAGCGCTGAATCCCAAAAAAGATTGGGAAATTGTTAGCCCTGCCCA
GATGGCGATGATTAATTTTCGCTACGCGCCTAAGGATTTAACCAAAGAGGAGCAGGACATC
CTTAATGAAAAGATTTCGCATCGCATCTTGGAATCAGGCTATGCCGCTATTTTTACTACTGT
GCTGAATGGTAAGACAGTGTTACGCATTTGCGCGATTCACCCTGAGGCTACTCAAGAGGAT
ATGCAGCACACCATTGATCTGTTGGACCAATACGGTCGCGAGATCTATACTGAAATGAAAA
AGGCTCTCGAGCACCATCACCATCACCATTGA

DNA sequence of *C-His-RgnTDC*^{H120N}:

ATGTCGCAGGTCATTAAGAAAAAACGCAATACGTTTATGATTGGAACGGAGTACATCCTTAA
TTCGACACAGTTAGAGGAGGCAATTAAGTCTTTCGTGCACGATTTTTGTGCGGAAAAACAT
GAGATCCATGATCAGCCCGTCGTTGTTGAAGCCAAGGAGCACCAGGAAGATAAAATTAAG
CAGATCAAGATCCCTGAAAAAGGACGCCCAGTAAATGAGGTCGTGAGTGAGATGATGAAT
GAAGTTTACCGCTATCGCGGAGATGCGAACCACCCCCGTTTCTTCTCCTTCGTTCCGGGTC
CAGCTTCGAGCGTCTCCTGGCTTGGAGACATCATGACGAGTGCATATAATATCAATGCCGG
AGGCAGTAAATTGGCTCCCATGGTAAACTGTATTGAGCAAGAAGTGCTGAAGTGGTTGGCA
AAGCAAGTGGGATTTACTGAAAATCCCGGCGGGGTGTTCGTCTCAGGTGGCTCGATGGCG

AACATCACGGCGTTAACAGCAGCCCGTGACAATAAACTTACTGACATTAATTTGCATTTAGG

AACGGCGTATATCAGCGACCAAACACACTCCAGTGTAGCCAAGGGGTTACGTATTATTGGC

ATCACCGACAGCCGTATTCGCCGTATTCCCACTAATTCGCACTTCCAAATGGATACGACCA

AGTTGGAGGAGGCCATTGAAACCGATAAAAAGAGTGGCTATATCCCGTTTGTAGTGATCGG

AACCGCTGGCACGACTAATACAGGATCCATTGACCCATTAACGGAAATTTCTGCATTATGTA

AAAAGCACGATATGTGGTTCCACATCGACGGTGCGTATGGTGCCTCCGTATTGCTTAGTCC

AAAATATAAGTCCCTTTTGACAGGAACAGGATTAGCAGATAGTATTTCTTGGGATGCTCACA

AATGGTTATTCCAGACGTATGGGTGCGCCATGGTATTGGTGAAGGACATCCGCAACCTGTT

CCATTCCTTTCACGTTAACCCCGAATATCTGAAAGACCTTGAGAATGACATTGATAATGTCA

ATACGTGGGATATTGGGATGGAGTTAACACGTCCGGCACGTGGGCTTAAACTTTGGCTGA

CCTTGCAGGTTCTTGGGTCCGACCTTATTGGGTCTGCAATTGAGCACGGTTTCCAATTAGC

GGTATGGGCGGAAGAAGCGCTGAATCCCAAAAAAGATTGGGAAATTGTTAGCCCTGCCCA

GATGGCGATGATTAATTTTCGCTACGCGCCTAAGGATTTAACCAAAGAGGAGCAGGACATC

CTTAATGAAAAGATTTCGCATCGCATCTTGGAATCAGGCTATGCCGCTATTTTTACTACTGT

GCTGAATGGTAAGACAGTGTTACGCATTTGCGCGATTCACCCTGAGGCTACTCAAGAGGAT

ATGCAGCACACCATTGATCTGTTGGACCAATACGGTCGCGAGATCTATACTGAAATGAAAA

AGGCTCTCGAGCACCATCACCATCACCATTGA

DNA sequence of *C-His-RgnTDC*$^{\mathbf{NMY}}$ (H120N, L126M, W349Y):

ATGTCGCAGGTCATTAAGAAAAAACGCAATACGTTTATGATTGGAACGGAGTACATCCTTAA

TTCGACACAGTTAGAGGAGGCAATTAAGTCTTTCGTGCACGATTTTTGTGCGGAAAAACAT

GAGATCCATGATCAGCCCGTCGTTGTTGAAGCCAAGGAGCACCAGGAAGATAAAATTAAG

CAGATCAAGATCCCTGAAAAAGGACGCCCAGTAAATGAGGTCGTGAGTGAGATGATGAAT

GAAGTTTACCGCTATCGCGGAGATGCGAACCACCCCCGTTTCTTCTCCTTCGTTCCGGGTC

CAGCTTCGAGCGTCTCCTGGCTTGGAGACATCATGACGAGTGCATATAATATCAATGCCGG

AGGCAGTAAAATGGCTCCCATGGTAAACTGTATTGAGCAAGAAGTGCTGAAGTGGTTGGCA

AAGCAAGTGGGATTTACTGAAAATCCCGGCGGGGTGTTCGTCTCAGGTGGCTCGATGGCG

AACATCACGGCGTTAACAGCAGCCCGTGACAATAAACTTACTGACATTAATTTGCATTTAGG

AACGGCGTATATCAGCGACCAAACACACTCCAGTGTAGCCAAGGGGTTACGTATTATTGGC

ATCACCGACAGCCGTATTCGCCGTATTCCCACTAATTCGCACTTCCAAATGGATACGACCA

AGTTGGAGGAGGCCATTGAAACCGATAAAAAGAGTGGCTATATCCCGTTTGTAGTGATCGG

AACCGCTGGCACGACTAATACAGGATCCATTGACCCATTAACGGAAATTTCTGCATTATGTA

AAAAGCACGATATGTGGTTCCACATCGACGGTGCGTATGGTGCCTCCGTATTGCTTAGTCC

AAAATATAAGTCCCTTTTGACAGGAACAGGATTAGCAGATAGTATTTCTTGGGATGCTCACA

AATGGTTATTCCAGACGTATGGGTGCGCCATGGTATTGGTGAAGGACATCCGCAACCTGTT

CCATTCCTTTCACGTTAACCCCGAATATCTGAAAGACCTTGAGAATGACATTGATAATGTCA

ATACGTATGATATTGGGATGGAGTTAACACGTCCGGCACGTGGGCTTAAACTTTGGCTGAC

CTTGCAGGTTCTTGGGTCCGACCTTATTGGGTCTGCAATTGAGCACGGTTTCCAATTAGCG

GTATGGGCGGAAGAAGCGCTGAATCCCAAAAAAGATTGGGAAATTGTTAGCCCTGCCCAG

ATGGCGATGATTAATTTTCGCTACGCGCCTAAGGATTTAACCAAAGAGGAGCAGGACATCC

TTAATGAAAAGATTTCGCATCGCATCTTGGAATCAGGCTATGCCGCTATTTTTACTACTGTG

CTGAATGGTAAGACAGTGTTACGCATTTGCGCGATTCACCCTGAGGCTACTCAAGAGGATA

TGCAGCACACCATTGATCTGTTGGACCAATACGGTCGCGAGATCTATACTGAAATGAAAAA

GGCTCTCGAGCACCATCACCATCACCATTGA

Protein sequence of *C-His-VlmD* (*Kitasatospora setae;* Uniprot accession code: E4N6B4):

MAALPTGIPFGPDDAAWSTGLDRLRTAGATRVSAPSADPRETYPQLPELPPGRFQLPARGLDA

TEYAQAEDLFRRYVEDHSSRSLGYQLHWSEDFARRLAPYLGLQLNNIGDPYQHGAFMPNSKV

LERAVLDYFASLWNAKWPHRAGDPESYWGYVLTMGASEGNIQALWNARECLSGKPLAGQPR

LPADTAHENPNARHPVVFFSRETHYSLTKAVNLLGLDTFHALGSSRYPDANPLGPGTEWPTEV

PCVGGVDGPGAIDVEKLSLLVRFFVRRGHPVFVNLNYGSTFKGAFDDVPEAARAVHEICAEYG

MAERTFPSDREGTGARPRPGYWIHVDAALGAAYVPYLRMARAAGLVESAPPPFDFRLPQVHS

LTVSAHKWMGAPWPCGVFMTRNGLRMPPPRSSEYIGGTDTTLSGSRNGFSALLMWDYLAHH

SYDDLARQAAECDRLARLAHERLLKLQSGLGVDLLVSRSPWSLAVRFRRPDEAILRRYSLAYET

LLVDGVERPYAHLYVVPHVTEGLIDALLRDLGQPGAFAGGAAALEHHHHHH

DNA sequence of *C-His-VlmD:*

ATGGCAGCTTTGCCCACAGGTATCCCATTTGGTCCCGATGACGCCGCCTGGTCCACTGGA

CTGGATCGCCTGCGCACTGCTGGCGCTACTCGCGTAAGCGCACCGTCAGCTGACCCGCG

CGAAACGTATCCGCAATTGCCCGAGTTGCCGCCGGGCCGCTTCCAACTGCCGGCTCGCG

GTCTGGATGCAACAGAATATGCTCAAGCAGAAGATCTTTTCCGCCGCTACGTGGAAGATCA

CTCTTCTCGTTCTTTGGGCTACCAGTTGCACTGGTCCGAGGACTTTGCCCGTCGCCTGGC

GCCCTATTTAGGATTGCAGCTGAATAATATTGGCGATCCTTATCAACATGGAGCGTTTATGC

CAAATAGCAAGGTATTGGAACGCGCTGTACTGGACTATTTTGCTAGTTTATGGAACGCCAA

ATGGCCACATCGTGCGGGAGATCCCGAAAGTTACTGGGGCTACGTGCTGACAATGGGCG

CGTCCGAAGGAAACATTCAGGCGTTGTGGAATGCACGTGAATGTCTTAGCGGAAAGCCCC

TGGCAGGTCAACCTCGTTTGCCCGCAGACACTGCACATGAGAACCCGAATGCTCGCCATC

CGGTTGTATTCTTTTCCCGTGAGACCCACTATTCACTTACAAAAGCCGTTAACTTATTGGGA

CTGGACACATTCCATGCACTTGGGTCTAGCCGCTATCCAGACGCAAATCCATTAGGTCCCG

GGACAGAGTGGCCGACTGAAGTACCATGTGTGGGAGGGGTCGATGGACCCGGCGCTATT

GACGTAGAGAAGCTTTCCCTGTTAGTTCGTTTCTTTGTACGTCGTGGCCACCCGGTTTTCG

TGAACCTGAATTACGGTAGTACCTTCAAAGGGGCCTTCGACGATGTGCCTGAAGCTGCGC

GTGCTGTTCATGAGATTTGTGCCGAGTACGGGATGGCAGAACGCACTTTCCCATCCGATC

GCGAAGGAACGGGAGCCCGCCCCCGCCCAGGGTACTGGATTCATGTGGACGCGGCTTTG

GGAGCCGCGTACGTGCCGTACTTACGTATGGCACGTGCGGCCGGGTTAGTTGAATCAGCA

CCACCCCCGTTTGACTTTCGCTTACCCCAAGTACATTCTCTGACAGTGTCCGCTCATAAGT

GGATGGGCGCCCCCTGGCCATGCGGTGTATTCATGACTCGCAATGGATTACGTATGCCGC

CCCCTCGCTCTTCCGAATACATCGGAGGAACGGACACCACACTGTCTGGGAGTCGCAATG

GATTTTCCGCGCTGTTAATGTGGGATTATCTTGCGCACCATAGTTATGACGATTTAGCACG

CCAAGCCGCAGAATGCGATCGTTTAGCGCGTTTGGCCCATGAACGTTTATTAAAGTTGCAG

AGTGGATTAGGGGTTGACCTGTTAGTAAGCCGTTCGCCATGGAGCCTTGCCGTACGTTTTC

GCCGCCCGGATGAAGCAATCTTGCGCCGTTATAGTCTTGCCTACGAGACCCTTCTTGTCGA

TGGTGTTGAACGCCCTTACGCTCATCTTTATGTTGTACCTCATGTTACTGAAGGACTTATCG

ATGCCCTTCTTCGCGATTTAGGACAGCCAGGTGCGTTTGCAGGGGGAGCGGCGGCT*CTC*

*GAG*CACCATCACCATCACCATTGA

Protein sequence of *C-His-SOI* (*Pseudomonas sp.* VLB120; Uniprot accession code: O50216):

MLHAFERKMAGHGILMIFCTLLFGVGLWMNLVGGFEIIPGYIIEFHVPGSPEGWARAHSGPALN

GMMVIAVAFVLPSLGFADKTARLLGSIIVLDGWSNVGFYLFSNFSPNRGLTFGPNQFGPGDIFS

FLALAPAYLFGVLAMGALAVIGYQALKSTRSRKAVPHAAAELEHHHHHH

DNA sequence of *C-His-SOI*:

ATGCTTCATGCGTTCGAGCGTAAGATGGCTGGACATGGAATTTTGATGATTTTTTGCACTTT

ACTGTTTGGGGTAGGTTTGTGGATGAACTTGGTGGGTGGGTTTGAAATCATTCCAGGTTAT

ATTATTGAGTTTCACGTGCCAGGCTCCCCTGAGGGATGGGCACGTGCCCACAGCGGACCC

GCCTTGAATGGCATGATGGTAATCGCAGTTGCATTCGTGTTACCCTCCTTGGGTTTTGCGG

ACAAAACGGCACGTTTGCTGGGCAGTATCATCGTTCTTGATGGGTGGTCAAACGTGGGCTT

TTACTTGTTTTCTAACTTTTCTCCCAATCGTGGTCTGACTTTTGGTCCGAATCAGTTCGGAC

CAGGTGACATCTTTAGTTTCTTGGCTTTGGCGCCTGCGTACCTGTTCGGGGTATTAGCGAT

GGGCGCACTTGCAGTTATTGGTTATCAAGCCCTGAAATCAACACGCTCTCGTAAAGCAGTC

CCTCATGCCGCAGCTGAGCTCGAGCACCATCACCATCACCATTGA

DNA Isolation and Storage: DNA was purified via gel electrophoresis and isolated using a DNA gel extraction kit (Zymo Research). All isolated DNA was stored at -20 ^{o}C.

Protein expression methods

Cloning, expression, purification, and storage of ObiH

A codon-optimized copy of the *Pseudomonas fluorescens* ObiH gene was purchased as a gBlock from Integrated DNA Technologies. This DNA fragment was inserted into a pET-28b(+) vector by the Gibson Assembly method.[3] BL21 (DE3) E. coli cells were subsequently transformed with the resulting cyclized DNA product via electroporation. After 45 min of recovery in Terrific Broth II (TB) media containing 0.4% glucose at 37 °C, cells were plated onto Luria-Burtani (LB) plates with 50 µg/mL kanamycin (KAN) and incubated overnight. Single colonies were used to inoculate 5 mL TB + 50 µg/mL KAN (TB-KAN), which were grown overnight at 37 °C, 200 rpm. Expression cultures, typically 1 L of TB-KAN, were inoculated from these starter cultures and shaken (180 rpm) at 37 °C. After 3.5 hours (OD_{600} = > 1.0), the expression cultures were chilled on ice. After 45 min on ice, ObiH expression was induced with 1 mM IPTG, and the cultures were expressed for 16 hours at 23 °C with shaking at 200 rpm. (We found protein yields to be generally invariable to exact cell OD_{600} at the time of induction) Cells were then harvested by centrifugation at 4,300×g at 4 °C for 15 min. Cell pellets were pink in color and were frozen and stored at -20 °C until purification.

To purify ObiH, cell pellets were thawed on ice and then resuspended in lysis buffer (50 mM potassium phosphate (KPi) buffer (pH = 8.0), 1 mg/mL Hen Egg White Lysozyme (GoldBio), 0.2 mg/mL DNaseI (GoldBio), 1 mM $MgCl_2$, and 200 µM pyridoxal 5′-phosphate (PLP)). A volume of 4 mL of lysis buffer per gram of wet cell pellet was used. After 45 min of shaking at 37 °C, cells were sonicated with a ½ in. tip for 10 min (1 s on; 1 s off). The resulting lysate was then spun down at 75,000×g to pellet cell debris. Upon successful lysis, the pellet was colorless whereas the supernatant was pink in color. Ni/NTA beads (GoldBio) were added to a gravity column and

the lysis supernatant ran over the bead bed for purification by Ni-affinity chromatography. The column was washed with 4 column volumes of 20 mM imidazole, 50 mM KPi buffer (pH = 8.0). Washing with higher concentrations of imidazole resulted in slow protein elution. ObiH was eluted with 250 mM imidazole, 50 mM KPi buffer (pH = 8.0). Elution of the desired protein product was monitored by the disappearance of its bright pink color (resulting from the release of ObiH) from the column. The protein product was dialyzed to < 50 µM imidazole in 50 mM Tris-HCl buffer (pH = 8.01) or 50 mM KPi buffer (pH = 8.08). Purified enzyme was flash frozen in pellet form by pipetting enzyme dropwise into a crystallization dish filled with liquid nitrogen. The enzyme was transferred to a plastic conical and stored at -80 °C until further use. Frozen pellets were thawed at room temperature and centrifuged before use. The concentration of protein was determined by Bradford assay after freeze-thawing using bovine serum albumin for a standard concentration curve. Generally, this procedure yielded > 300 mg per L culture. Protein purity was analyzed by sodium dodecyl sulfate-polyacrylamide (SDS-PAGE) gel electrophoresis using 12% polyacrylamide gels.

Cloning, expression, purification, and storage of *Rgn*TDC

A codon-optimized copy of the *Ruminococcus gnavus* tryptophan decarboxylase (*Rgn*TDC) gene was purchased as a gBlock from Integrated DNA Technologies. This DNA fragment was inserted into a pET-22b(+) vector by the Gibson Assembly method.[3] BL21 (DE3) E. coli cells were subsequently transformed with the resulting cyclized DNA product via electroporation. After 30 min of recovery in LB media at 37 °C, cells were plated onto LB plates with 100 µg/mL ampicillin (AMP) and incubated overnight. Single colonies were used to inoculate 5 mL TB + 100 µg/mL AMP (TB-AMP), which were grown overnight at 37 °C, 200 rpm. Expression cultures, typically 1 L of TB-AMP were inoculated from these starter cultures and shaken (180 rpm) at 37 °C. After 3.5 to 4 hours (OD_{600} > 1.5), the expression cultures were chilled on ice. After 45 min on ice, expression was induced with 1 mM IPTG, and the cultures were

supplemented with 0.5 mM indole. Cultures were expressed overnight at 23 °C with shaking at 180 rpm. Cells were then harvested by centrifugation at 4300xg at 4 °C for 15 min. Cell pellets were frozen and stored at -20 °C until purification.

To purify TDC, cell pellets were thawed on ice and then resuspended in lysis buffer (50 mM KPi buffer (pH = 8.0), 1 mg/mL Hen Egg White Lysozyme (GoldBio), 0.2 mg/mL DNaseI (GoldBio), 1 mM $MgCl_2$, and 200 μM pyridoxal 5′-phosphate (PLP)). A volume of 4 mL of lysis buffer per gram of wet cell pellet was used. After 45 min of shaking at 37 °C, cells were sonicated with a ½ in. tip for 10 min (1 s on; 1 s off). The resulting lysate was then spun down at 75,000×g to pellet cell debris. Ni/NTA beads (GoldBio) were added to a gravity column and the lysis supernatant ran over the bead bed for purification by Ni-affinity chromatography. The column was washed with 4 column volumes of 20 mM imidazole, 50 mM KPi buffer (pH = 8.0). Washing with higher concentrations of imidazole resulted in slow protein elution. TDC was eluted with 250 mM imidazole, 50 mM KPi buffer (pH = 8.0). Elution of the desired protein product was monitored by the disappearance of its bright yellow color (resulting from the release of TDC) from the column. The protein product was dialyzed to < 50 μM imidazole in 50 mM Tris-HCl buffer (pH = 8.01) or 50 mM KPi buffer (pH = 8.08). Purified enzyme was flash frozen in pellet form by pipetting enzyme dropwise into a crystallization dish filled with liquid nitrogen. The enzyme was transferred to a plastic conical and stored at -80 °C until further use. Frozen pellets were thawed at room temperature and centrifuged before use. The concentration of protein was determined by Bradford assay after freeze-thawing using bovine serum albumin for a standard concentration curve. Generally, this procedure yielded > 100 mg per L culture. Protein purity was analyzed by sodium dodecyl sulfate-polyacrylamide (SDS-PAGE) gel electrophoresis using 12% polyacrylamide gels.

Cloning, expression, purification, and storage of VlmD

A codon-optimized copy of the *Kitasatospora setae valine decarboxylase* (VlmD) gene was purchased as a gBlock from Integrated DNA Technologies. This DNA fragment was inserted into a pET-22b(+) vector by the Gibson Assembly method.[3] BL21 (DE3) E. coli cells were subsequently transformed with the resulting cyclized DNA product via electroporation. After 30 min of recovery in LB media at 37 °C, cells were plated onto LB plates with 100 µg/mL ampicillin (AMP) and incubated overnight. Single colonies were used to inoculate 5 mL TB + 100 µg/mL AMP (TB-AMP), which were grown overnight at 37 °C, 200 rpm. 0.5 L of TB-AMP was inoculated from these starter cultures and shaken (180 rpm) at 37 °C. After 3 hours (OD_{600} ~ 1.0), the expression culture was chilled on ice. After 1 h on ice, expression was induced with 1 mM IPTG, and the cultures were expressed for 16 hours at 23 °C with shaking at 180 rpm. Cells were then harvested by centrifugation at 4300xg at 4 °C for 15 min. Cell pellets were frozen and stored at -20 °C until purification.

To purify VlmD, cell pellets were thawed on ice and then resuspended in lysis buffer (50 mM KPi buffer (pH = 7.0), 1 mg/mL Hen Egg White Lysozyme (GoldBio), 0.2 mg/mL DNaseI (GoldBio), 1 mM $MgCl_2$, and 200 µM pyridoxal 5′-phosphate (PLP)). A volume of 4 mL of lysis buffer per gram of wet cell pellet was used along with 2.5% BugBuster. After 1 h of shaking at 37 °C, the lysate was spun down at 75,000×g to pellet cell debris. Ni/NTA beads (GoldBio) were added to a gravity column and the lysis supernatant ran over the bead bed for purification by Ni-affinity chromatography. The column was washed with 4 column volumes of 20 mM imidazole, 50 mM KPi buffer (pH = 7.0). VlmD was eluted with 250 mM imidazole, 50 mM KPi buffer (pH = 7.0). Elution of the desired protein product was monitored by the disappearance of its bright yellow color (resulting from the release of VlmD) from the column. The protein product was dialyzed to < 50 µM imidazole in 50 mM KPi buffer (pH = 7.0). Purified enzyme was flash frozen in pellet form by pipetting enzyme dropwise into a crystallization dish filled with liquid nitrogen.

The enzyme was transferred to a plastic conical and stored at -80 °C until further use. Frozen pellets were thawed at room temperature and centrifuged before use. The concentration of protein was determined by Bradford assay after freeze-thawing using bovine serum albumin for a standard concentration curve. This procedure yielded 70 mg per L culture.

Cloning, expression, purification, and storage of SOI

A codon-optimized copy of the *Pseudomonas sp.* VLB120 *StyC* (SOI) gene was purchased as a gBlock from Integrated DNA Technologies. This DNA fragment was inserted into a pET-22b(+) vector by the Gibson Assembly method[3]. BL21 (DE3) *E. coli* cells were subsequently transformed with the cyclized DNA product via electroporation. After 45 min of recovery in TB media at 37 °C, cells were plated onto LB plates with 100 μg/mL AMP and incubated overnight. Following initial cloning into the pET-22b(+) vector, the gene encoding SOI was transferred to a pBAD vector (p15A origin, KAN resistant).

Single colonies were used to inoculate 10 mL TB-KAN, which was grown overnight at 37 °C, 200 rpm. Expression cultures, typically 1 L of TB-KAN, were inoculated with starter cultures (1% inoculum) and shaken (200 rpm) at 37 °C. After ~3 hours (OD_{600} = ~0.6), the expression cultures were chilled on ice. After 30 min on ice, expression of the protein was induced with 0.2% w/v arabinose. The cultures were expressed for 16-24 hours at 20 °C with shaking at 200 rpm. Cells were then harvested by centrifugation at 4,300×*g* at 4 °C for 10 min. Cell pellets were extruded from a syringe and flash frozen in liquid nitrogen as small pellets to give flash frozen wet cells.

General Methods

wt-*Rgn*TDC and wt-VlmD activity assessment

Freeze-thawed wt-*Rgn*TDC and wt-VlmD was spin-filtered, and the supernatant was used in the following reaction: 10 mM amino acid, 500 μM PLP, 50 mM KPi buffer pH = 8.0, and 37.5 μM

enzyme (final volume = 100 µL). Reactions were allowed to proceed for 16 h at 37 °C. Reactions were quenched via addition of 300 µL acetonitrile (ACN) followed by 300 µL H_2O, which were then centrifuged to aggregate enzyme, and injected onto UPLC-MS for product detection. Product m/z areas were used to determine relative amounts of product formed (Fig S1).

Initial ObiH-*Rgn*TDC and ObiH-VlmD cascade reactions

Freeze-thawed wt-VlmD and wt-*Rgn*TDC was spin-filtered, and the supernatant was used in the following reaction: 10 mM benzaldehyde, 50 mM L-threonine (Thr), 400 µM pyridoxal 5'-phosphate (PLP), 50 mM KPi buffer pH = 8.0, and 10 µM VlmD/*Rgn*TDC (final volume = 100 µL). Reactions were allowed to proceed for 16 h at 37 °C. Reactions were quenched via addition of 300 µL ACN followed by 300 µL H_2O, which were then centrifuged to aggregate enzyme, and injected onto UPLC-MS for product detection. Product m/z areas were used to determine relative amounts of product formed (Fig S2).

Mutagenesis of wt-RgnTDC

Based on a previously reported structure of an *Rgn*TDC-inhibitor complex (PDB ID: 4OBV), we modelled 3-hydroxyhomophenylalanine (**3b**) bound substrate into the active site (Fig 3a). H120 was chosen for site-saturation mutagenesis (SSM). Primers were purchased from Integrated DNA Technologies (IDT). For this site, three primers encoding the degenerate codons NDT, VHG, and TGG at the codon of interest were mixed in a 12:9:1 ratio, respectively.[4] The gene library was amplified first as two separate fragments and then combined via polymerase chain assembly (PCA) to form full-length RgnTDC gene mutagenized at the site of interest.[3] The corresponding gene was then inserted into a pET-22b(+) vector as described above and then transformed into BL21 (DE3) E. coli cells and plated on LB + 100 µg/mL AMP agar plates.

Mutagenesis of *Rgn*TDC^{H120N} double/triple mutants

Starting from *Rgn*TDC^{H120N}, we next mutagenized further 1st- and 2nd-sphere active-site residues: L126, Y312, and W349 (Fig 3a). Primers were ordered and SSM libraries prepared following the above protocol.

To investigate triple mutants, primers encoding H120N and L126M were used to add these mutations onto W349Y- and W349F-containing *Rgn*TDC gene sequences via PCA. These two gene sequences were transformed and expressed as above. *Rgn*TDC^{NMY} = H120N, L126M, W349Y; *Rgn*TDC^{NMF} = H120N, L126M, W349F.

Screening of *Rgn*TDC libraries

Cell pellets were thawed and then resuspended in lysis buffer: 50 mM KPi buffer (pH = 8.0), 1 mg/mL Hen Egg White Lysozyme (GoldBio), 0.2 mg/mL DNaseI (GoldBio), 1 mM $MgCl_2$, and 300 µM PLP. A volume of 600 µL lysis buffer per well was used. After 45 min of shaking at 37 °C, the resulting lysate was then spun down at 4000 x*g* to pellet cell debris. Then, 140 µL of the resulting supernatant was added to 60 µL of a substrate mixture in a separate reaction plate. Final substrate concentrations are as follows: 2.5 mM Thr (**1b**), 5 mM **3b,** and 2.5 mM 3-hydroxyleucine (**4b**), and 2.5 mM 2-amino-3-hydroxyoctanoic acid (**5b**). Reactions were incubated at 37 °C for 16 h, and then 100 µL reaction solution was quenched via addition of 200 µL ACN and centrifuged at 4000 x*g* for 10 min. 200 µL of the quenched reaction mixture supernatant was filtered into a 96-well plate for UPLC-MS analysis. Data were collected on an Acquity UHPLC with an Acquity QDA MS detector (Waters) using a BEH C18 column (Waters). Product m/z ion counts were used to assess product formation from the reaction mixture (Fig S3, S5-S7).

Lysate verification of activated *Rgn*TDC variants

Cells from wells containing putatively activated *Rgn*TDC variants (H120N, H120N/L126M, H120N/W349Y, and H120N/L126M/W349Y) were streaked onto LB + AMP agar plates. Individual colonies were used to inoculate 5 mL TB + AMP in biological triplicate for each mutant and grown for 16 h at 37 °C. Variant strain cultures were used to inoculate 5 mL TB + AMP, which was expressed, induced, and collected as described above. Cell pellets were frozen at -20 °C prior to use. Cell pellets were thawed at room temperature and then resuspended in 1 mL lysis buffer (50 mM KPi buffer (pH = 8.0), 1 mg/mL Hen Egg White Lysozyme (GoldBio), 0.2 mg/mL DNaseI (GoldBio), 1 mM $MgCl_2$, and 300 μM PLP). Lysis was conducted at 37 °C for 1 h. Lysates were centrifuged at 20,000 x*g* for 15 min, and the supernatants were used for the following reactions: 90 μL lysate supernatant + 10 μL 100 mM β-OH amino acid (**3b**, **4b**, or **5b**). Reactions progressed at 37 °C for 4 h and quenched via addition of 180 μL ACN to 20 μL reaction solution. Quenched reactions were then diluted with 200 μL H_2O, centrifuged at 20,000 x*g* for 10 min to pellet enzyme, and then products analyzed via LC-MS using single ion retention channels for the expected products (Fig S8).

*Rgn*TDC des-hydroxy amino acid activity comparison

Freeze-thawed *Rgn*TDC variants were spin-filtered, and the supernatants used for the following reaction: 10 mM amino acid, 400 μM PLP, 10 μM *Rgn*TDC (for Leu/homoPhe) or 0.05 μM *Rgn*TDC (for Trp/Phe), and 50 mM KPi buffer pH = 8.0 (final volume = 100 μL). Reactions were allowed to proceed for 16 h at 37 °C. Reactions were quenched via addition of 300 μL ACN followed by 300 μL H_2O, which were then centrifuged to aggregate enzyme, and injected onto UPLC-MS for product detection. Product m/z areas were used to determine relative amounts of product formed (Leu/homoPhe) or UV-vis peak areas at 254 nm (Phe) or 280 nM (Trp) were used to determine relative conversions (Fig S4).

*Rgn*TDC diastereoselectivity for β-OH moiety

Freeze-thawed *Rgn*TDC variants were spin-filtered, and the supernatants used for the following reaction: 10 mM *p*-Br, β-OH Phe **(6b)** 80:20 threo:erythro d.r., 400 μM PLP, 10 μM *Rgn*TDC variant, and 50 mM KPi buffer pH = 8.0 (final volume = 100 μL). Reactions were allowed to proceed for 15 min at 37 °C. Reactions were quenched via addition of 300 μL ACN followed by 300 μL H_2O, which were then centrifuged to aggregate enzyme, and injected onto UPLC-MS for product detection. UV-vis peak areas at 254 were used to determine relative concentrations of remaining amino acid starting material to observe changes in d.r. over the course of the reaction (Fig S11).

*Rgn*TDC lineage amino acid specificity measurements

Freeze-thawed *Rgn*TDC variants were spin-filtered, and the supernatants used for the following reactions: 2 mM β-OH homoPhe (**3b**), 2 mM β-OH Hex (**5b**), 0.2 mM *p*-Br, β-OH Phe (**6b**), and 2 mM 4-bromotryptophan, 400 μM PLP, 1 μM *Rgn*TDC variant, and 50 mM KPi buffer pH = 7.5 (final volume = 100 μL). Reactions were conducted in duplicate for 16 h at 37 °C. Reactions were quenched using 500 μL ACN, centrifuged to pellet aggregated enzyme, and injected onto UPLC-MS for product detection. Product m/z's were used to quantify relative product abundances (Fig 3d).

For standard amino acid specificity determination, freeze-thawed RgnTDC variants were spin-filtered, and the supernatants used for the following reactions: 2 mM each substrate (His, Leu, Tyr, Phe, Trp), 400 μM PLP, 1 μM *Rgn*TDC variant, and 50 mM KPi buffer pH = 8.0 (final volume = 100 μL). Reactions were conducted in duplicate for 10 min at 37 °C. Reactions were quenched using 500 μL ACN, centrifuged to pellet aggregated enzyme, and injected onto UPLC-MS for product detection. Product m/z's were used to quantify relative product abundances (Fig S4).

Optimization of ObiH-TDC Cascade

Buffer conditions:

Freeze-thawed ObiH and *Rgn*TDC^{H120N} was spin-filtered, and the supernatants used for the following reaction: 25 mM 3,4-dichlorobenzaldehyde, 75 mM Thr, 400 µM PLP, 10 µM ObiH, 25 µM *Rgn*TDC^{H120N}, and 50 mM Tris/KPi buffer with 5% MeOH (final volume = 250 µL). Buffer pH's ranged from pH = 7.0 to pH = 8.5. Reactions were conducted for 16 h at 37 °C at 180 rpm. Reactions were quenched using 500 µL ACN, centrifuged to pellet aggregated enzyme, and injected onto UPLC-MS for product detection. Product m/z areas were used to quantify relative product abundances. Each reaction condition was conducted in duplicate.

Additionally, *Rgn*TDC activity was assayed independently under different buffer conditions. Freeze-thawed *Rgn*TDC^{NMY} was spin-filtered, and the supernatant used for the following reaction: 10 mM β-OH homoPhe (**3b**), 400 µM PLP, 10 µM *Rgn*TDC^{NMY}, and 50 mM Tris/KPi buffer (final volume = 100 µL). Buffer pH's ranged from pH = 7.0 to pH = 8.5. Reactions were conducted for 16 h at 37 °C at 180 rpm. Reactions were quenched using 300 µL ACN, followed by 300 µL H_2O, centrifuged to pellet aggregated enzyme, and injected onto UPLC-MS for product detection. Product m/z areas were used to quantify relative product abundances. Each reaction condition was conducted in duplicate (Fig S10)

Co-solvent conditions:

Freeze-thawed ObiH and *Rgn*TDC^{H120N} was spin-filtered, and the supernatants used for the following reaction: 25 mM 4-chlorobenzaldehyde, 80 mM Thr, 200 µM PLP, 50 mM Tris-HCl buffer pH = 8.0, 32 µM ObiH, 12 µM *Rgn*TDC^{H120N} (final volume = 250 µL), with differing v/v % cosolvents (0–20% MeOH/DMSO/ACN). Reactions were conducted for 16 h at 37 °C without agitation. Reactions were quenched using 500 µL ACN, centrifuged to pellet aggregated enzyme, and injected onto UPLC-MS for product detection (Fig S10).

Marfey's Analysis of Yields and e.r.'s/d.r.'s

General Procedure: All reactions were done in triplicate on analytical scale (250 µL). Stocks of Thr were made in either 50 mM Tris-HCl buffer pH = 8.0 or 50 mM KPi buffer pH = 8.0 and aldehydes were prepared in MeOH. Derivatized amino acid product quantitation was performed by PDA analysis, integrating the area under the product curve at 340 nm and correcting by comparing to the internal standard (arginine) peak area. To calculate product concentrations, a standard curve was generated by subjecting stock solutions of L-Phe in buffer using the identical procedure used to process and derivatize enzymatic reaction solutions, in duplicate. This curve was used to calculate the concentration of 1,2-amino alcohol product in solution.

In a microcentrifuge tube, 30 µL of quenched reaction mix (1 equiv., 1.0 mM final total amines from unreacted Thr, Tris, unreacted β-hydroxy-α-amino acid, and 1,2-amino alcohol product) was added to a solution of 120 µL of 0.25 mM arginine dissolved in 18.75 mM sodium bicarbonate ($NaHCO_{3)}$ (7.5 equiv., 0.1 mM final concentration arginine and 7.5 mM final concentration $NaHCO_3$) followed by the addition of 150 µL 5 mM (*S*)-1-fluoro-2-4-dinitrophenyl-5-L-valine amide (L-FDVA) dissolved in ACN (2.5 equiv., 2.5 mM final concentration) to bring the total reaction volume to 300 µL. Each reaction was placed in a dark 37 °C incubator for 12 h, then quenched with 300 µL of 1:1 ACN:60 mM HCl (15 mM post-quench) before analyzing by UPLC-MS.

Determination of turnover numbers for TDC variants via Marfey's derivation:

Freeze-thawed *Rgn*TDC variants were spin-filtered, and the supernatants were used in the following reaction: 10 mM amino acid (**3b**, **5b,** or **6b**) 500 µM PLP, 50 µM *Rgn*TDC variant (for **3b** reaction), 20 µM *Rgn*TDC variant (for **5b** reaction), or 0.3 µM *Rgn*TDC variant (for **6b** reaction), and 50 mM KPi buffer pH = 8 (final volume = 100 µL). Reactions were allowed to proceed in a 37 °C incubator for 16 h prior to quenching with 100 µL ACN. The samples were

centrifuged to pellet aggregated enzyme and diluted an additional 1:10 with 50 mM KPi buffer pH = 8.0 to ensure the total amine concentration for the Marfey's derivatization was low.

In a microcentrifuge tube, 30 μL of diluted reaction mix (1 equiv., 0.05 mM final total amines from unreacted β-OH amino acid substrate, and formed 1,2-amino alcohol product) was added to a solution of 120 μL of 18.75 mM $NaHCO_3$ (150 equiv., 7.5 mM final concentration $NaHCO_3$) followed by addition of 150 μL 5 mM L-FDVA dissolved in ACN (50 equiv., 2.5 mM final concentration) to bring the total reaction volume to 300 μL. Each reaction was placed in a dark 50 °C thermomixer for 8 h, then quenched with 300 μL of 1:1 ACN:60 mM HCl (15 mM post-quench) before analyzing by UPLC-MS. The yield of the *Rgn*TDC reactions were estimated using the ratio of the derivatized amino alcohol product to the sum of the derivatized amino alcohol product and derivatized amino acid starting material. Total turnover numbers were calculated by taking the yield of the *Rgn*TDC reaction and multiplying it by the max TON (Fig 3e).

Determination of analytical substrate scope yields via Marfey's derivation:

For analytical cascade yields, freeze-thawed ObiH and *Rgn*TDC^{NMY} were spin-filtered, and the supernatants used for the following reactions: 10 mM aldehyde/styrene oxide (**2a-22a**), 50 mM Thr, 400 μM PLP, 40 μM ObiH, 40 μM *Rgn*TDC^{NMY}, and 50 mM KPi buffer pH = 8.0 (final volume of 100 μL). Reactions were conducted in triplicate for 16 h at 37 °C. d Reactions were quenched using 200 μL ACN, followed by 100 μL H_2O, and centrifuged to pellet aggregated enzyme.

A subsequent Marfey's derivatization reaction was performed to assess UPLC yield of the produced 1,2-amino alcohols. To 8 μL of the quenched reaction solution was added 1 μL 20 mM arginine (0.2 mM final concentration), 41 μL saturated $NaHCO_3$ (246 mM final concentration), and 50 μL 10 mM (*R*)-1-fluoro-2-4-dinitrophenyl-5-L-valine amide (D-FDVA, 5

mM final concentration). These reactions proceeded for 16 h at 37 °C. After 16 h, reactions were quenched via addition of 50 µL 1 M HCl and diluted to 1 mL with 1:1 ACN:H_2O. Reaction solutions were analyzed on UPLC-MS by looking at absorbance at 340 nm and comparing relative product peak areas to the derivatized Arg peak area (Fig 4; Fig S12)

Calculation of product enantiomeric ratios via Marfey's derivation:

A Marfey's derivatization reaction was performed to assess the enantiomeric ratios of formed 1,2-amino alcohols following the above procedure with purified material. However, the Marfey's reaction was conducted both in the presence of L-FDVA or D-FDVA to ensure enantiomer separation via our UPLC-MS method. Enantiomeric ratio (e.r.) was determined by Marfey-derivatized product peaks integrated at 340 nm.

Stereoisomer determination of 22c:

Freeze-thawed RgnTDC^{NMY} was spin-filtered, and the supernatant used for the following reactions: 10 mM **22b** (65:35 *anti*:*syn* for the γ-Me and β-OH groups; prepared as previously described,[2] 400 µM PLP, 0 or 10 µM *Rgn*TDC^{NMY}, 50 mM KPi buffer pH = 8.0 (final volume = 100 µL). Reactions were conducted in duplicate for 16 h at 37 °C. Reactions were quenched using 300 µL ACN, followed by 300 µL H_2O, centrifuged to pellet aggregated enzyme, and injected onto UPLC-MS for product detection. Product m/z's were used to identify substrate and product peaks. Selective amino acid stereoisomer conversion was observed only for the minor *syn* amino acid diastereomer along with appearance of a new product peak, which we attribute to be the *syn* 1,2-amino alcohol (Fig S13).

Synthetic Methods

Synthesis of Marfey's Reagent

Synthesis of (*S*)-1-fluoro-2-4-dinitrophenyl-5-L-valine amide (L-FDVA) and (*R*)-1-fluoro-2-4-dinitrophenyl-5-L-valine amide (D-FDVA) was conducted following the procedure of Brückner and Keller-Hoehl (1990) with slight modifications.[5]

(*S*)-1-fluoro-2-4-dinitrophenyl-5-L-valine amide (L-FDVA)

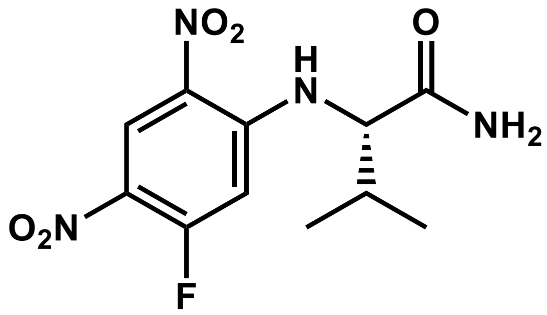

117.9 mg (0.77 mmol) of L-valinamide hydrochloride was dissolved in 3 mL of pH adjusted (addition of ~0.4 mL of 6 M sodium hydroxide to bring the pH from 9 to >10) 0.6 M $NaHCO_3$. To which 7.5 mL of H_2O and 305.6 mg (1.5 mmol) of 1,5-difluoro-2,4-dinitrobenzene (DFDNB) dissolved in 10 mL of acetone were added. The stirring solution was heated at 40°C for 1.5 hr and quenched with 17.5 mL of H_2O.

After the reaction, the insoluble product was isolated by vacuum filtration, washed several times with H_2O, and dried under vacuum. The product was isolated as a yellow crystalline powder (170.8 mg) in a 74% yield. Product purity analyzed by ^{1}H NMR was determined to be 96%. Acetone (2.08 ppm) and H_2O (3.33 ppm) were determined to be the main impurities.

(*R*)-1-fluoro-2-4-dinitrophenyl-5-L-valine amide (D-FDVA)

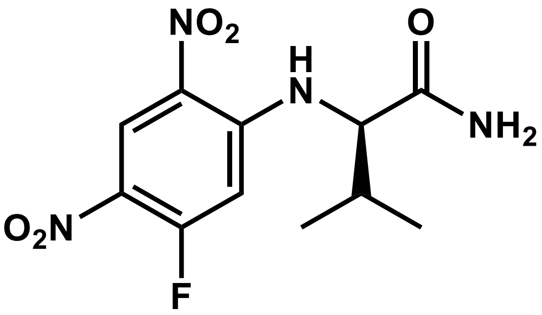

119.0 mg (0.78 mmol) of D-valinamide hydrochloride was dissolved in 3 mL of pH adjusted (addition of ~0.4 mL of 6 M sodium hydroxide to bring the pH from 9.0 to >10) 0.6 M $NaHCO_3$. To which, 7.5 mL of H_2O and 303.5 mg (1.5 mmol) of DFDNB dissolved in 10 mL of acetone were added. The stirring solution was heated at 40°C for 1.5 hr and quenched with 17.5 mL of H_2O.

After the reaction, the insoluble product was isolated by vacuum filtration, washed several times with H_2O, and dried under vacuum. The product was isolated as a yellow crystalline powder (153.3 mg) in a 65% yield. Product purity analyzed by ^{1}H NMR was determined to be 84%. Acetone (2.08 ppm) and H_2O (3.33 ppm) were determined to be the main impurities.

Biocatalytic cascade synthesis of 1,2-amino alcohols

*****We found that purification of the protonated amines was generally easier than purification of the deprotonated amines on C18. We additionally observed high Tris (buffer) retention under basic purification conditions, whereas Tris eluted early as a single peak under acidic conditions. For these reasons, product isolations from C18 were generally performed at pH < 1 via acidification using HCl. We still occasionally observed remaining Tris in isolated product (singlet at 3.6 ppm) and Thr (doublet at 1.4 ppm).

Although acid/base extraction was used for the purification of several of the below compounds, considering the surprising simplicity of C18 purification and high resulting compound purity, we opted for column chromatography purification of many products.

Additionally, since *Rgn*TDC^{H120N} was equally active on phenylserine amino acids, we used this enzyme for many syntheses instead of *Rgn*TDC^{NMY}, simply due to catalyst availability.

(*R*)-2-amino-1-(4-bromophenyl)ethan-1-ol (6c)

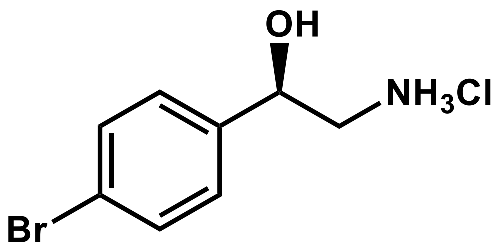

94.7 mg (0.512 mmol) of 4-bromobenzaldehyde was dissolved in 2.5 mL of MeOH. To this, 440 mg (3.7 mmol) of Thr was added, and the solution was diluted to ~45 mL with 50 mM Tris-HCl buffer pH = 8.01. 0.5 mL of 20 mM PLP was then added, such that the final concentration was 0.200 µM. Freeze-thawed ObiH and *Rgn*TDC^{H120N} was spin-filtered, and the supernatants added to the flask with final concentrations of: [ObiH] = 9 µM (0.1 mol% catalyst); [*Rgn*TDC^{H120N}] = 9 µM (0.1 mol% catalyst). Final [aldehyde] = 10 mM; final [Thr] = 74 mM. The reaction was then incubated at 37 °C for 40 h.

The reaction solution was then heat-treated at 75 °C for 20 minutes to aggregate protein, diluted with 40 mL ACN, and centrifuged at 4000 x*g* for 10 min. The pellet was washed with 10 mL 1:1 ACN:H_2O. The combined supernatants were concentrated via rotary evaporation down to ~5 mL, and 6 M HCl was added to bring the pH < 1. The resulting suspension was injected onto a 30 g C18 flash chromatography column and the product eluted early at 1% MeOH. Product-containing fractions were pooled and concentrated via rotary evaporation, frozen @ -80 °C, and lyophilized until dry.

Later fractions were observed to contain additional product. These fractions were pooled and concentrated via rotary evaporation. This aqueous solution was then basified with 6 M NaOH and extracted twice with 50 mL EtOAc. The desired product was then extracted from the EtOAc

layer twice with 50 mL dilute HCl, which were subsequently combined and concentrated via rotary evaporation. This solution was then frozen @ -80 °C and lyophilized until dry.

From both purifications, the product was isolated as the HCl salt as a white powder (82 mg: 67 mg from C18, 14 mg from extraction) in 63% yield. Enantiopurity was determined by subsequent Marfey's analysis to be 99:1 e.r. (*R:S*).

^{1}H NMR (500 MHz, MeOD) δ 7.60 – 7.52 (m, 2H), 7.42 – 7.34 (m, 2H), 4.88 (dd, *J* = 9.6, 3.3 Hz, 1H), 3.17 (dd, *J* = 12.8, 3.3 Hz, 1H), 3.00 (dd, *J* = 12.8, 9.6 Hz, 1H).

^{13}C NMR (126 MHz, MeOD) δ 140.34, 131.46, 127.54, 121.65, 68.92, 45.66.

MS/ESI m/z for $[M+H]^+$; C8H10BrNO; calculated 216.0019, observed 216.0018.

(*R*)-2-amino-1-(4-nitrophenyl)ethan-1-ol (7c)

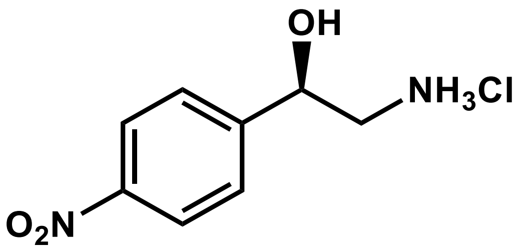

82.8 mg (0.548 mmol) of 4-nitrobenzaldehyde was dissolved in 1.25 mL of methanol (MeOH). To this, 261 mg (2.2 mmol) of Thr was added, and the solution was diluted to ~45 mL with 50 mM Tris-HCl buffer pH = 8.01. 1 mL of 20 mM PLP was then added, such that the final concentration was 0.400 μM. Freeze-thawed ObiH and *Rgn*TDC^{H120N} was spin-filtered, and the supernatants added to the flask with final concentrations of: [ObiH] = 40 μM (0.4 mol% catalyst); [*Rgn*TDC^{H120N}] = 10 μM (0.1 mol% catalyst). Final [aldehyde] = 11 mM; final [Thr] = 44 mM. The reaction was then incubated at 37 °C for 16 h @ 180 rpm.

The reaction solution was then heat-treated at 75 °C for 20 minutes to aggregate protein, diluted with 50 mL ACN, and centrifuged at 4000 x*g* for 10 min. The pellet was washed with 10 mL 1:1 ACN:H_2O. The combined supernatants were concentrated via rotary evaporation down to ~5 mL, and 6 M HCl was added to bring the pH < 1. The resulting suspension was injected onto a 30 g C18 flash chromatography column and the product eluted early at 1% MeOH. Product-containing fractions were pooled and concentrated via rotary evaporation, frozen @ -80 °C, and lyophilized until dry. The product was isolated as the HCl salt as a white powder (105 mg) in 96% yield. Enantiopurity was determined by subsequent Marfey's analysis to be 97.5:2.5 e.r. (*R:S*).

^{1}H NMR (500 MHz, MeOD) δ 8.31 – 8.24 (m, 2H), 7.73 – 7.67 (m, 2H), 5.03 (dd, *J* = 9.4, 3.3 Hz, 1H), 3.24 (dd, *J* = 12.8, 3.3 Hz, 1H), 3.03 (dd, *J* = 12.8, 9.4 Hz, 1H).

^{13}C NMR (126 MHz, MeOD) δ 149.80, 149.33, 128.18, 124.80, 70.12, 46.83.

MS/ESI m/z for $[M+H]^+$; C8H10N2O3; calculated 183.0764, observed 183.0763

***(R)*-N-(4-(2-amino-1-hydroxyethyl)phenyl)methanesulfonamide (8c)**

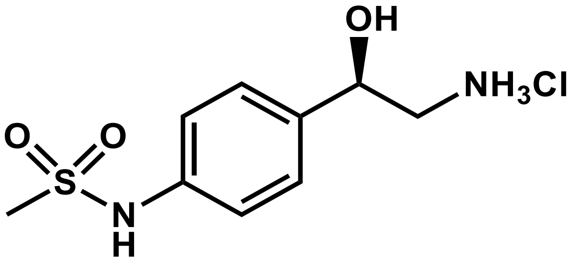

96.6 mg (0.485 mmol) of 4-sulfonamidobenzaldehyde was dissolved in 2.5 mL of MeOH. To this, 433 mg (3.6 mmol) of Thr was added, and the solution was diluted to ~45 mL with 50 mM Tris-HCl buffer pH = 8.01. 1 mL of 20 mM PLP was then added, such that the final concentration was 0.400 μM. Freeze-thawed ObiH and *Rgn*TDC^{NMY} was spin-filtered, and the

supernatants added to the flask with final concentrations of: [ObiH] = 50 μM (0.5 mol% catalyst); [*Rgn*TDC^{NMY}] = 14 μM (0.1 mol% catalyst). Final [aldehyde] = 10 mM; final [Thr] = 73 mM. The reaction was then incubated at 37 °C for 40 h @ 180 rpm.

The reaction solution was then heat-treated at 75 °C for 20 minutes to aggregate protein, diluted with 50 mL ACN, and centrifuged at 4000 xg for 10 min. The supernatant was concentrated via rotary evaporation down to ~5 mL, and 6 M HCl was added to bring the pH < 1. The resulting suspension was injected onto a 30 g C18 flash chromatography column and the product eluted at 1% MeOH. Product-containing fractions were pooled and concentrated via rotary evaporation, frozen @ -80 °C, and lyophilized until dry. The product was isolated as the HCl salt as a white crystalline powder (96 mg) in 74 % yield. Enantiopurity was determined by subsequent Marfey's analysis to be 96:4 e.r. (*R*).

^{1}H NMR (500 MHz, MeOD) δ 7.47 – 7.39 (m, 2H), 7.33 – 7.27 (m, 2H), 4.89 (dd, *J* = 9.6, 3.4 Hz, 1H), 3.16 (dd, *J* = 12.8, 3.4 Hz, 1H), 3.02 (dd, *J* = 12.7, 9.6 Hz, 1H), 2.97 (s, 3H).

^{13}C NMR (126 MHz, MeOD) δ 138.21, 137.12, 126.72, 120.26, 69.05, 47.09, 45.82, 37.88.

MS/ESI m/z for $[M+H]^+$; $C_9H_{14}N_2O_3S$; calculated 231.0798, observed 231.0795.

(*R*)-2-amino-1-(4-fluoro-3-nitrophenyl)ethan-1-ol (9c)

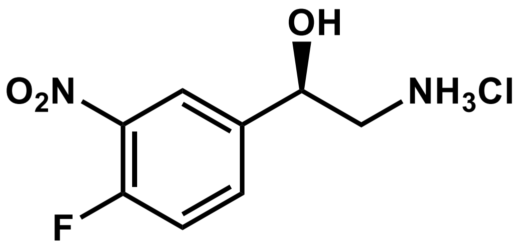

83.7 mg (0.495 mmol) of 4-fluoro-3-nitrobenzaldehyde was dissolved in 2.5 mL of MeOH. To this, 425 mg (3.6 mmol) of Thr was added, and the solution was diluted to ~45 mL with 50 mM KPi buffer pH = 8.08. 1 mL of 20 mM PLP was then added, such that the final concentration was 0.400 µM. Freeze-thawed ObiH and *Rgn*TDC^{NMY} was spin-filtered, and the supernatants added to the flask with final concentrations of: [ObiH] = 12 µM (0.1 mol% catalyst); [*Rgn*TDC^{NMY}] = 17 µM (0.2 mol% catalyst). Final [aldehyde] = 10 mM; final [Thr] = 71 mM. The reaction was then incubated at 37 °C for 16 h @ 140 rpm.

The reaction solution was then heat-treated at 75 °C for 20 minutes to aggregate protein, diluted with 40 mL ACN, and centrifuged at 4000 xg for 10 min. The supernatant was concentrated via rotary evaporation down to ~5 mL, and 6 M HCl was added to bring the pH < 1. The resulting suspension was injected onto a 30 g C18 flash chromatography column and the product eluted at 1% MeOH. Product-containing fractions were pooled and concentrated via rotary evaporation, frozen @ -80 °C, and lyophilized until dry. The product was isolated as the HCl salt as a white crystalline powder (80 mg) in 69 % yield. Enantiopurity was determined by subsequent Marfey's analysis to be 95.5:4.5 e.r. (*R*).

^{1}H NMR (500 MHz, MeOD) δ 8.22 (dd, *J* = 7.1, 2.3 Hz, 1H), 7.83 (ddd, *J* = 8.9, 4.3, 2.3 Hz, 1H), 7.49 (dd, *J* = 10.9, 8.6 Hz, 1H), 5.01 (dd, *J* = 9.2, 3.4 Hz, 1H), 3.25 (dd, *J* = 12.9, 3.4 Hz, 1H), 3.05 (dd, *J* = 12.9, 9.2 Hz, 1H).

^{13}C NMR (126 MHz, MeOD) δ 155.87, 153.78, 138.68, 138.64, 133.02, 132.95, 123.30, 123.28, 118.44, 118.27, 67.93, 45.42.

^{19}F NMR (377 MHz, MeOD) δ -121.80 (ddd, *J* = 11.4, 7.2, 4.2 Hz).

MS/ESI m/z for $[M+H]^+$; C8H9FN2O3; calculated 201.0670, observed 201.0669.

(*R*)-2-amino-1-(quinolin-3-yl)ethan-1-ol (10c)

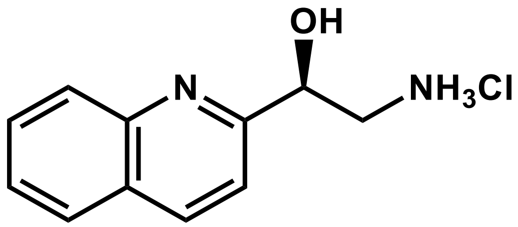

79.5 mg (0.506 mmol) of quinoline-2-carboxaldehyde was dissolved in 2.5 mL of MeOH. To this, 420 mg (3.5 mmol) of Thr was added, and the solution was diluted to ~45 mL with 50 mM Tris-HCl buffer pH = 8.01. 1 mL of 20 mM PLP was then added, such that the final concentration was 0.400 μM. Freeze-thawed ObiH and *Rgn*TDC^{H120N} was spin-filtered, and the supernatants added to the flask with final concentrations of: [ObiH] = 63 μM (0.6 mol% catalyst); [*Rgn*TDC^{H120N}] = 40 μM (0.4 mol% catalyst). Final [aldehyde] = 10 mM; final [Thr] = 70 mM. The reaction was then incubated at 37 °C for 40 h @ 140 rpm.

The reaction solution was then heat-treated at 75 °C for 20 minutes to aggregate protein, diluted with 40 mL ACN, and centrifuged at 4000 xg for 10 min. The supernatant was concentrated via rotary evaporation down to ~3 mL, and 6 M HCl was added to bring the pH < 1. The resulting suspension was injected onto a 30 g C18 flash chromatography column and the product eluted at 1% MeOH. Product-containing fractions were pooled and concentrated via rotary evaporation, frozen @ -80 °C, and lyophilized until dry.

Later fractions were observed to contain significant product. These fractions were pooled and concentrated via rotary evaporation. This aqueous solution was then basified with 6 M NaOH and extracted thrice with 50 mL EtOAc. The desired product was then extracted from the EtOAc layer thrice with 50 mL dilute HCl, which were subsequently combined and concentrated via rotary evaporation. This solution was then frozen @ -80 °C and lyophilized until dry.

From both purifications, the product was isolated as the HCl salt as a yellow solid (104 mg: 66 mg from C18, 37 mg from extraction) in 91% yield. Enantiopurity was determined by subsequent Marfey's analysis to be 95.5:4.5 e.r. (*R*).

^{1}H NMR (500 MHz, MeOD) δ 8.84 (d, *J* = 8.6 Hz, 1H), 8.30 (d, *J* = 8.5 Hz, 1H), 8.19 (dd, *J* = 8.4, 1.4 Hz, 1H), 8.06 – 7.98 (m, 2H), 7.83 (ddd, *J* = 8.2, 6.9, 1.1 Hz, 1H), 5.42 (dd, *J* =f 8.6, 3.7 Hz, 1H), 3.58 (dd, *J* = 13.0, 3.8 Hz, 1H), 3.37 (dd, *J* = 13.0, 8.6 Hz, 1H).

^{13}C NMR (126 MHz, MeOD) δ 159.30, 142.73, 142.55, 132.57, 128.40, 128.36, 124.25, 119.05, 68.53, 43.99, 19.36.

MS/ESI m/z for [M+H]+ ; $C_{11}H_{12}N_2O$; calculated 189.1022, observed 189.1021.

(*R*)-2-amino-1-(naphthalen-2-yl)ethan-1-ol (11c)

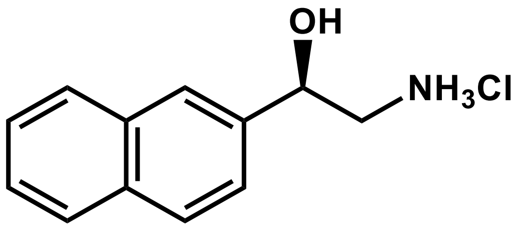

84.4 mg (0.5 mmol) of 2-naphthaldehyde was dissolved in 5 mL of MeOH. To this, 298 mg (2.5 mmol) of Thr was added, and the solution was diluted to ~45 mL with 50 mM Tris-HCl buffer pH = 8.01. 1 mL of 20 mM PLP was then added, such that the final concentration was 0.400 μM.

Freeze-thawed ObiH and *Rgn*TDC^{H120N} was spin-filtered, and the supernatants added to the flask with final concentrations of: [ObiH] = 40 μM (0.4 mol% catalyst); [*Rgn*TDC^{H120N}] = 10 μM (0.1 mol% catalyst). Final [aldehyde] = 10 mM; final [Thr] = 50 mM. The reaction was then incubated at 37 °C for 40 h @ 180 rpm. Insoluble 2-naphthaldehyde was visible at the bottom of the flask throughout the reaction.

The reaction solution was then heat-treated at 75 °C for 20 minutes to aggregate protein, diluted with 50 mL ACN, and centrifuged at 4000 xg for 10 min. The pellet was washed with 10 mL 1:1 ACN:H_2O. The combined supernatants were concentrated via rotary evaporation down to ~5 mL, and 6 M HCl was added to bring the pH < 1. The resulting suspension was injected onto a 30 g C18 flash chromatography column and the product eluted at 20% MeOH. Product-containing fractions were pooled and concentrated via rotary evaporation, frozen @ -80 °C, and lyophilized until dry. The product was isolated as the HCl salt as a white powder (55 mg) in 49% yield. Enantiopurity was determined by subsequent Marfey's analysis to be 95:5 e.r. (*R*).

^{1}H NMR (500 MHz, MeOD) δ 7.97 – 7.81 (m, 4H), 7.58 – 7.42 (m, 3H), 5.06 (dd, *J* = 9.4, 3.4 Hz, 1H), 3.25 (dd, *J* = 12.8, 3.4 Hz, 1H), 3.12 (dd, *J* = 12.8, 9.4 Hz, 1H).

^{13}C NMR (126 MHz, MeOD) δ 138.33, 133.43, 133.39, 128.21, 127.61, 127.36, 126.07, 125.92, 124.63, 123.26, 69.66, 45.83.

MS/ESI m/z for $[M+H]^+$; C12H13NO; calculated 188.1070, observed 188.1070.

(*R*)-1-([1,1'-biphenyl]-4-yl)-2-aminoethan-1-ol (12c)

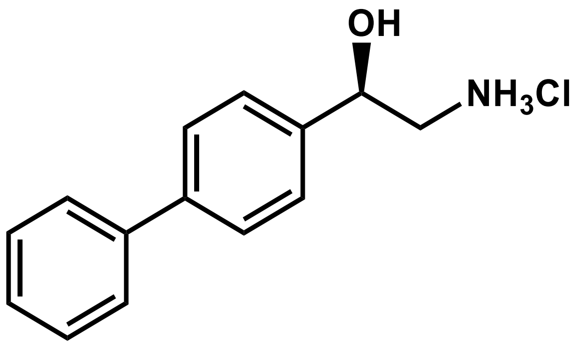

89.3 mg (0.490 mmol) of biphenyl-4-carboxaldehyde was dissolved in 2.5 mL of MeOH. To this, 426 mg (3.6 mmol) of Thr was added, and the solution was diluted to ~45 mL with 50 mM Tris-HCl buffer pH = 8.01. 1 mL of 20 mM PLP was then added, such that the final concentration was 0.400 μM. Freeze-thawed ObiH and *Rgn*TDC^{NMY} was spin-filtered, and the supernatants added to the flask with final concentrations of: [ObiH] = 12 μM (0.1 mol% catalyst); [*Rgn*TDC^{NMY}] = 11 μM (0.1 mol% catalyst). Final [aldehyde] = 10 mM; final [Thr] = 72 mM. The reaction was then incubated at 37 °C for 16 h @ 140 rpm.

The reaction solution was then heat-treated at 75 °C for 20 minutes to aggregate protein, diluted with 40 mL ACN, and centrifuged at 4000 xg for 10 min. It was observed that product and remaining starting aldehyde also crashed out at this stage. Therefore, the pellet was extracted with hot MeOH and 1 M HCl, solvent was rotary evaporated, and the remaining solution was injected onto a 30 g C18 flash chromatography column. The product eluted at 70% MeOH. Product-containing fractions were pooled and concentrated via rotary evaporation, frozen @ -80 °C, and lyophilized until dry.

The supernatant of the original spin-down was concentrated via rotary evaporation down to ~3 mL, and 6 M HCl was added to bring the pH < 1. The resulting suspension was injected onto a 30 g C18 flash chromatography column and the product eluted at 10% MeOH. Product-

containing fractions were pooled and concentrated via rotary evaporation, frozen @ -80 °C, and lyophilized until dry.

From both purifications, the product was isolated as the HCl salt as a white powder (23 mg: 14 mg from C18, 8 mg from extraction) in 21% yield. Enantiopurity was determined by subsequent Marfey's analysis to be 92:8 e.r. (*R*).

^{1}H NMR (500 MHz, MeOD) δ 7.73 – 7.58 (m, 4H), 7.57 – 7.49 (m, 2H), 7.49 – 7.41 (m, 2H), 7.40 – 7.33 (m, 1H), 4.96 (dd, *J* = 9.5, 3.4 Hz, 1H), 3.21 (dd, *J* = 12.7, 3.4 Hz, 1H), 3.08 (dd, *J* = 12.7, 9.6 Hz, 1H).

^{13}C NMR (126 MHz, MeOD) δ 141.22, 140.47, 139.98, 128.52, 127.15, 126.92, 126.54, 126.09, 69.32, 45.90.

MS/ESI m/z for [M+H]+ ; $C_{14}H_{15}NO$; calculated 214.1226, observed 214.1223.

(*R*)-1-aminoheptan-2-ol (5c)

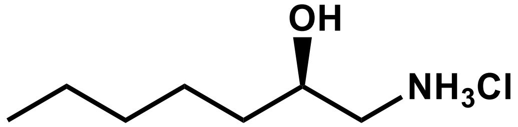

50.0 mg (0.5 mmol) of hexanal was dissolved in 1 mL of MeOH. To this, 180 mg (1.5 mmol) of Thr was added, and the solution was diluted to ~14 mL with 50 mM KPi buffer pH = 8.01. 0.4 mL of 20 mM PLP was then added, such that the final concentration was 0.400 μM. Freeze-thawed ObiH and *Rgn*TDC^{NMY} was spin-filtered, and the supernatants added to the flask with final concentrations of: [ObiH] = 33 μM (0.1 mol% catalyst); [*Rgn*TDC^{NMY}] = 33 μM (0.1 mol% catalyst). Final [aldehyde] = 25 mM; final [Thr] = 76 mM. The reaction was then incubated at 37 °C for 40 h with no shaking.

The reaction solution was then heat-treated at 75 °C for 20 minutes to aggregate protein, diluted with 50 mL ACN, and centrifuged at 4000 xg for 10 min. The pellet was washed with 20 mL 1:1 ACN:H_2O. The combined supernatants were concentrated via rotary evaporation down to ~20 mL. The pH of the solution was adjusted via addition of 6 M NaOH to > 11 followed by 6x extraction with 50 mL EtOAc. The organic extractions were combined and evaporated to ~50 mL. The product was then extracted from the organic layer via addition of 3x 50 mL dilute HCl (pH ~3). The resulting aqueous layer was concentrated via rotary evaporation and was transferred to a pre-tared flask, frozen @ -80 °C, and lyophilized until dry. The product was isolated as the HCl salt as a white powder (68 mg) in 81% yield. Enantiopurity was determined by subsequent Marfey's analysis to be > 99:1 e.r. (*R*).

^{1}H NMR (500 MHz, MeOD) δ 3.76 (dtd, *J* = 9.2, 6.1, 3.1 Hz, 1H), 3.03 (dd, *J* = 12.7, 3.1 Hz, 1H), 2.77 (dd, *J* = 12.7, 9.4 Hz, 1H), 1.56 – 1.27 (m, 8H), 0.98 – 0.91 (m, 3H).

^{13}**C NMR** (126 MHz, MeOD) δ 67.36, 44.69, 34.59, 31.45, 24.66, 22.20, 12.93.

MS/ESI m/z for $[M+H]^+$; C7H17NO; calculated 132.1383, observed 132.1382.

(*R*)-1-amino-4-(methylthio)butan-2-ol (14c)

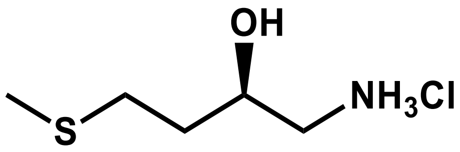

52.1 mg (0.500 mmol) of methional was dissolved in 1 mL of MeOH. To this, 181 mg (1.5 mmol) of Thr was added, and the solution was diluted to ~12 mL with 50 mM KPi buffer pH = 8.08. 200 µL of 20 mM PLP was then added, such that the final concentration was 0.200 µM. Freeze-thawed ObiH and *Rgn*TDC^{NMY} was spin-filtered, and the supernatants added to the flask with final concentrations of: [ObiH] = 25 µM (0.1 mol% catalyst); [*Rgn*TDC^{NMY}] = 50 µM (0.2 mol% catalyst). Final [aldehyde] = 25 mM; final [Thr] = 76 mM. The reaction was then incubated at 37 °C for 36 h @ 200 rpm.

The reaction solution was then heat-treated at 75 °C for 20 minutes to aggregate protein, diluted with 25 mL ACN, and centrifuged at 4000 xg for 10 min. The supernatant was concentrated via rotary evaporation down to ~15 mL. The pH of the solution was adjusted via addition of 6 M NaOH to > 11 followed by 5x extraction with 50 mL EtOAc. The organic extractions were combined and evaporated to ~50 mL. The product was then extracted from the organic layer via addition of 5x 50 mL dilute HCl (pH ~3). The resulting aqueous layer was concentrated via rotary evaporation and was transferred to a pre-tared flask, frozen @ -80 °C, and lyophilized until dry. The product was isolated as the HCl salt as a white crystalline powder (25 mg) in 29% yield. Enantiopurity was determined by subsequent Marfey's analysis to be > 99:1 e.r. (*R*).

^{1}**H NMR** (500 MHz, MeOD) δ 3.94 (tt, *J* = 7.9, 3.5 Hz, 1H), 3.11 – 3.02 (m, 1H), 2.83 (dd, *J* = 12.6, 9.2 Hz, 1H), 2.72 – 2.57 (m, 2H), 2.12 (s, 3H), 1.77 (hd, *J* = 7.4, 3.7 Hz, 2H).

^{13}**C NMR** (126 MHz, MeOD) δ 66.13, 44.64, 33.94, 29.30, 13.92.

MS/ESI m/z for [M+H]+ ; $C_5H_{13}NOS$; calculated 136.0791, observed 136.0789.

tert-butyl (*S*)-(3-amino-2-hydroxypropyl)carbamate (15c)

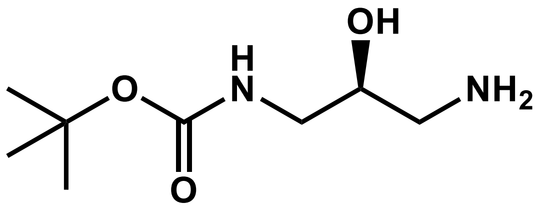

80.6 mg (0.506 mmol) of N-Boc aminoacetaldehyde was dissolved in 1 mL of MeOH. *This substrate was a waxy, sticky solid and did not dissolve easily into solution. To this, 180 mg (1.5 mmol) of Thr was added, and the solution was diluted to ~12 mL with 50 mM Tris-HCl buffer pH = 8.01. 400 µL of 20 mM PLP was then added, such that the final concentration was 0.400 µM. Freeze-thawed ObiH and *Rgn*TDC^{NMY} was spin-filtered, and the supernatants added to the flask with final concentrations of: [ObiH] = 127 µM (0.5 mol% catalyst); [*Rgn*TDC^{NMY}] = 101 µM (0.4 mol% catalyst). Final [aldehyde] = 25 mM; final [Thr] = 76 mM. The reaction was then incubated at 37 °C for 36 h @ 140 rpm.

The reaction solution was then heat-treated at 75 °C for 20 minutes to aggregate protein, diluted with 25 mL ACN, and centrifuged at 4000 xg for 10 min. The supernatant was concentrated via rotary evaporation down to ~4 mL. Due to the present Boc group, we opted not to use acid/base extraction, and instead injected directly on a 30 g C18 flash chromatography column. The product eluted at 100% MeOH with a small amount of aldehyde starting material. Product-containing fractions were pooled and concentrated via rotary evaporation, frozen @ -80 °C, and

lyophilized until dry. The product was isolated as a tan powder (8.5 mg) in 7% yield. Enantiopurity was determined by subsequent Marfey's analysis to be > 99:1 e.r. (*R*).

^{1}H NMR (500 MHz, MeOD) δ 3.62 (dtd, *J* = 7.3, 5.9, 4.0 Hz, 1H), 3.17 – 3.05 (m, 2H), 2.72 (dd, *J* = 13.3, 4.1 Hz, 1H), 2.58 (dd, *J* = 13.2, 7.3 Hz, 1H), 1.46 (s, 9H).

^{13}C NMR (126 MHz, MeOD) δ 157.35, 78.80, 70.98, 44.10, 43.39, 27.31.

MS/ESI m/z for $[M+H]^+$; $C_8H_{18}N_2O_3$; calculated 191.1390, observed 191.1387.

(*R*)-1-amino-3-phenylpropan-2-ol (3c)

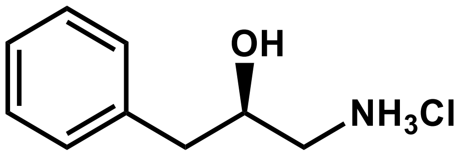

60.1 mg (0.5 mmol) of styrene oxide was dissolved in 2.5 mL of MeOH. To this, 304 mg (2.55 mmol) of Thr was added, and the solution was diluted to ~45 mL with 50 mM KPi buffer pH = 8.01. 1 mL of 20 mM PLP was then added, such that the final concentration was 0.400 µM. 50 mg frozen SOI cells were then added to the flask. Freeze-thawed ObiH and *Rgn*TDC^{NMY} was spin-filtered, and the supernatants added to the flask with final concentrations of: [ObiH] = 10 µM (0.1 mol% catalyst); [*Rgn*TDC^{NMY}] = 22 µM (0.2 mol% catalyst). Final [epoxide] = 10 mM; final [Thr] = 51 mM. The reaction was then incubated at 37 °C for 40 h @ 180 rpm.

The reaction solution was then heat-treated at 75 °C for 20 minutes to aggregate protein, diluted with 50 mL ACN, and centrifuged at 4000 xg for 10 min. The pellet was washed with 10 mL 1:1 ACN:H_2O. The combined supernatants were concentrated via rotary evaporation down to ~40 mL. The pH of the solution was adjusted via addition of 6 M NaOH to > 11 followed by 5x

extraction with 75 mL EtOAc. The organic extractions were combined and evaporated to ~50 mL. The product was then extracted from the organic layer via addition of 3x 50 mL dilute HCl (pH ~3). The resulting aqueous layer was concentrated via rotary evaporation and was injected onto a 30 g C18 flash chromatography column. The product eluted at 1% MeOH. Product-containing fractions were pooled and concentrated via rotary evaporation, frozen @ -80 °C, and lyophilized until dry. The product was isolated as the HCl salt as a white crystalline solid (25mg) in 33% yield. Enantiopurity was determined by subsequent Marfey's analysis to be > 99:1 e.r. (*R*).

^{1}H NMR (500 MHz, MeOD) δ 7.36 – 7.21 (m, 5H), 4.03 (dtd, *J* = 9.7, 6.6, 2.9 Hz, 1H), 3.01 (dd, *J* = 12.8, 3.0 Hz, 1H), 2.91 – 2.75 (m, 3H).

^{13}C NMR (126 MHz, MeOD) δ 137.24, 129.09, 128.19, 126.31, 68.66, 68.62, 44.07, 41.25.

MS/ESI m/z for $[M+H]^+$; C9H13NO; calculated 188.1070, observed 188.1070.

(*R*)-1-amino-3-(benzo[d][1,3]dioxol-5-yl)propan-2-ol (20c)

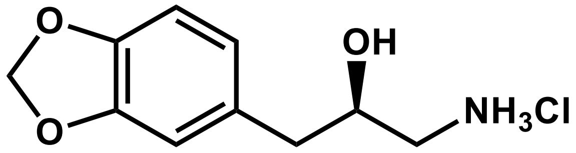

55 mg (0.328 mmol) of 5-(oxiran-2-yl)benzo[d][1,3]dioxole (as prepared by Meza *et al.*[2]) was dissolved in 2.5 mL of MeOH. To this, 423 mg (3.55 mmol) of Thr was added, and the solution was diluted to ~45 mL with 50 mM KPi buffer pH = 8.01. 1 mL of 20 mM PLP was then added, such that the final concentration was 0.400 μM. 50 mg frozen SOI cells were then added to the flask. Freeze-thawed ObiH and *Rgn*TDC^{NMY} was spin-filtered, and the supernatants added to the flask with final concentrations of: [ObiH] = 54 μM (0.6 mol% catalyst); [*Rgn*TDC^{NMY}] = 36 μM (0.5 mol% catalyst). Final [epoxide] = 10 mM; final [Thr] = 71 mM. The reaction was then incubated at 37 °C for 40 h @ 140 rpm.

The reaction solution was then heat-treated at 75 °C for 20 minutes to aggregate protein, diluted with 50 mL ACN, and centrifuged at 4000 xg for 10 min. The pellet was washed with 10 mL 1:1 ACN:H_2O. The combined supernatants were concentrated via rotary evaporation down to ~3 mL. The remaining liquid was injected onto a 30 g C18 flash chromatography column. The product eluted cleanly at 1% MeOH as well as in a mixture at 30%. The early product-containing fractions were pooled and concentrated via rotary evaporation, frozen @ -80 °C, and lyophilized until dry.

Later fractions were purified via acid/base extraction. These fractions were pooled and concentrated via rotary evaporation to remove MeOH. The pH of the solution was adjusted via addition of 6 M NaOH to > 11 followed by 3x extraction with 50 mL EtOAc. The organic extractions were combined and evaporated to ~50 mL. The product was then extracted from the organic layer via addition of 3x 50 mL dilute HCl (pH ~3). This aqueous solution was

concentrated via rotary evaporation, frozen @ -80 °C, and lyophilized until dry. The product was isolated as the HCl salt as a white solid (20 mg: 4 mg from early flash chromatography fractions, 17 mg from extraction) in 27% yield. Enantiopurity was determined by subsequent Marfey's analysis to be > 99:1 e.r. (*R*).

^{1}H NMR (500 MHz, MeOD) δ 6.79 – 6.76 (m, 2H), 6.72 (dd, *J* = 7.9, 1.7 Hz, 1H), 5.93 (s, 2H), 3.95 (dtd, *J* = 9.7, 6.6, 3.0 Hz, 1H), 2.99 (dd, *J* = 12.7, 3.0 Hz, 1H), 2.81 – 2.68 (m, 3H).

^{13}C NMR (126 MHz, MeOD) δ 147.83, 146.46, 130.83, 122.05, 109.23, 107.78, 100.85, 68.69, 43.96, 40.88.

MS/ESI m/z for $[M+H]^+$; $C_{10}H_{13}NO_3$; calculated 196.0968, observed 196.0967.

(2*R*,3*S*)-1-amino-3-phenylbutan-2-ol (21c)

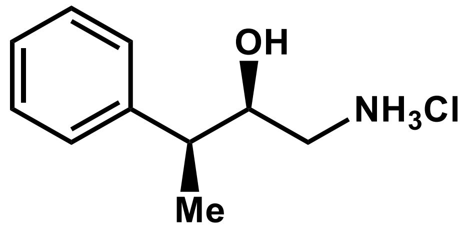

72.5 mg (0.540 mmol) of 2-methyl-2-phenyloxirane was dissolved in 2.5 mL of MeOH. To this, 322 mg (2.7 mmol) of Thr was added, and the solution was diluted to ~45 mL with 50 mM KPi buffer pH = 8.01. 1 mL of 20 mM PLP was then added, such that the final concentration was 0.400 μM. 50 mg frozen SOI cells were then added to the flask. Freeze-thawed ObiH and *Rgn*TDC^{NMY} was spin-filtered, and the supernatants added to the flask with final concentrations of: [ObiH] = 43 μM (0.4 mol% catalyst); [*Rgn*TDC^{NMY}] = 30 μM (0.3 mol% catalyst). Final

[epoxide] = 11 mM; final [Thr] = 54 mM. The reaction was then incubated at 37 °C for 40 h @ 140 rpm.

The reaction solution was then heat-treated at 75 °C for 20 minutes to aggregate protein, diluted with 50 mL ACN, and centrifuged at 4000 xg for 10 min. The combined supernatants were concentrated via rotary evaporation down to ~5 mL. The remaining liquid was injected onto a 30 g C18 flash chromatography column. The product eluted cleanly at 1% MeOH as well as in a mixture at 30%. The early product-containing fractions were pooled and concentrated via rotary evaporation, frozen at -80 °C, and lyophilized until dry.

Later fractions were purified via acid/base extraction. These fractions were pooled and concentrated via rotary evaporation to remove MeOH. The pH of the solution was adjusted via addition of 6 M NaOH to > 11 followed by 4x extraction with 75 mL EtOAc. The organic extractions were combined and evaporated to ~75 mL. The product was then extracted from the organic layer via addition of 4x 75 mL dilute HCl (pH ~3). This aqueous solution was concentrated via rotary evaporation, frozen @ -80 °C, and lyophilized until dry. The product was isolated as the HCl salt as a white solid (43 mg: 25 mg from early flash chromatography fractions, 18 mg from extraction) in 39% yield. Only one diastereomer was observable via ^{1}H NMR. The methyl stereocenter was assigned by extension, as *Rgn*TDC^{NMY} was found to be active only on the corresponding (2*S*, 3R, 4S) amino acid stereoisomer (Fig S13). Enantiopurity was determined by subsequent Marfey's analysis to be > 99:1 e.r. and > 99:1 d.r. (2*R,* 3*S*).

^{1}H NMR (500 MHz, MeOD) δ 7.37 – 7.31 (m, 2H), 7.26 (td, *J* = 6.8, 1.6 Hz, 3H), 3.81 (td, *J* = 9.2, 3.6 Hz, 1H), 2.78 – 2.57 (m, 3H), 1.40 (d, *J* = 7.0 Hz, 3H).

^{13}C NMR (126 MHz, MeOD) δ 143.09, 128.51, 127.17, 126.65, 72.26, 45.09, 43.40, 17.16.

MS/ESI m/z for $[M+H]^{+}$; $C_{10}H_{15}NO$; calculated 166.1226, observed 166.1226.

Chemoenzymatic syntheses

Reductive amination of **7c**, **8c** and **11c** was conducted following the procedure of Tajbakhsh et al. (2011) with slight modifications.[6]

(*R*)-2-(isopropylamino)-1-(4-nitrophenyl)ethan-1-ol (7d)

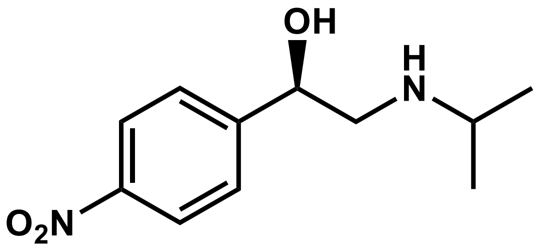

45.4 mg (0.207 mmol) of the (*R*)-2-amino-1-(4-nitrophenyl)ethan-1-ol HCl salt was added to a round bottom along with 0.80 mL acetone (1.1 mmol) and 2 mL trifluoroethanol (TFE). The round bottom was heated to 45 °C upon which 113.49 mg $NaBH_4$ (3 mmol) was added. The solution was then stirred at 45 °C for 16 h.

After the reaction, remaining insoluble $NaBH_4$ was filtered away via washing with MeOH and the filtrate was collected and evaporated via rotary evaporation to ~ 2 mL. The resulting solution was diluted with 3 mL H_2O and injected onto a 30 g C18 flash chromatography column. The product eluted at 80% MeOH. Product-containing fractions were pooled and concentrated via rotary evaporation, frozen @ -80 °C, and lyophilized until dry. The product was isolated as a white crystalline powder (49 mg) in quantitative yield.

^{1}H NMR (500 MHz, CD_3CN) δ 8.23 – 8.18 (m, 2H), 7.66 – 7.59 (m, 2H), 4.75 (dd, *J* = 8.5, 4.0 Hz, 1H), 2.94 (dd, *J* = 12.2, 4.1 Hz, 1H), 2.81 (hept, *J* = 6.2 Hz, 1H), 2.61 (dd, *J* = 12.2, 8.5 Hz, 1H), 1.05 (dd, *J* = 6.3, 1.8 Hz, 6H).

^{13}C NMR (126 MHz, CD_3CN) δ 152.31, 147.75, 127.40, 123.83, 71.76, 54.84, 48.84, 23.08, 22.76.

MS/ESI m/z for $[M+H]^+$; C11H16N2O3; calculated 225.1234 observed 225.1231.

(*R*)-N-(4-(1-hydroxy-2-(isopropylamino)ethyl)phenyl)methanesulfonamide (8d)

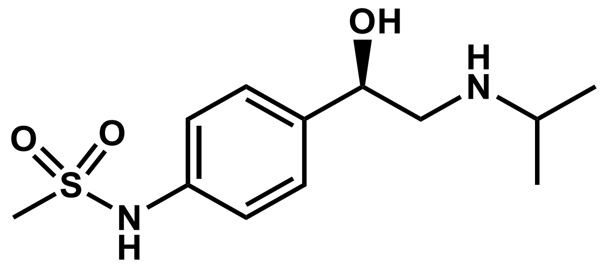

77.5 mg (0.290 mmol) of the (*R*)-N-(4-(2-amino-1-hydroxyethyl)phenyl)-methanesulfonamide HCl salt was added to a round bottom along with 1.05 mL acetone (1.4 mmol) and 3 mL TFE. The round bottom was heated to 45 °C upon which 119.2 mg $NaBH_4$ (3 mmol) was added. The solution was then stirred at 45 °C for 16 h.

After the reaction, remaining insoluble $NaBH_4$ was filtered away via washing with MeOH and the filtrate was collected and evaporated via rotary evaporation to ~ 2 mL. The resulting solution was diluted with 3 mL H_2O and injected onto a 30 g C18 flash chromatography column. The product eluted at 70% MeOH. Product-containing fractions were pooled and concentrated via rotary evaporation, frozen @ -80 °C, and lyophilized until dry. The product was isolated as a white crystalline powder 82 mg) in quantitative yield.

^{1}**H NMR** (500 MHz, MeOD) δ 7.18 (d, *J* = 8.3 Hz, 2H), 7.09 (d, *J* = 8.5 Hz, 2H), 4.65 (t, *J* = 6.7 Hz, 1H), 2.89 – 2.83 (m, 1H), 2.82 (s, 3H), 2.77 – 2.74 (m, 2H), 1.09 (dd, *J* = 16.3, 6.3 Hz, 6H).

^{13}**C NMR** (126 MHz, MeOD) δ 147.91, 147.88, 133.33, 133.31, 126.11, 121.14, 72.15, 54.19, 37.54, 21.31, 21.07.

MS/ESI m/z for $[M+H]^+$; $C_{12}H_{20}N_2O_3S$; calculated 273.1267, observed 273.1264.

(*R*)-2-(isopropylamino)-1-(naphthalen-2-yl)ethan-1-ol (11d)

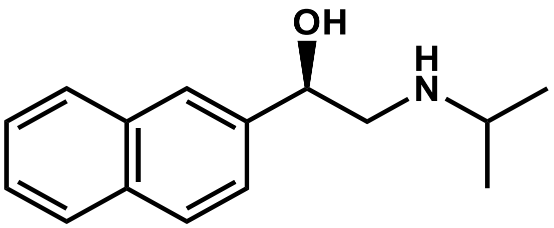

50.0 mg (0.224 mmol) of the (*R*)-2-amino-1-(naphthalen-2-yl)ethan-1-ol HCl salt was added to a round bottom along with 0.22 mL acetone (3 mmol) and 3 mL TFE. The round bottom was heated to 45 °C upon which 127.8 mg $NaBH_4$ (3 mmol) was added. The solution was then stirred at 45 °C for 16 h.

After the reaction, remaining insoluble $NaBH_4$ was filtered away via washing with MeOH and the filtrate was collected and evaporated via rotary evaporation to ~ 0.5 mL. The resulting solution was diluted with 2 mL H_2O and injected onto a 30 g C18 flash chromatography column. The product eluted at 100% MeOH. Product-containing fractions were pooled and concentrated via rotary evaporation, frozen at -80 °C, and lyophilized until dry. The product was isolated as a white crystalline powder (36.5 mg) in 71% yield.

^{1}H NMR (500 MHz, CD_3CN) δ 7.93 – 7.85 (m, 4H), 7.57 – 7.47 (m, 3H), 4.78 (dd, *J* = 8.7, 4.0 Hz, 1H), 2.97 (dd, *J* = 12.1, 4.0 Hz, 1H), 2.83 (hept, *J* = 6.3 Hz, 1H), 2.70 (dd, *J* = 12.1, 8.7 Hz, 1H), 1.06 (d, *J* = 6.2 Hz, 6H).

^{13}C NMR (126 MHz, CD_3CN) δ 142.11, 133.90, 133.40, 128.33, 128.25, 128.12, 126.62, 126.21, 125.02, 124.91, 72.61, 55.21, 48.88, 23.14, 22.85.

MS/ESI m/z for $[M+H]^+$; $C_{15}H_{19}NO$; calculated 230.1537, observed 230.1536.

Small Molecule Crystallography

Product Crystallization

8 mg of 2-amino-1-(4-bromophenyl)ethan-1-ol (**6c**) was dissolved in 0.6 mL of 2:1 MeOH:ACN mixture. The solution was filtered to remove impurities using a 0.2 µm PES syringe filter. The solution was heated at 75 °C until evaporation and slowly cooled to room temperature. Small, clear crystals were observed around the edge of the vial.

Data Collection

A colorless crystal with approximate dimensions 0.057 x 0.036 x 0.011 mm^3 was selected under oil under ambient conditions and attached to the tip of a MiTeGen MicroMount©. The crystal was mounted in a stream of cold nitrogen at 100(1) K and centered in the X-ray beam by using a video camera. The crystal evaluation and data collection were performed on a Bruker D8 VENTURE PhotonIII four-circle diffractometer with Cu Kα (λ = 1.54178 Å) radiation and the detector to crystal distance of 4.0 cm.[3] The initial cell constants were obtained from a 180° φ scan conducted at a 2θ = 50° angle with the exposure time of 1 second per frame. The reflections were successfully indexed by an automated indexing routine built in the APEX3 program. The final cell constants were calculated from a set of 8521 strong reflections from the actual data collection. The data were collected by using the full sphere data collection routine to survey the reciprocal space to the extent of a full sphere to a resolution of 0.78 Å. A total of 10187 data were harvested by collecting 15 sets of frames with 0.5° scans in ω and φ with an exposure time 10– 20 sec per frame. These highly redundant datasets were corrected for Lorentz and polarization effects. The absorption correction was based on fitting a function to the empirical transmission surface as sampled by multiple equivalent measurements.[4]

Structure Solution and Refinement

The systematic absences in the diffraction data were consistent for the space groups $P2_1$ and $P2_1/m$. The E-statistics strongly suggested the non-centrosymmetric space group $P2_1$ that yielded chemically reasonable and computationally stable results of refinement [5-8]. A successful solution by intrinsic phasing provided most non-hydrogen atoms from the E-map. The remaining non-hydrogen atoms were located with an alternating series of least-squares cycles and difference Fourier maps. All nonhydrogen atoms were refined with anisotropic displacement coefficients. All hydrogen atoms (except those bound to non-C atoms) were included in the structure factor calculation at idealized positions and were allowed to ride on the neighboring atoms with relative isotropic displacement coefficients. The absolute configuration of the C2 chiral atom is *R*. The final least-squares refinement of 121 parameters against 2083 data resulted in residuals *R* (based on F^2 for $I \geq 2\sigma$) and *wR* (based on F^2 for all data) of 0.0350 and 0.0960, respectively. The final difference Fourier map was featureless.

CCDC 2207847 contain the supplementary crystallographic data for this paper. These data are provided free of charge by the Cambridge Crystallographic Data Centre (www.ccdc.cam.ac.uk/structures).

Summary

Crystal Data for $[C_8H_{11}BrNO]^+Cl^-$ (M = 252.54 g/mol): monoclinic, space group $P2_1$ (no. 4), a = 5.823(2) Å, b = 7.573(2) Å, c = 11.404(4) Å, β = 102.507(16)°, V = 490.9(3) Å^3, Z = 2, T = 100.0 K, μ(Cu Kα) = 7.840 mm^{-1}, *Dcalc* = 1.708 g/cm^3, 7685 reflections measured (7.94° ≤ 2Θ ≤ 164.192°), 2083 unique (R_{int} = 0.0433, R_{sigma} = 0.0376) which were used in all calculations. The final R_1 was 0.0350 (I > 2σ(I)) and wR_2 was 0.0960 (all data).

Supplementary Figures

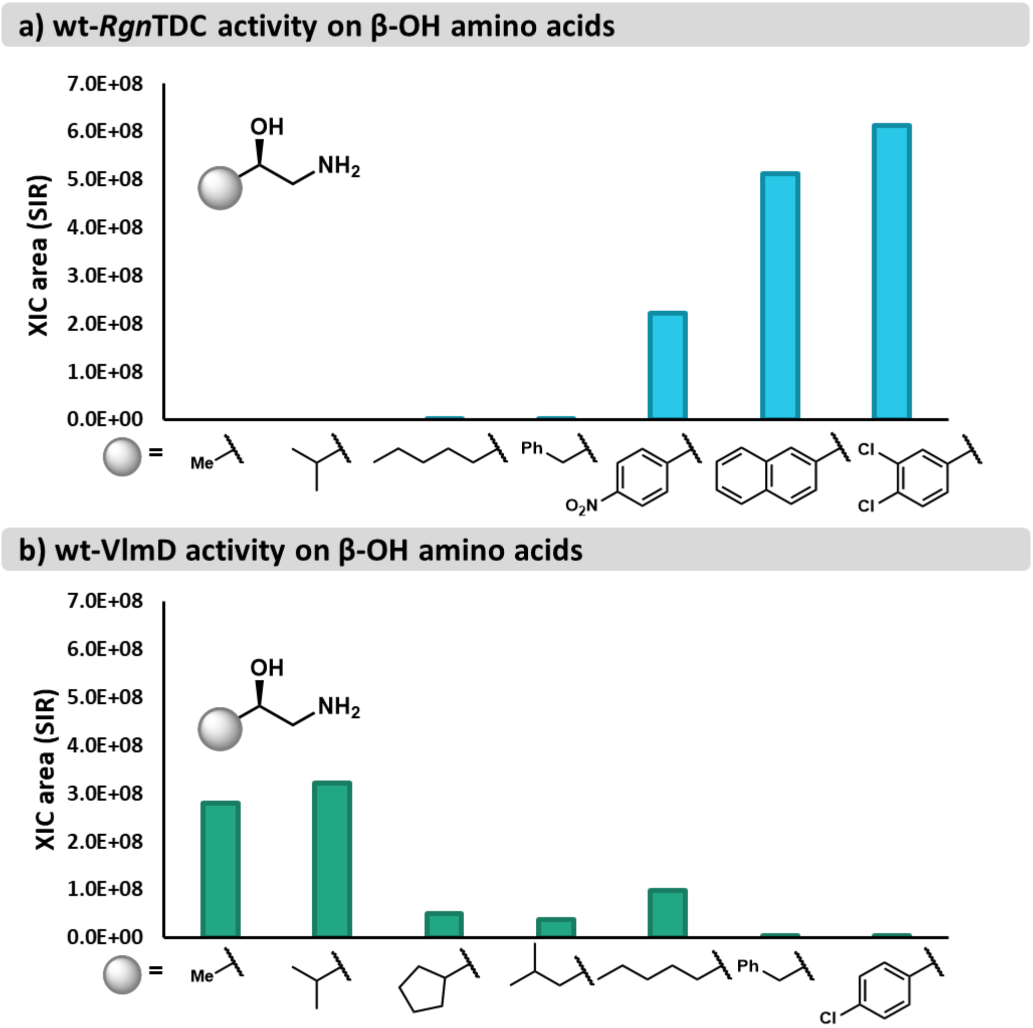

Figure S1. Activity of *Rgn*TDC and VlmD with various β-OH amino acids. 10 mM amino acid, 500 μM PLP, 50 mM KPi buffer pH = 8.0, and 37.5 μM *Rgn*TDC (a)/VlmD (b) (final volume = 100 μL). Reactions were allowed to proceed for 16 h at 37 °C. XIC = extracted ion count.

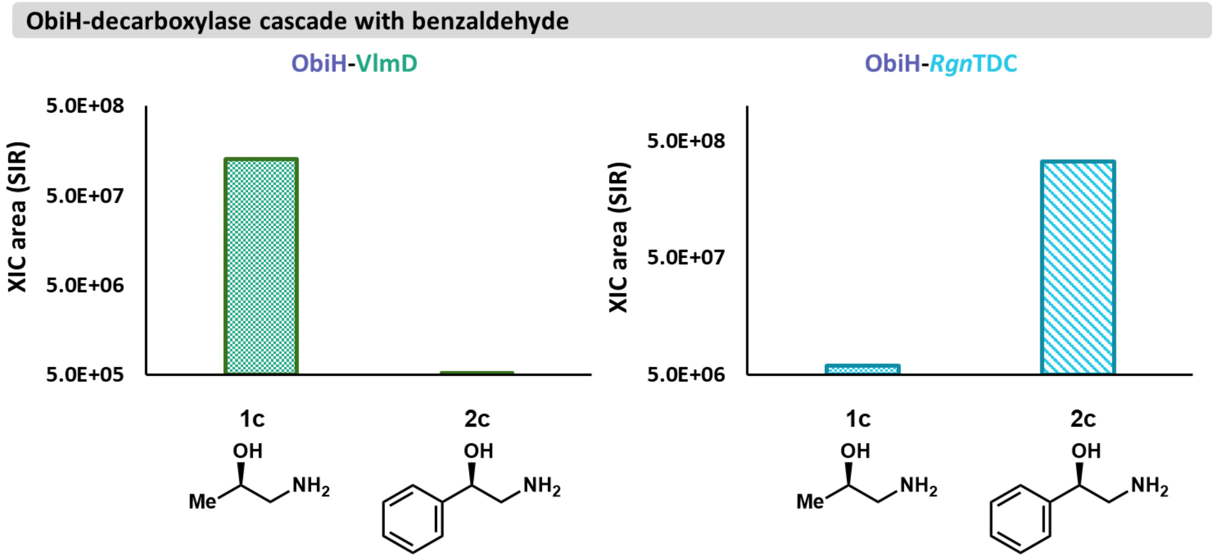

Figure S2. Products observed from cascade reactions with ObiH. 10 mM benzaldehyde, 50 mM Thr, 400 μM PLP, 50 mM KPi buffer pH = 8.0, and 10 μM VlmD/*Rgn*TDC (final volume = 100 μL). Reactions were allowed to proceed for 16 h at 37 °C. XIC = extracted ion count; (M+1)/z = 76 for **1c** and (M+1)/z = 138 for **2c**.

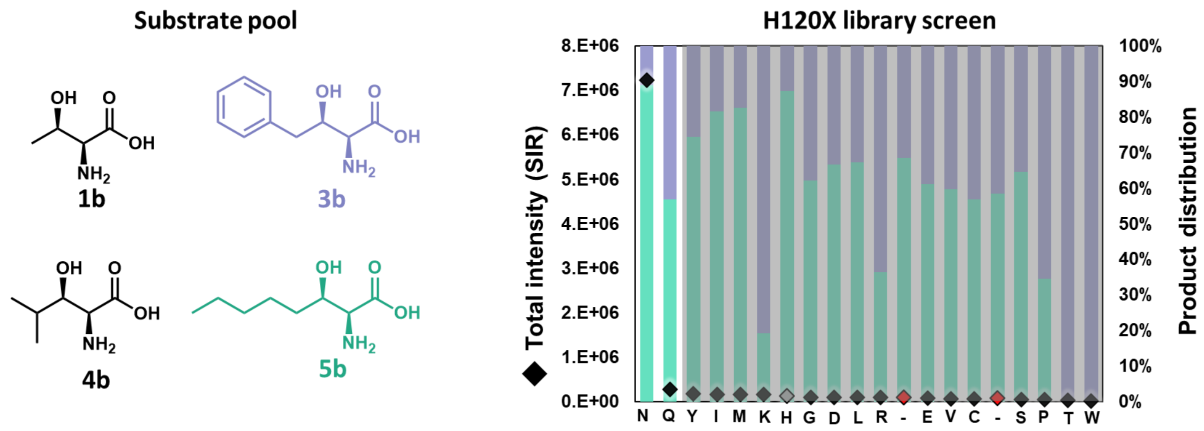

Figure S3. Screening results from the H120X site-saturation mutagenesis library. Colored bars represent relative amounts of each product formed, and diamonds represent mM total product produced, as determined by single-ion retention areas. The wild-type sequence is denoted by a grey diamond. Relative product amounts and mM total product were averaged from all wells with the given sequence. The greyed-out section of the graph indicates measurements that were indistinguishable from noise.

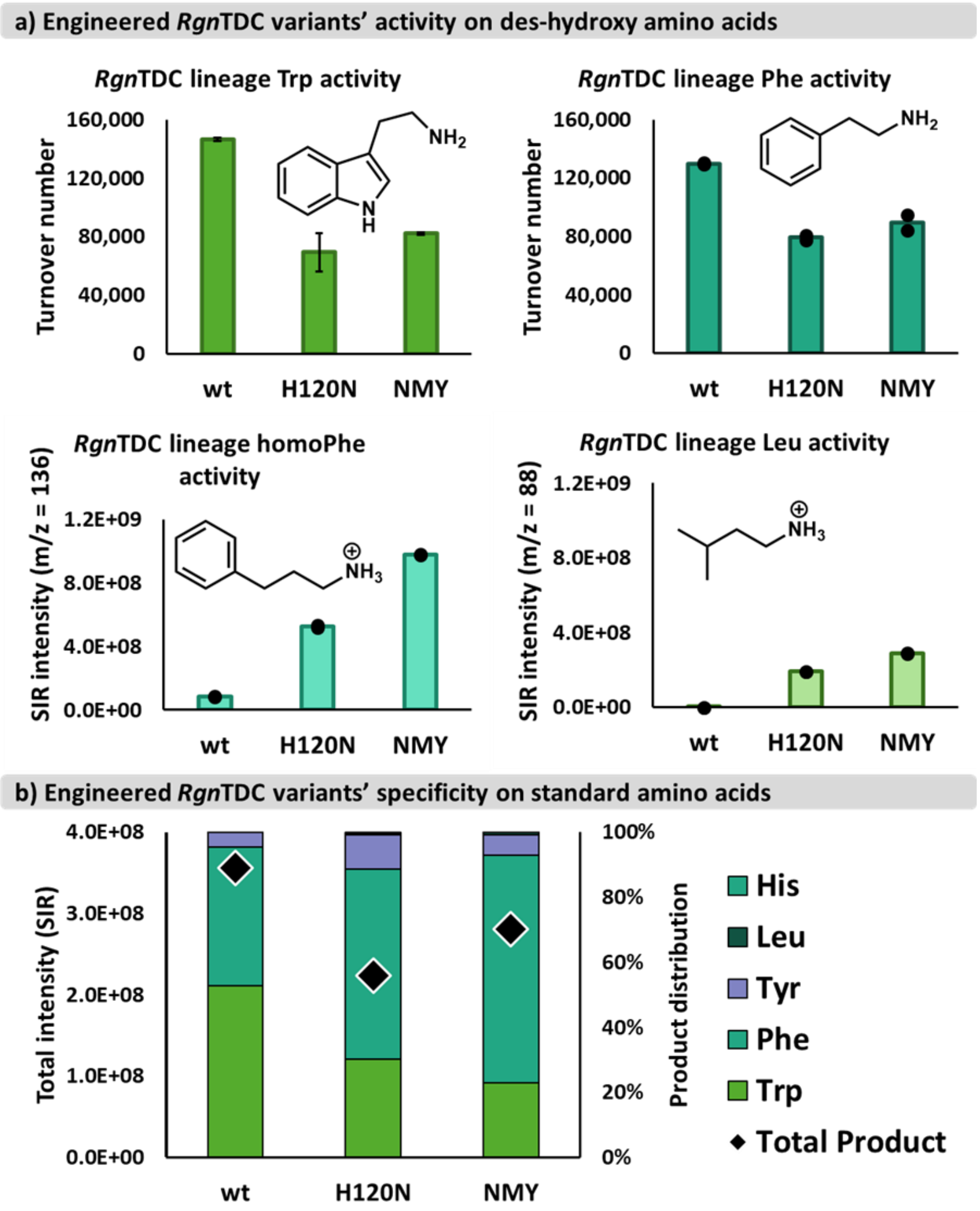

Figure S4. Activity of *Rgn*TDC variants with des-β-OH amino acids. a) Single-substrate activity comparisons. Reaction conditions: 10 mM amino acid, 400 μM PLP, 0.05 μM *Rgn*TDC (for Trp/Phe) or 10 μM *Rgn*TDC (for homoPhe/Leu), and 50 mM KPi buffer pH = 8.0 (final volume = 100 μL). Reactions were allowed to proceed for 16 h at 37 °C. Reactions for Trp were conducted in triplicate and all other reactions were conducted in duplicate. b) Analysis of *Rgn*TDC variant specificities for standard amino acids. Reaction conditions: 2 mM each substrate (His, Leu, Tyr, Phe, Trp), 400 μM PLP, 1 μM *Rgn*TDC variant, and 50 mM KPi buffer pH = 8.0 (final volume = 100 μL). Reactions were conducted in duplicate for 10 min at 37 °C. Average product single ion retention (SIR) areas were integrated to quantitate relative amounts of product formed.

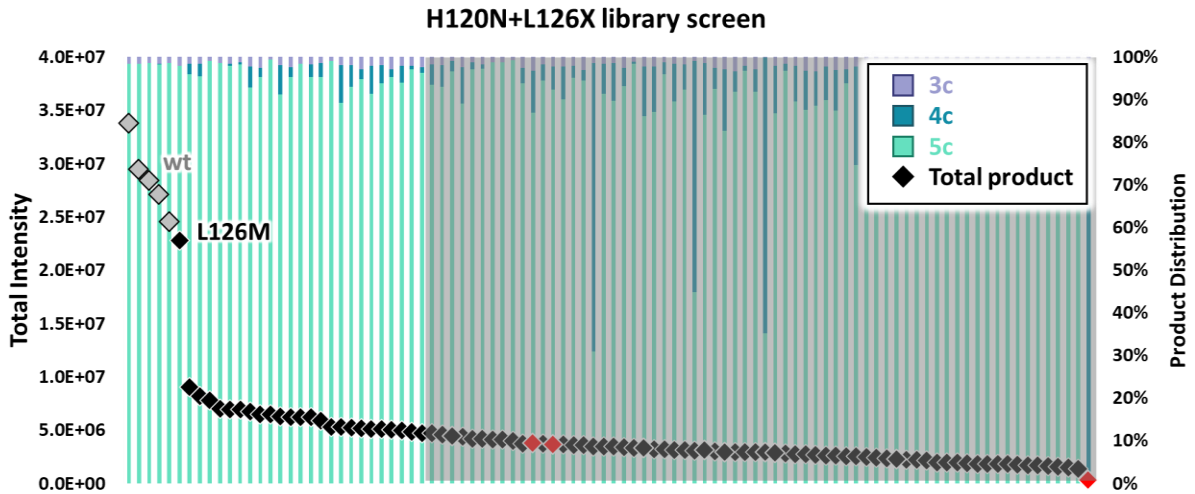

Figure S5. Screening results from the H120N + L126X site-saturation mutagenesis library. Colored bars represent relative amounts of each product formed, and diamonds represent mM total product produced, as determined by single-ion retention areas. The wild-type sequence is denoted by a grey diamond, and sequenced wells are labeled. Relative product amounts and mM total product were averaged from all wells with the given sequence. The greyed-out section of the graph indicates measurements that were indistinguishable from noise.

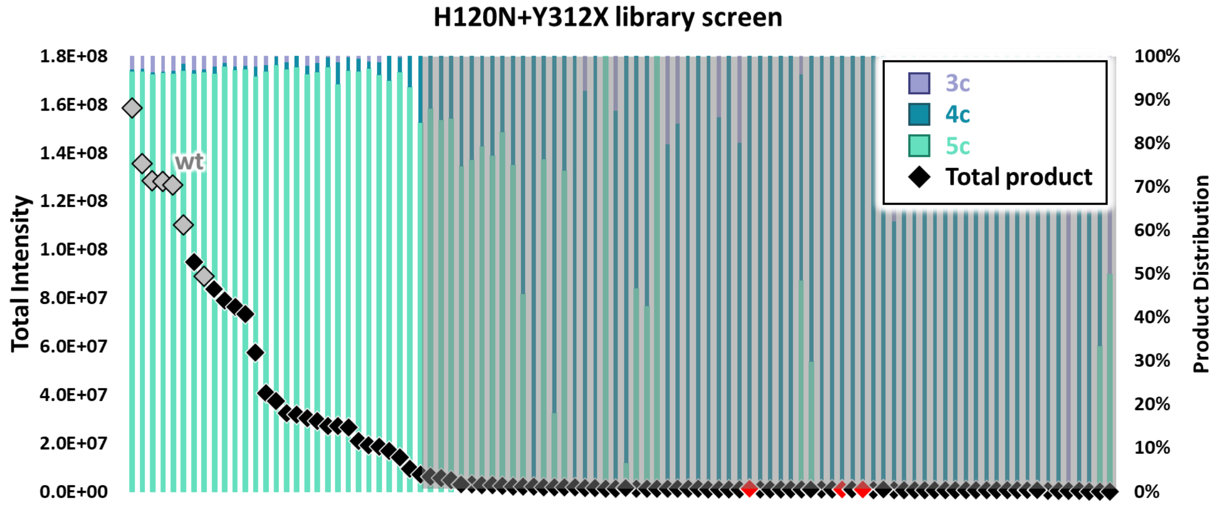

Figure S6. Screening results from the H120N + Y312X site-saturation mutagenesis library. Colored bars represent relative amounts of each product formed, and diamonds represent mM total product produced, as determined by single-ion retention areas. The wild-type sequence is denoted by a grey diamond, and sequenced wells are labeled. Relative product amounts and mM total product were averaged from all wells with the given sequence. The greyed-out section of the graph indicates measurements that were indistinguishable from noise.

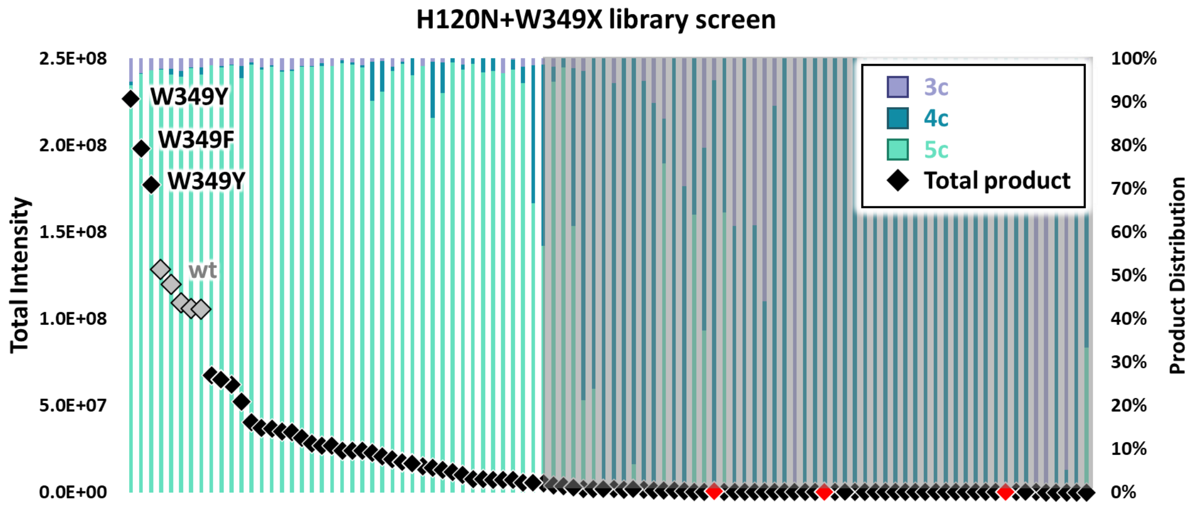

Figure S7. Screening results from the H120N + W349X site-saturation mutagenesis library. Colored bars represent relative amounts of each product formed, and diamonds represent mM total product produced, as determined by single-ion retention areas. The wild-type sequence is denoted by a grey diamond, and sequenced wells are labeled. Relative product amounts and mM total product were averaged from all wells with the given sequence. The greyed-out section of the graph indicates measurements that were indistinguishable from noise.

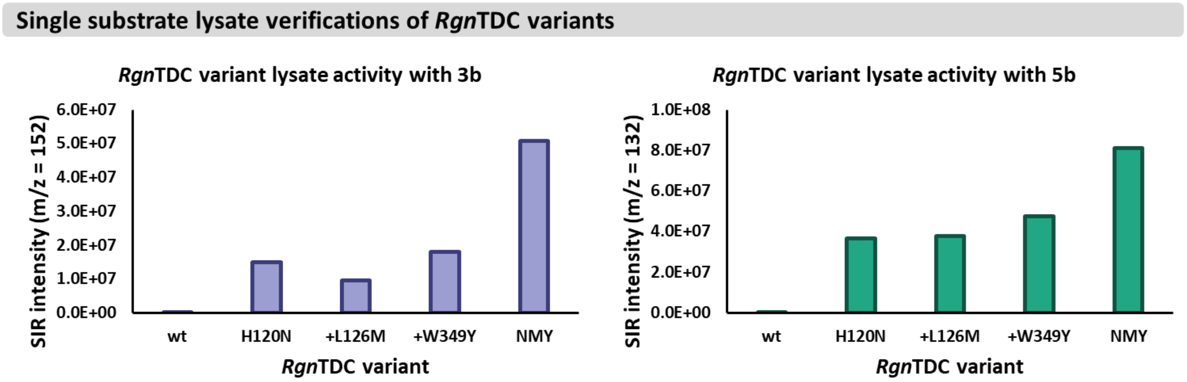

Figure S8. *Rgn*TDC variants lysate activity verifications on single substrates. Reaction conditions: 90 µL lysate supernatant + 10 µL 100 mM β-OH amino acid (**3b**, **4b**, or **5b**) at 37 °C for 4 h. Single ion retention (SIR) peak areas were used to determine product abundance. No product was detected for reactions with **4b**.

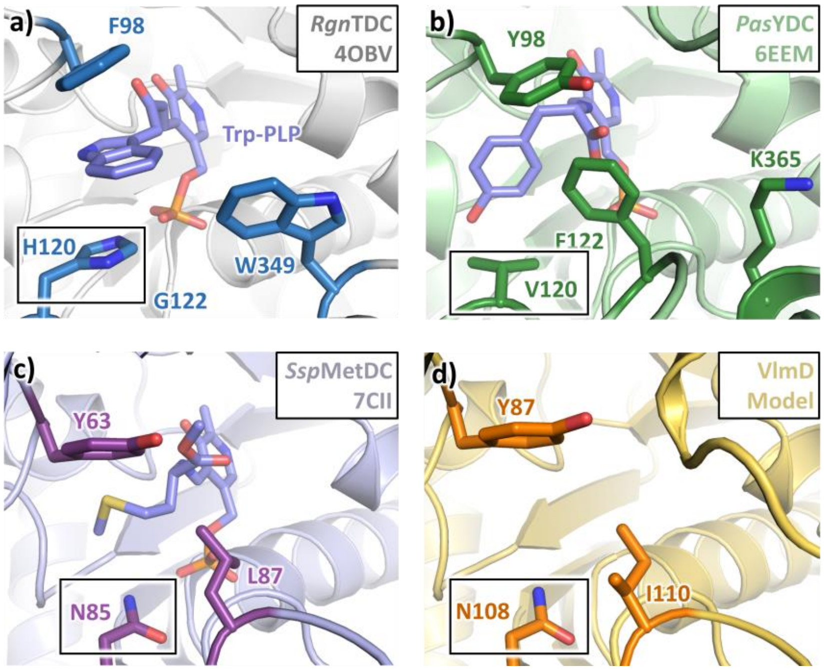

Figure S9. Active-site comparisons between various amino acid decarboxylases. PDB ID's are denoted for each structure (*Rgn*TDC = 4OBV, *Pas*YDC = 6EEM, *Ssp*MetDC = 7CII). The residues in the black box are analogous to the H120 residue of *Rgn*TDC. The structure of VlmD was generated using SwissModel with 7CII serving as the parent structure.

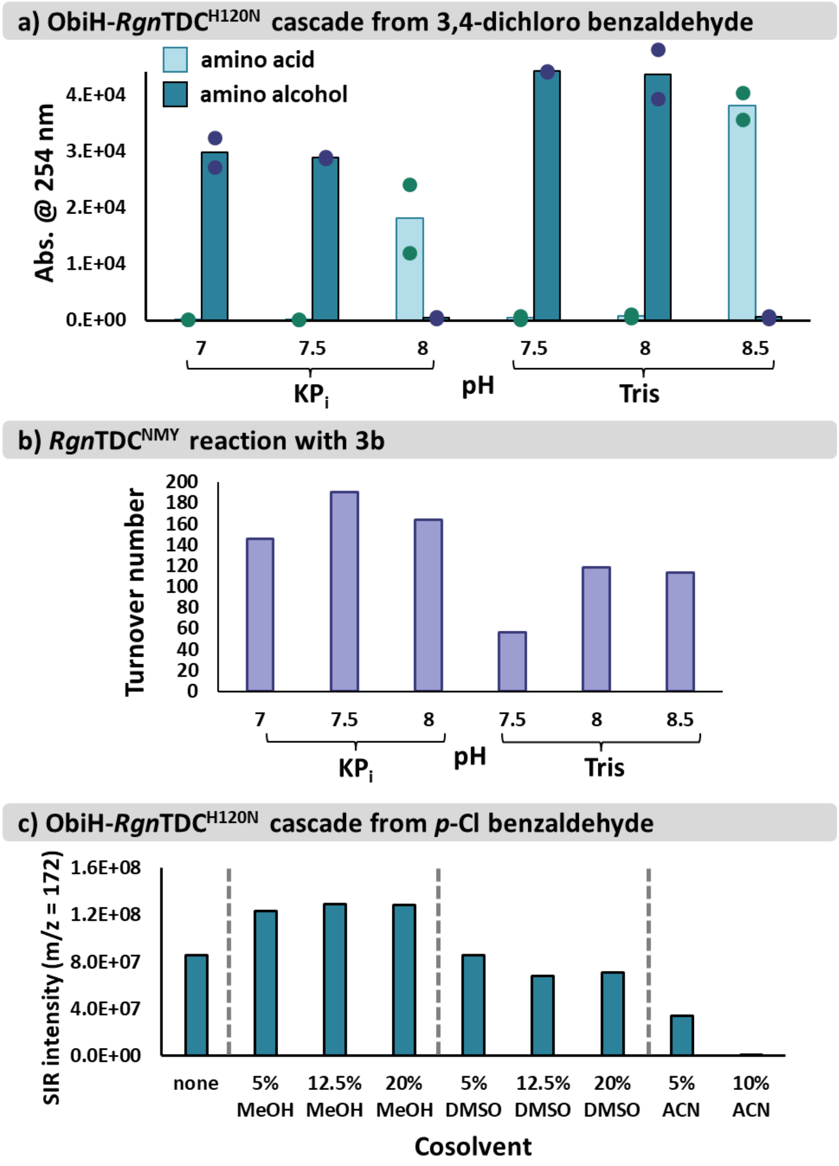

Figure S10. Determination of preferred buffer and pH system for ObiH – *Rgn*TDC cascade. a) Buffer screen for ObiH – RgnTDC^{H120N}**.** Reaction conditions: 25 mM 3,4-dichlorobenzaldehyde, 75 mM Thr, 400 μM PLP, 10 μM ObiH, 25 μM *Rgn*TDC^{H120N}, and 50 mM Tris/KPi buffer with 5% MeOH (final volume = 250 μL). Buffer pH's ranged from pH = 7.0 to pH = 8.5. Reactions were conducted for 16 h at 37 °C at 180 rpm. Product abundance was determined by absorbance at 254 nm. b) Buffer screen for *Rgn*TDC^{NMY}. Reaction conditions: 10 mM **3b**, 400 μM PLP, 10 μM *Rgn*TDC^{NMY}, and 50 mM Tris/KPi buffer (final volume = 100 μL). Buffer pH's ranged from pH = 7.0 to pH = 8.5. Reactions were conducted for 16 h at 37 °C at 180 rpm. Turnover numbers were determined by relative peak areas at 254 nm. c) Cosolvent screen for ObiH – *Rgn*TDC^{H120N} cascade. Reaction conditions: 25 mM *p*-chlorobenzaldehyde, 80 mM Thr, 200 μM PLP, 50 mM Tris-HCl buffer pH = 8.0, 32 μM ObiH, 12 μM *Rgn*TDC^{H120N} (final volume = 250 μL).

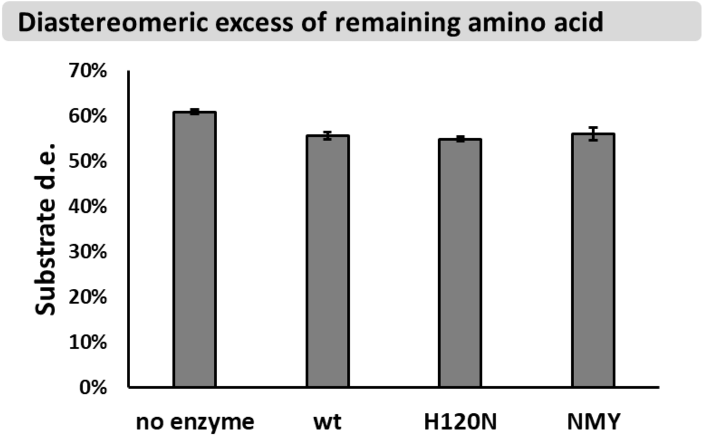

Figure S11: Analysis of *Rgn*TDC variant diastereoselectivity. Reaction conditions: 10 mM *p*-Br, β-OH Phe (**6b**) **(**80:20 d.r.), 400 μM PLP, 10 μM *Rgn*TDC variant, and 50 mM KPi buffer pH = 8.0 (final volume = 100 μL). Substrate d.e. was measured after incubation with *Rgn*TDC variants for 15 min at 37 °C corresponding with ~20% substrate conversion.

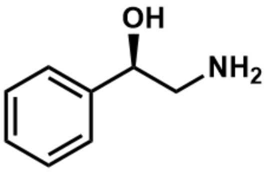

2c
A - 51%, 96:4 e.r.

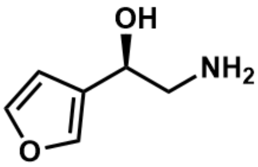

13c
A - 0%
amino acid yield: 0%

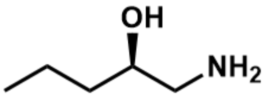

17c
A - 0%
amino acid yield: 12%

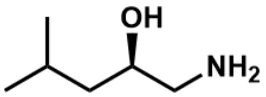

18c
A - 0%
amino acid yield: 77%

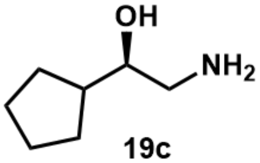

19c
A - 0%
amino acid yield: 47%

Figure S12. Additional substrates tested in ObiH – *Rgn*TDC^{NMY} cascade.

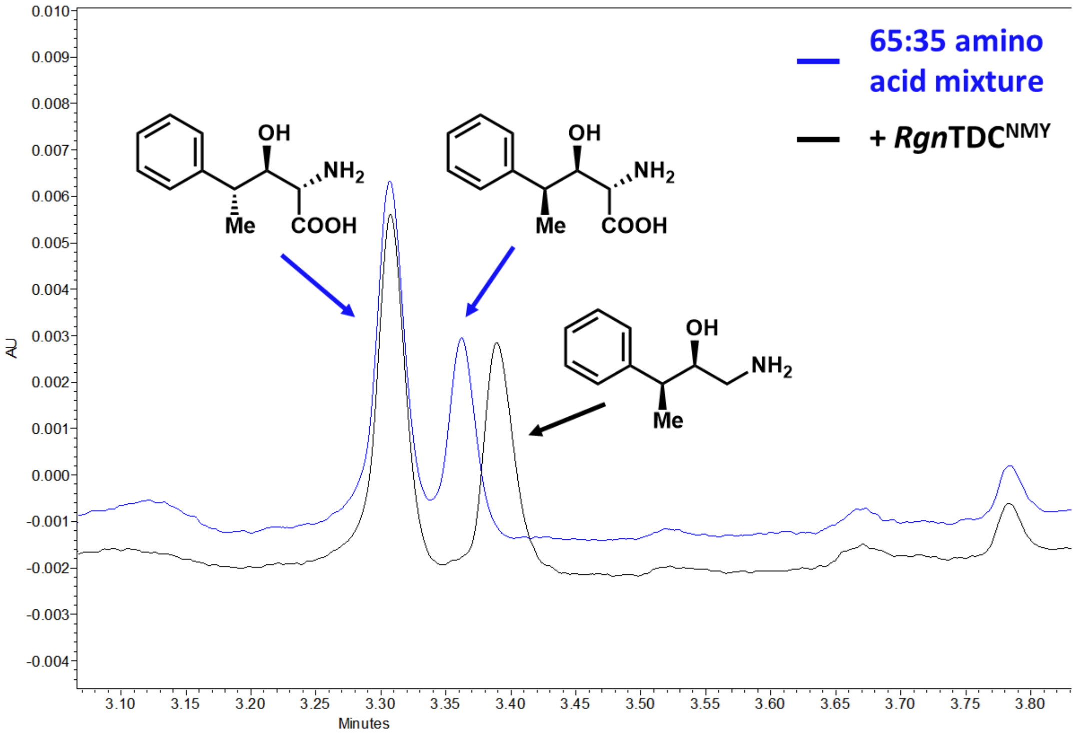

Figure S13. Determination of *Rgn*TDC γ-Me diastereoselectivity. 10 mM **21b** (65:35 *anti*:*syn* for the γ-Me and β-OH groups; prepared as previously described[2]), 400 μM PLP, 0 or 10 μM *Rgn*TDC^{NMY}, 50 mM KPi buffer pH = 8.0 (final volume = 100 μL). Reactions were conducted in duplicate for 16 h at 37 °C.

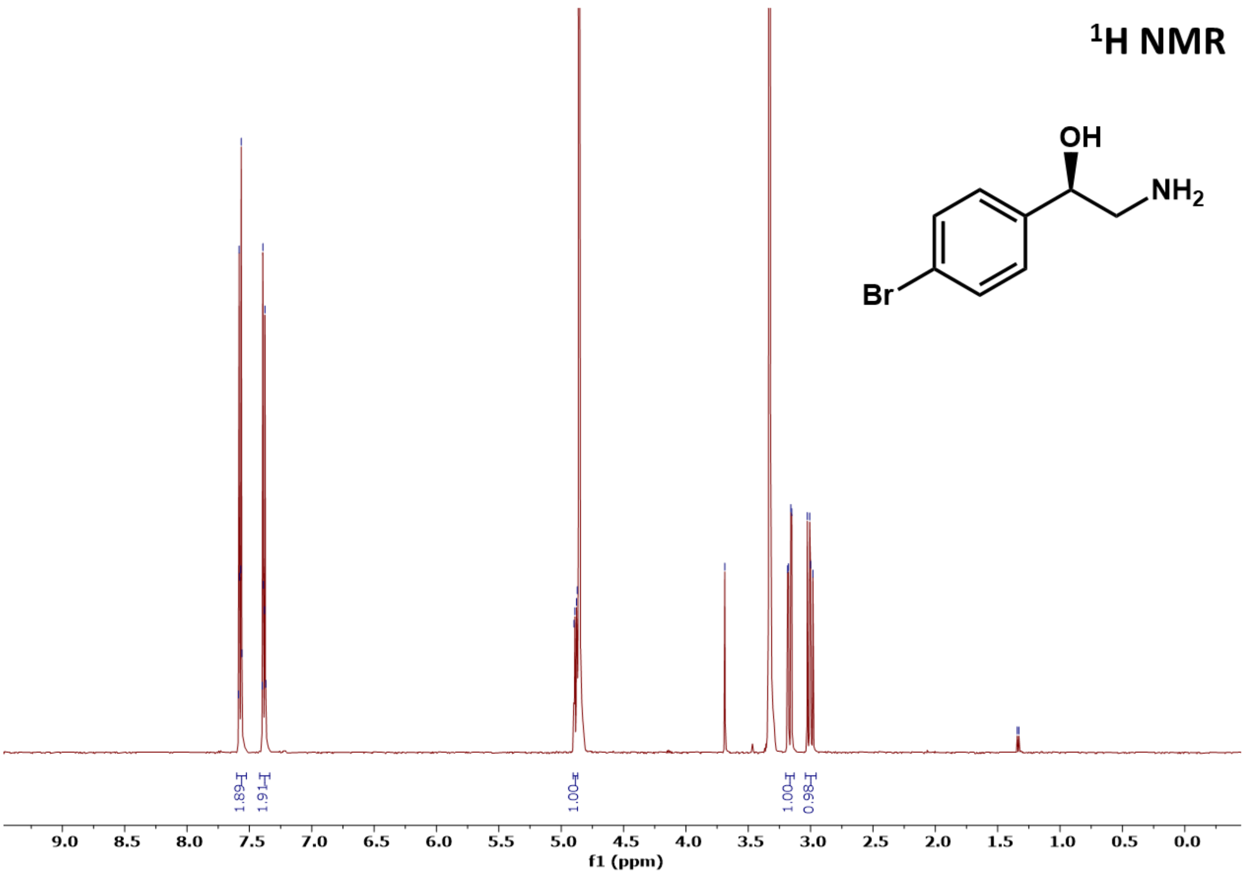

Figure S14. **^{1}H NMR spectrum of (*R*)-2-amino-1-(4-bromophenyl)ethan-1-ol (6c).**

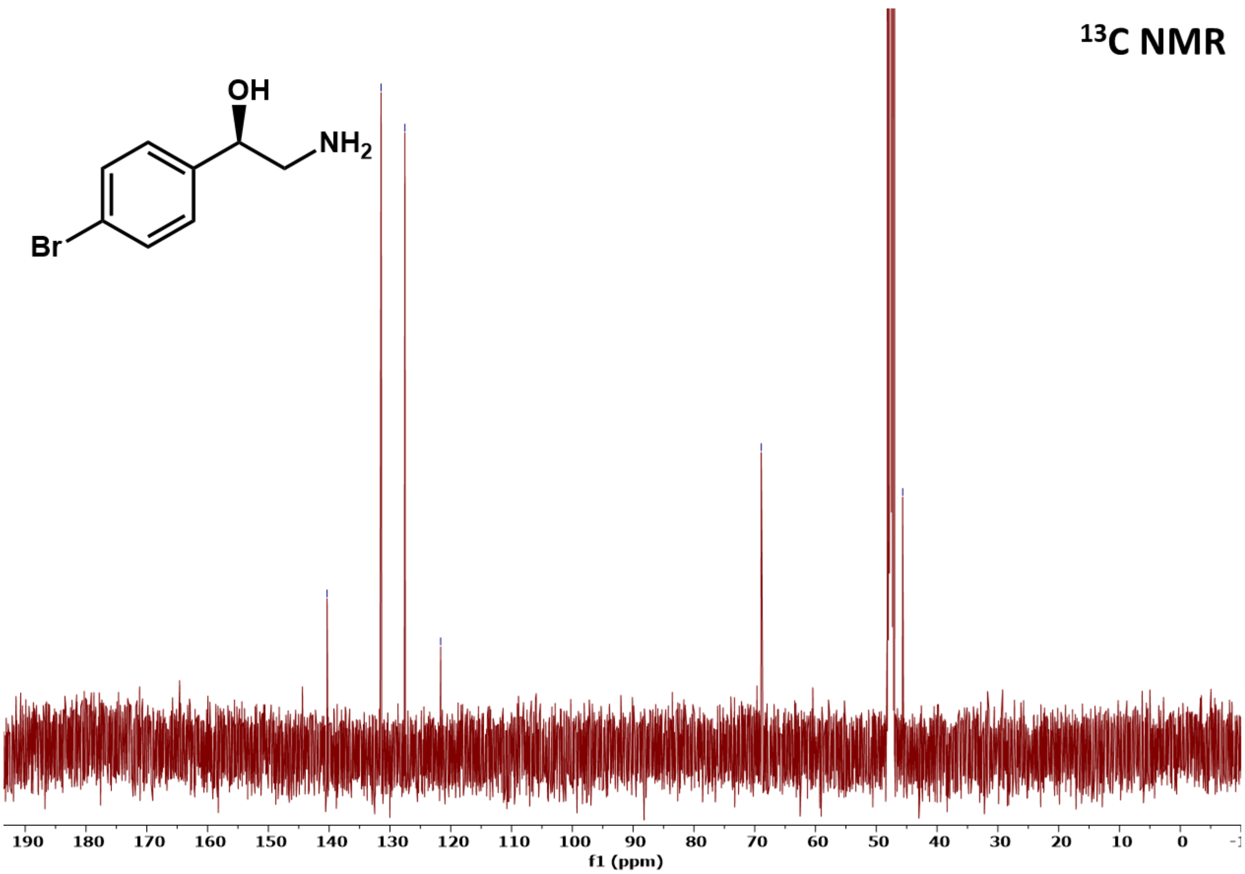

Figure S15. ^{13}**C NMR spectrum of (*R*)-2-amino-1-(4-bromophenyl)ethan-1-ol (6c).**

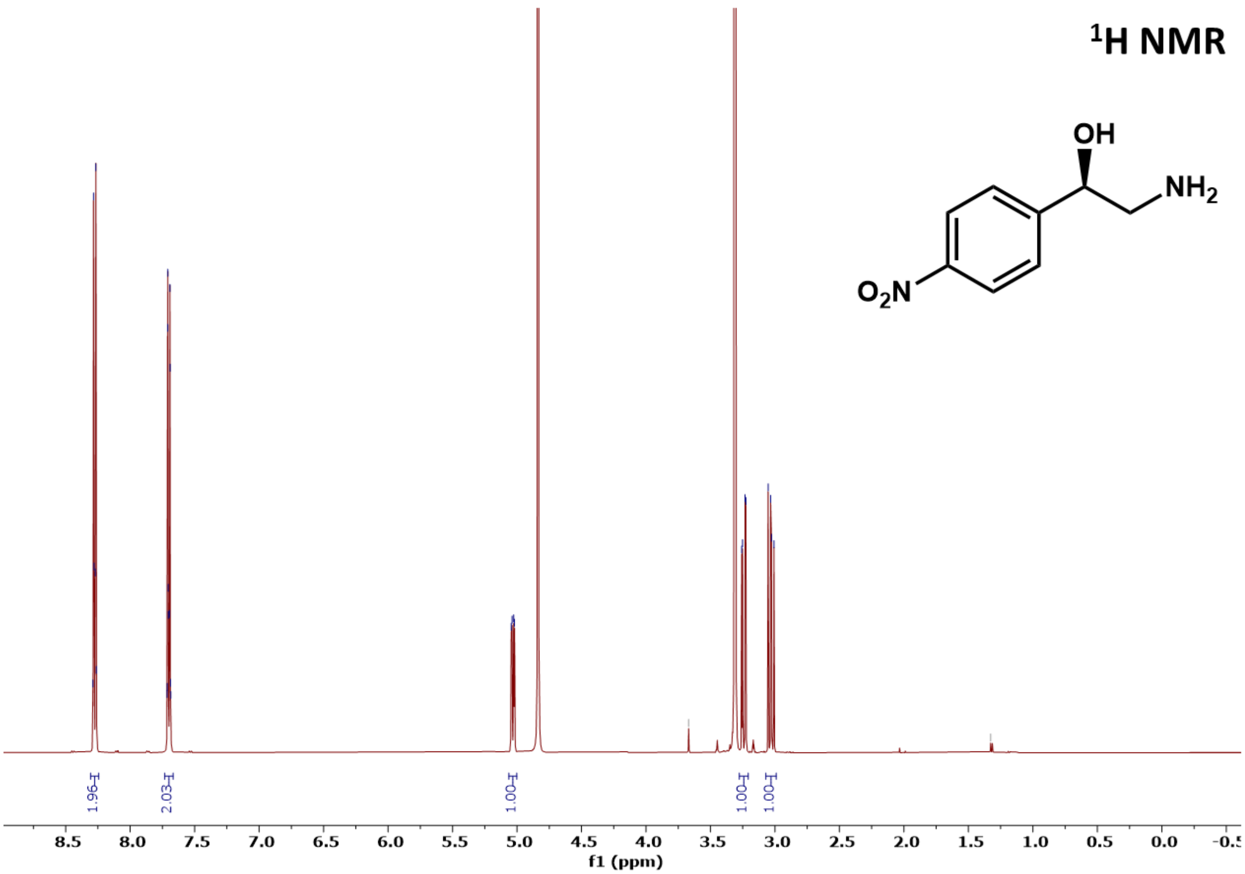

Figure S16. ^{1}**H NMR spectrum of (*R*)-2-amino-1-(4-nitrophenyl)ethan-1-ol (7c).**

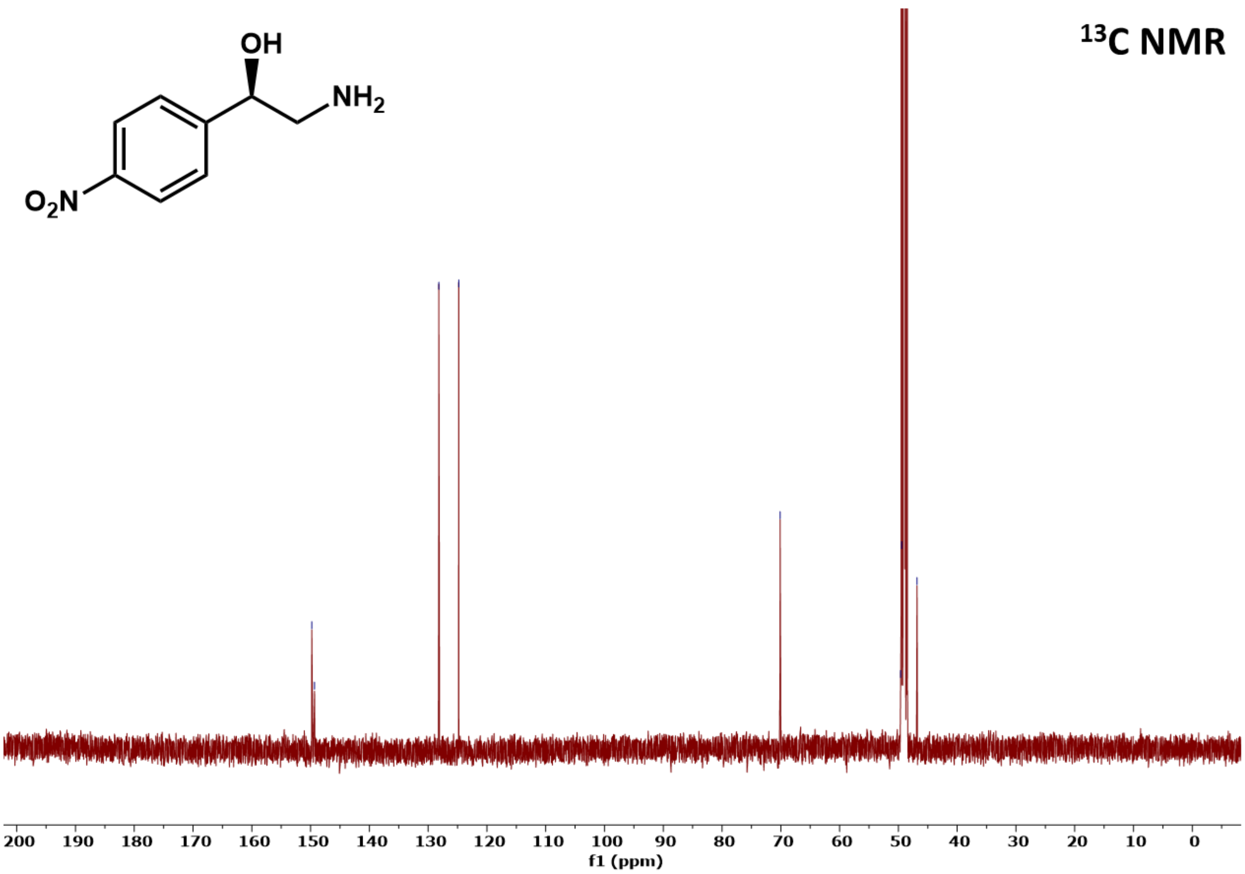

Figure S17. **^{13}C NMR spectrum of (*R*)-2-amino-1-(4-nitrophenyl)ethan-1-ol (7c).**

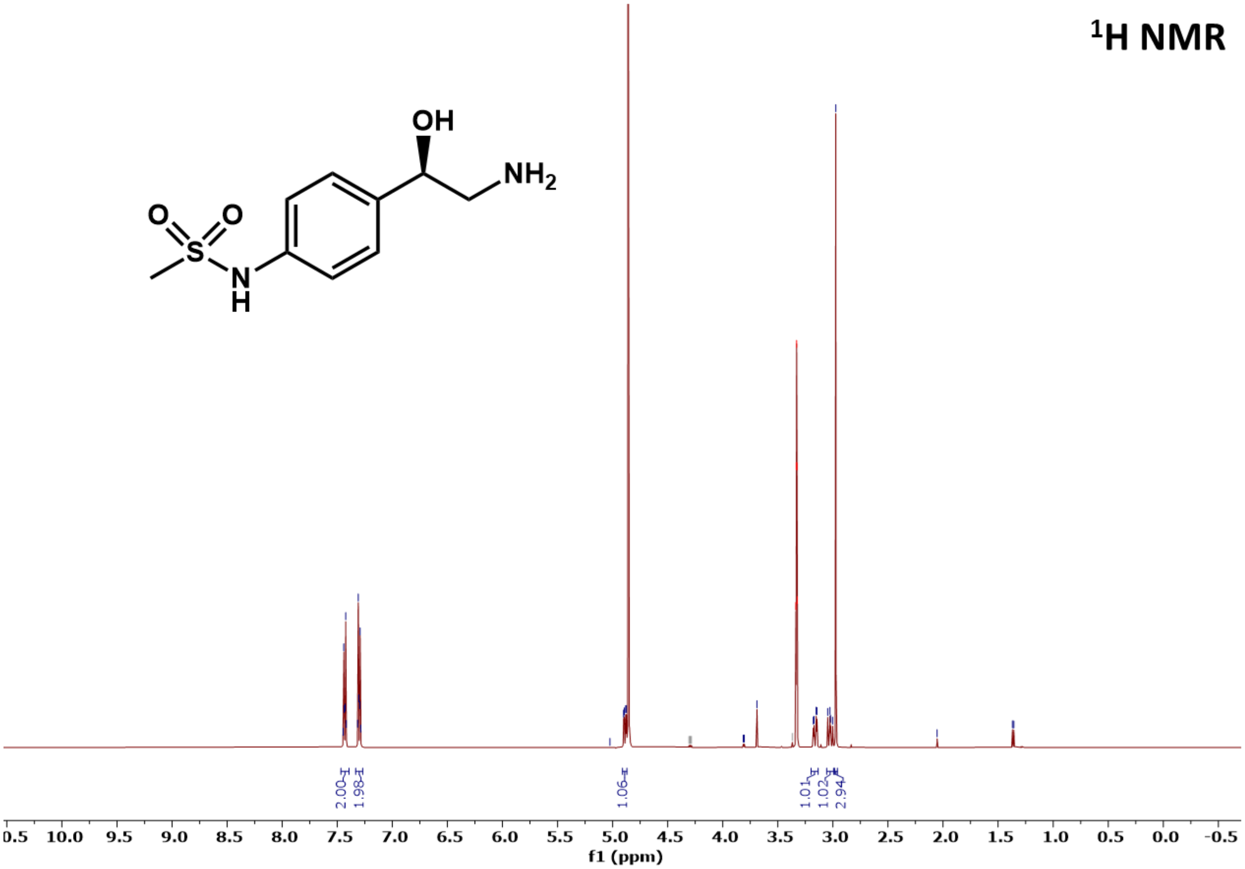

Figure S18. **^{1}H NMR spectrum of (*R*)-N-(4-(2-amino-1-hydroxyethyl)phenyl)methanesulfonamide (8c).**

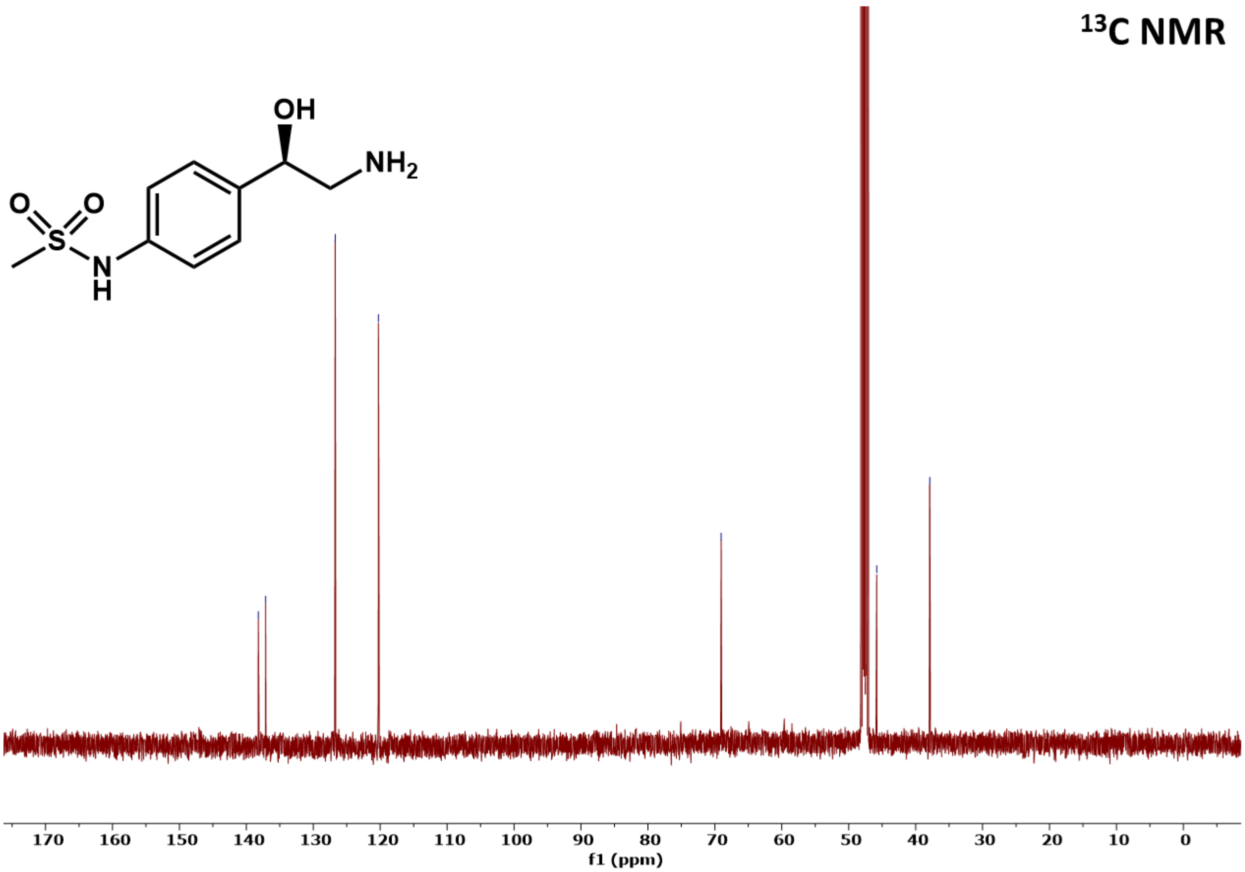

Figure S19. ^{13}**C NMR spectrum of (*R*)-N-(4-(2-amino-1-hydroxyethyl)phenyl)methanesulfonamide (8c).**

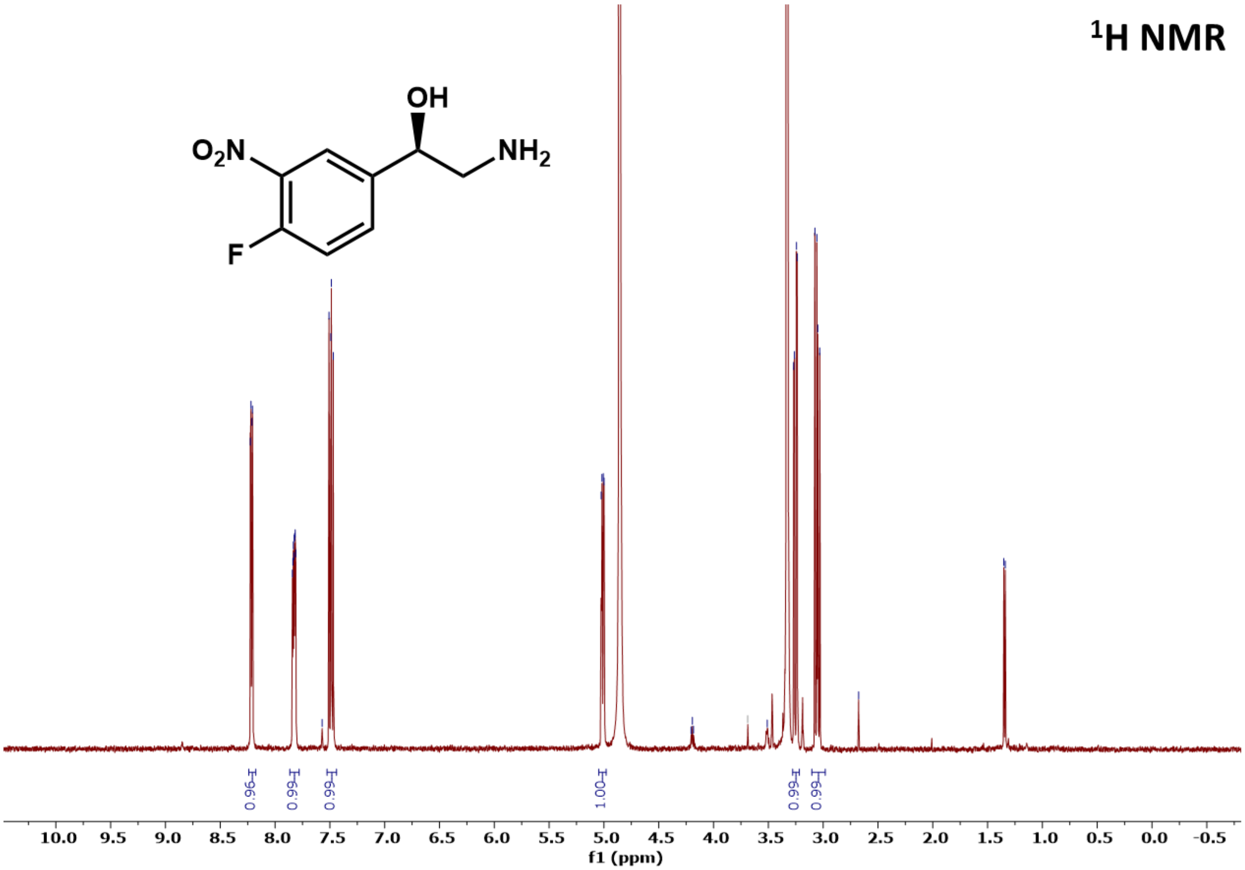

Figure S20. ^{1}H **NMR spectrum of (*R*)-2-amino-1-(4-fluoro-3-nitrophenyl)ethan-1-ol (9c).**

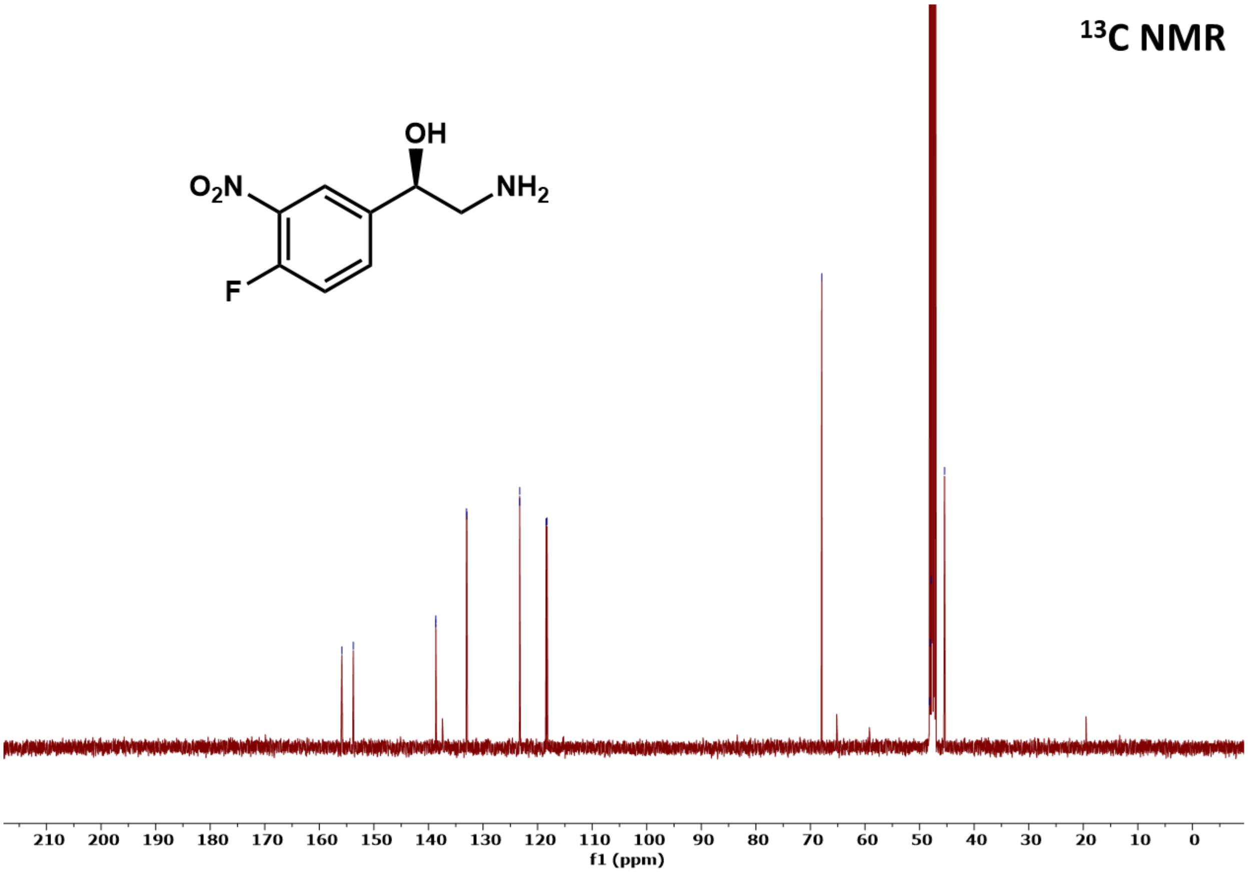

Figure S21. ^{13}**C NMR spectrum of (*R*)-2-amino-1-(4-fluoro-3-nitrophenyl)ethan-1-ol (9c).**

^{19}F NMR

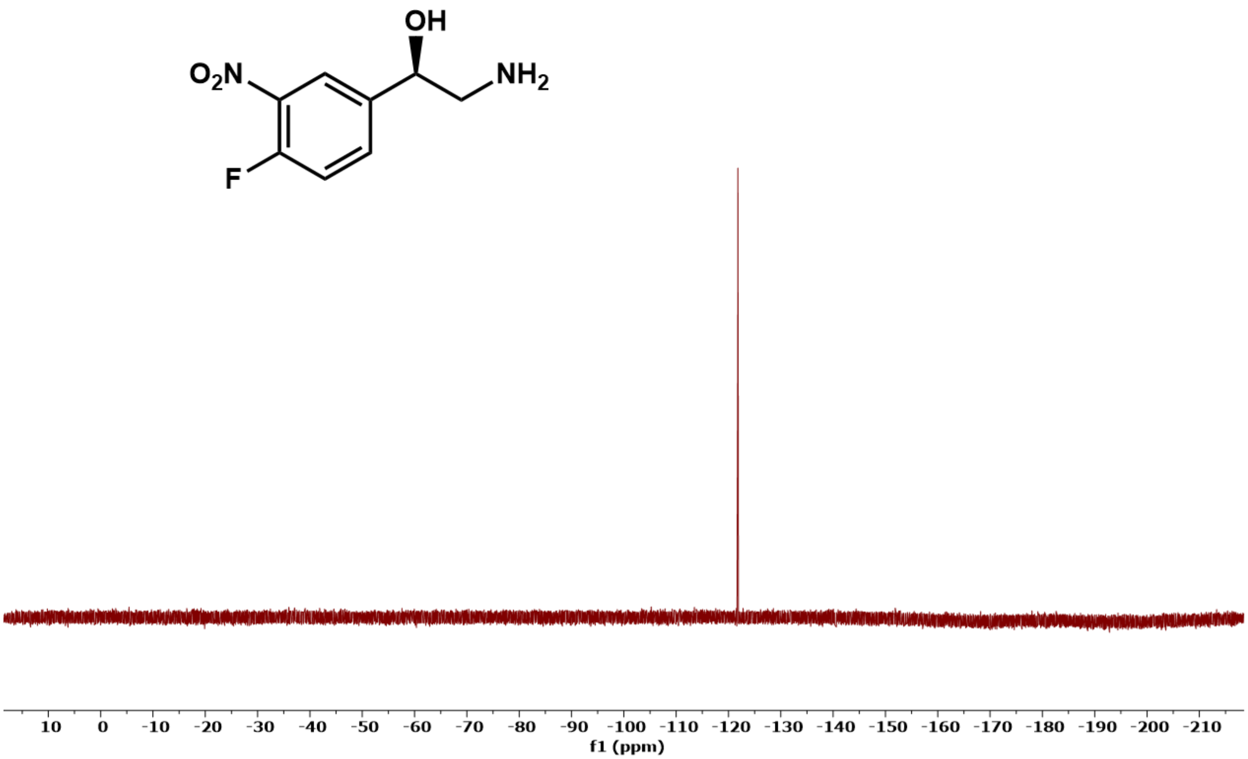

Figure S22. ^{19}F NMR spectrum of (*R*)-2-amino-1-(4-fluoro-3-nitrophenyl)ethan-1-ol (9c).

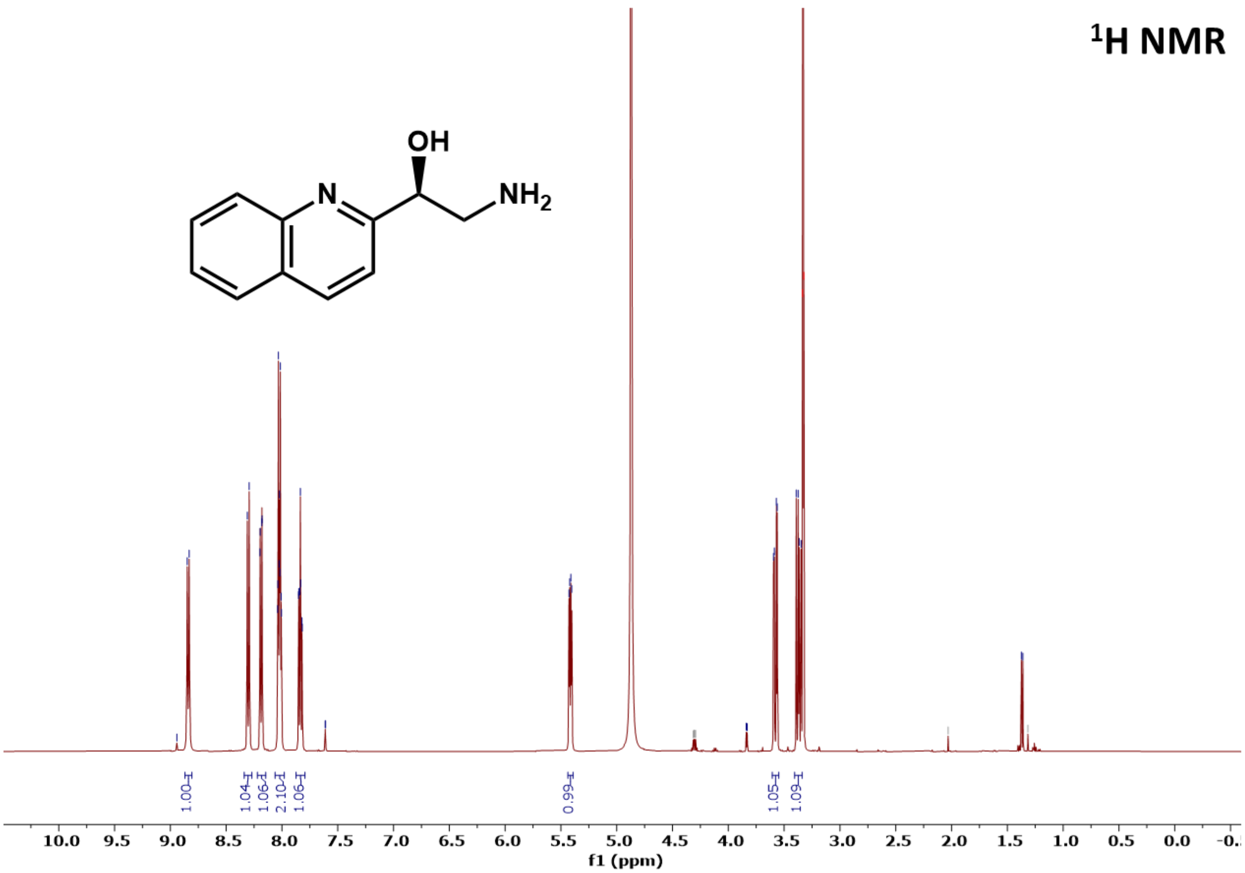

Figure S23. **^{1}H NMR spectrum of (*R*)-2-amino-1-(quinolin-3-yl)ethan-1-ol (10c).**

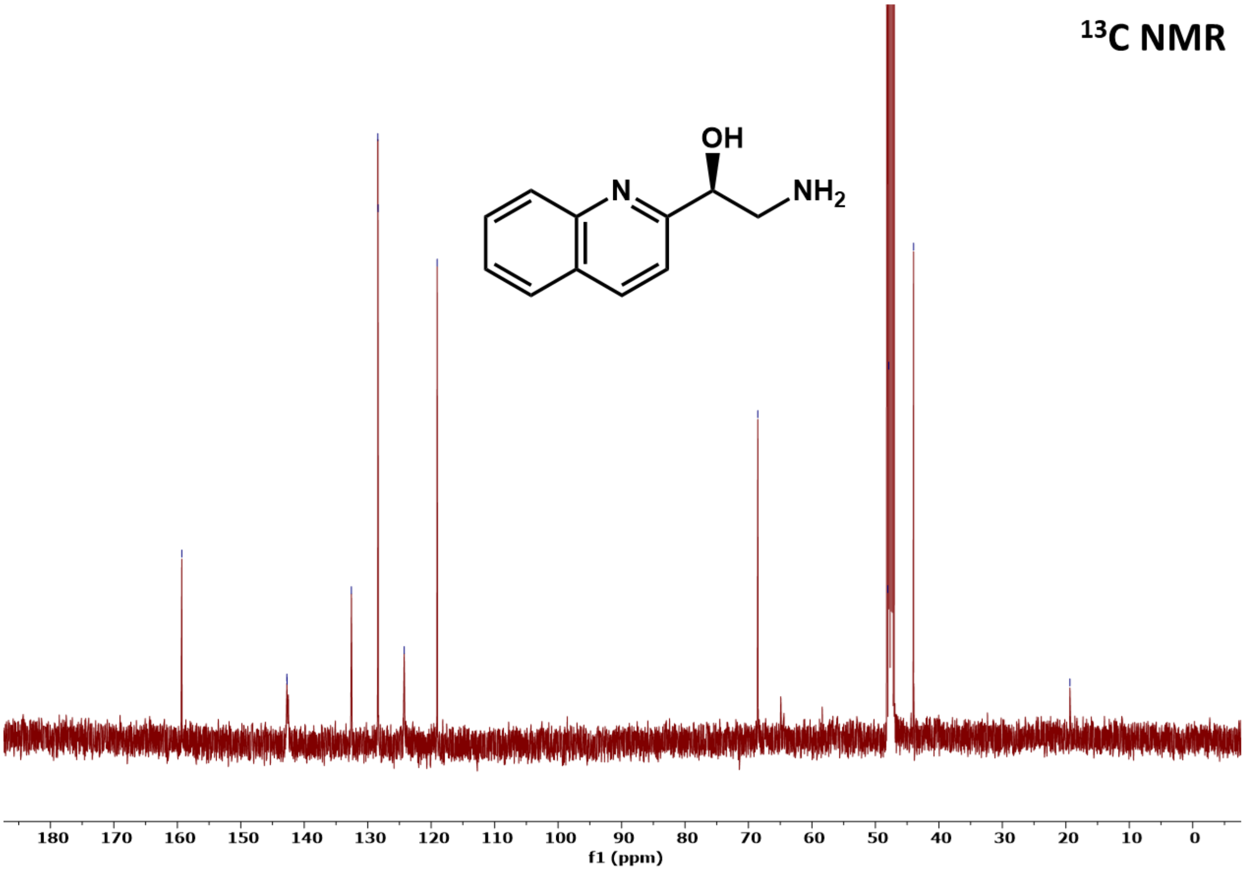

Figure S24. **^{13}C NMR spectrum of (*R*)-2-amino-1-(quinolin-3-yl)ethan-1-ol (10c).**

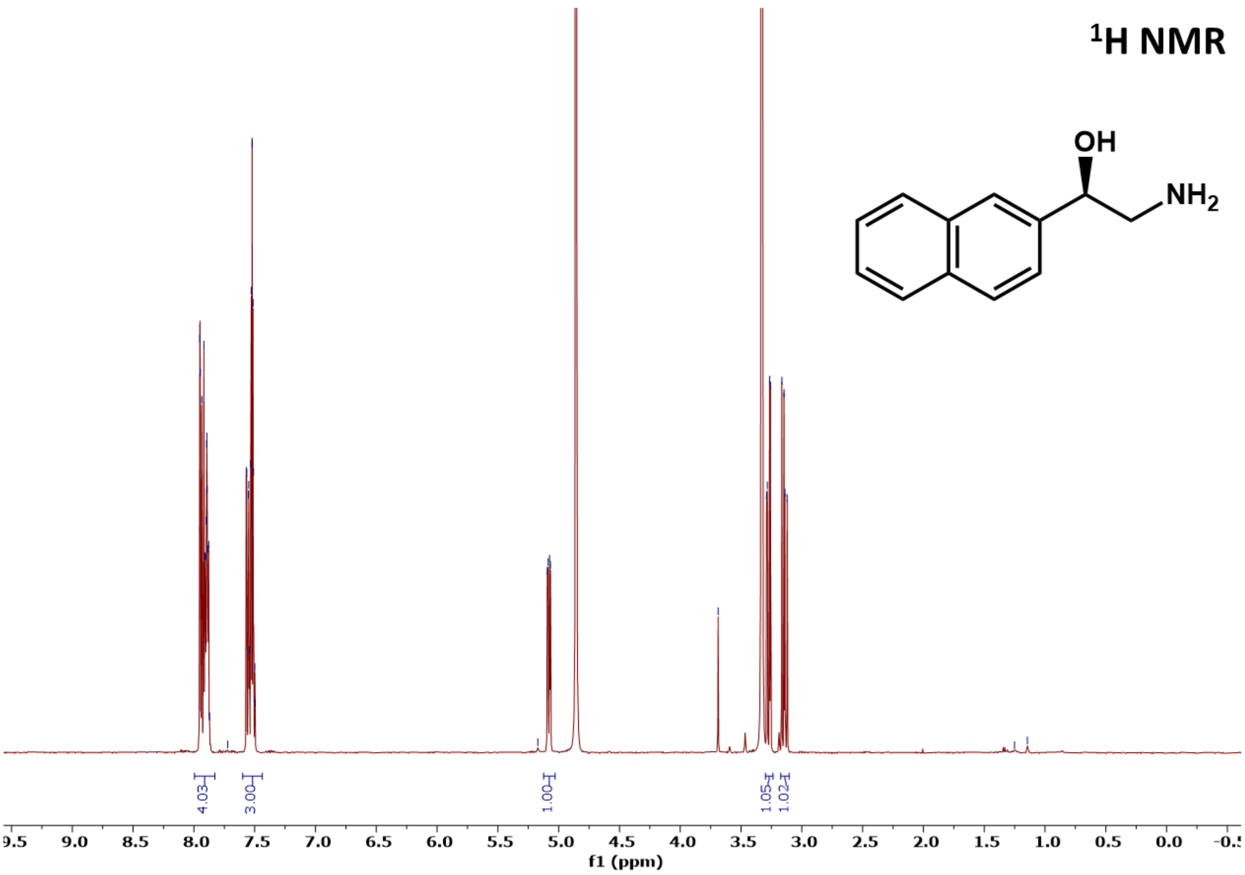

Figure S25. **^{1}H NMR spectrum of (*R*)-2-amino-1-(naphthalen-2-yl)ethan-1-ol (11c).**

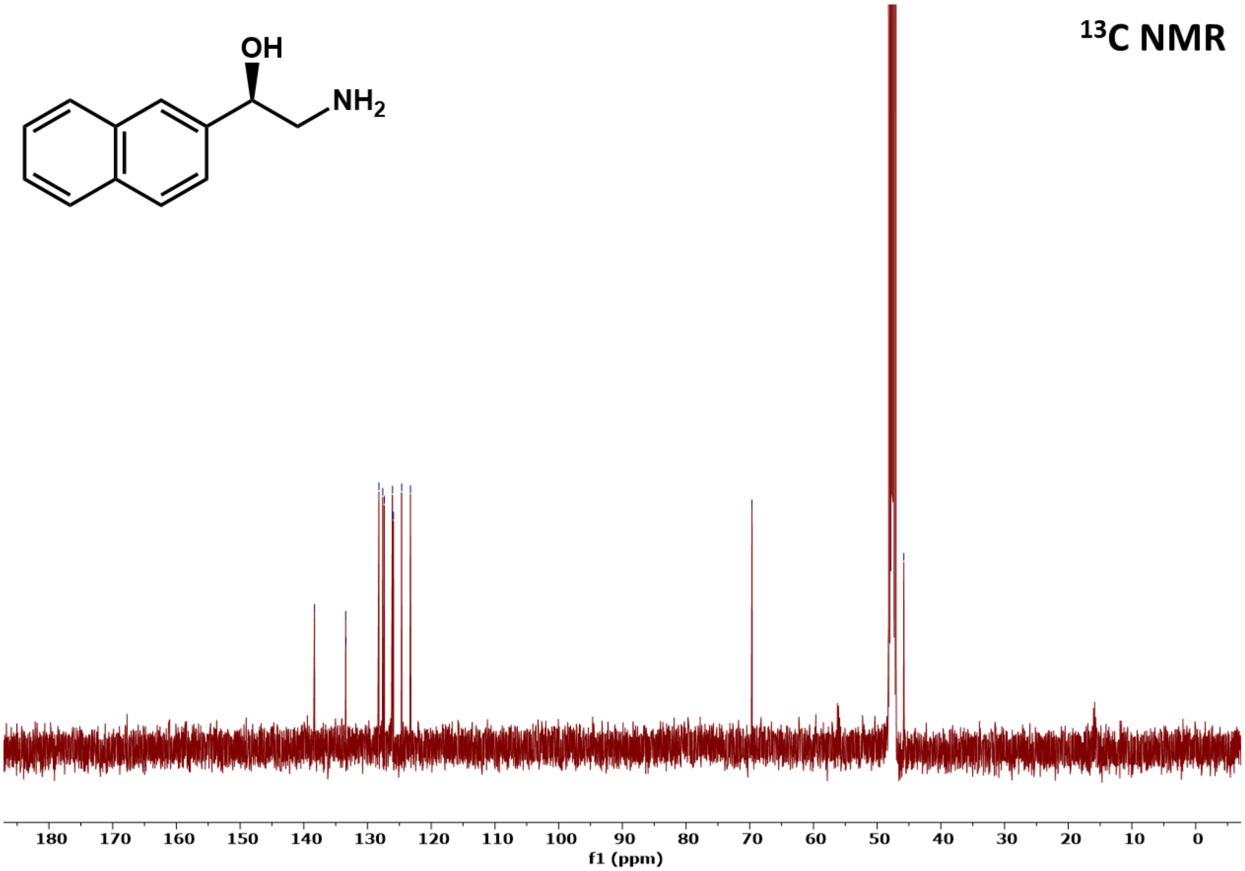

Figure S26. ^{13}**C NMR spectrum of (*R*)-2-amino-1-(naphthalen-2-yl)ethan-1-ol (11c).**

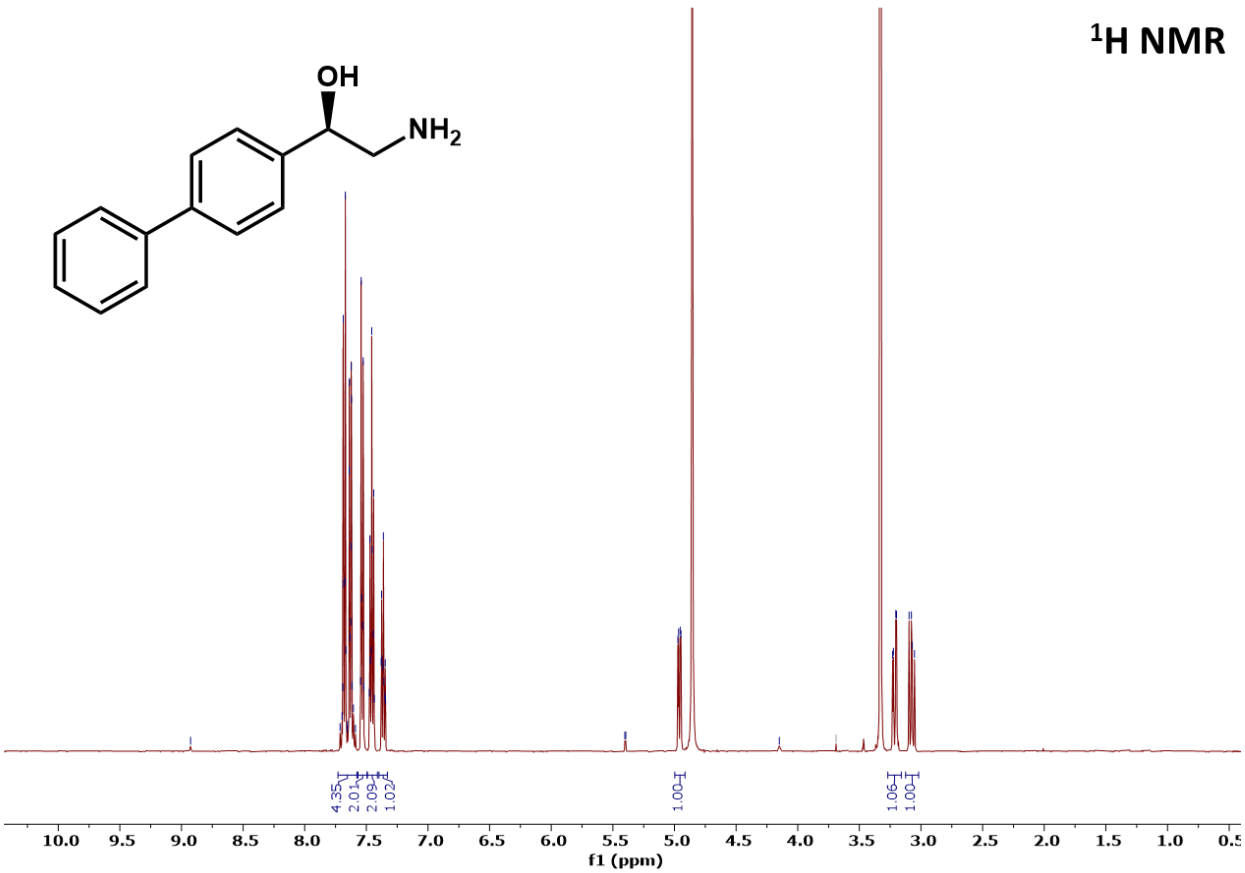

Figure S27. **^{1}H NMR spectrum of (*R*)-1-([1,1'-biphenyl]-4-yl)-2-aminoethan-1-ol (12c).**

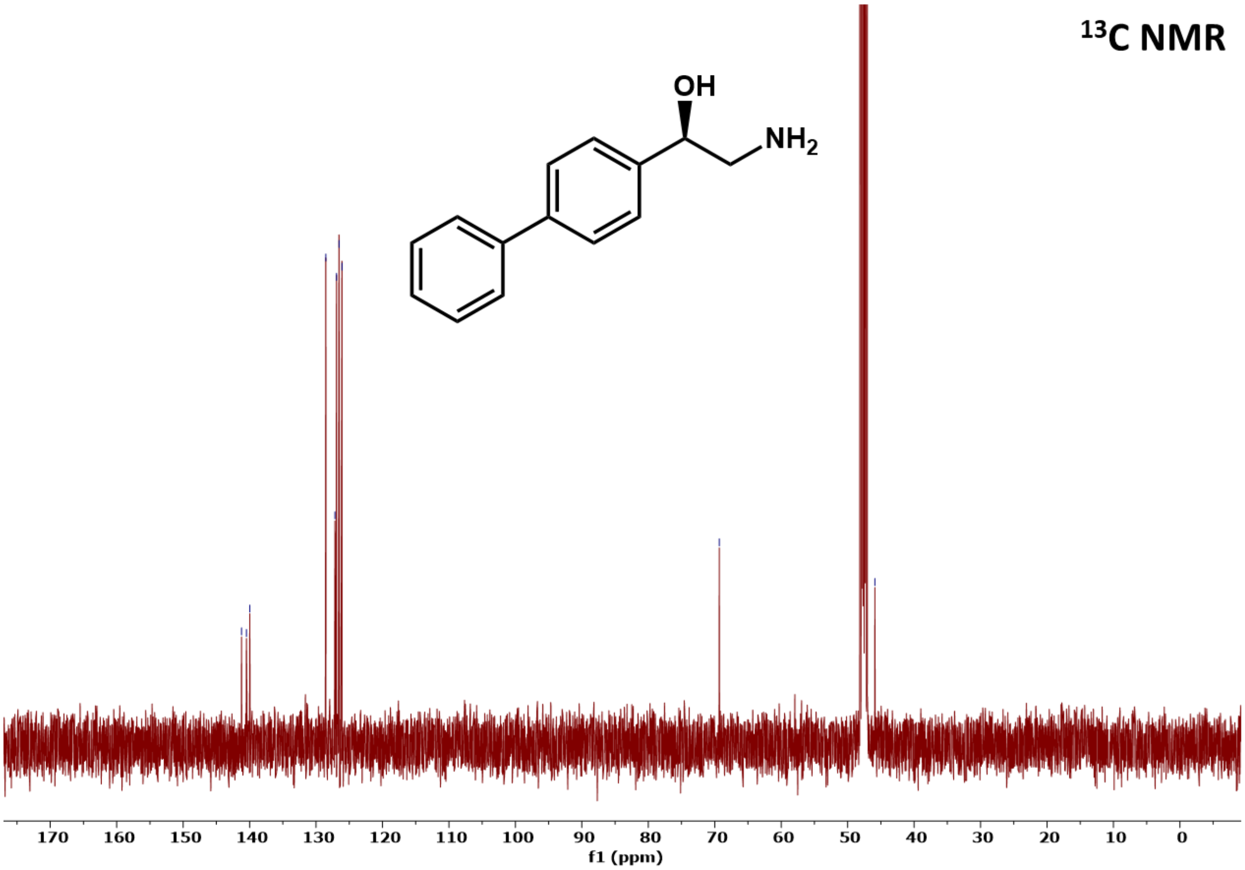

Figure S28. **^{13}C NMR spectrum of (*R*)-1-([1,1'-biphenyl]-4-yl)-2-aminoethan-1-ol (12c).**

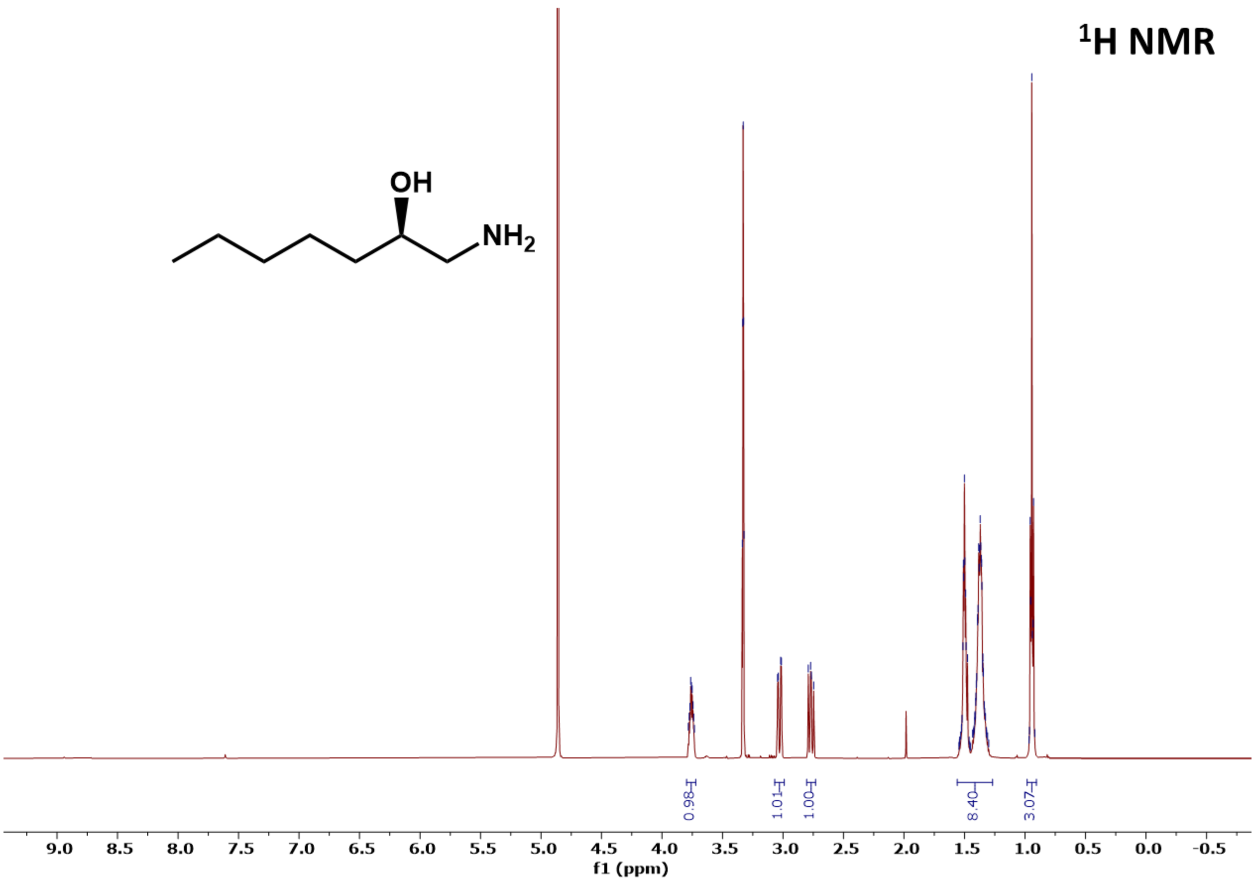

Figure S29. ^{1}**H NMR spectrum of (*R*)-1-aminoheptan-2-ol (5c).**

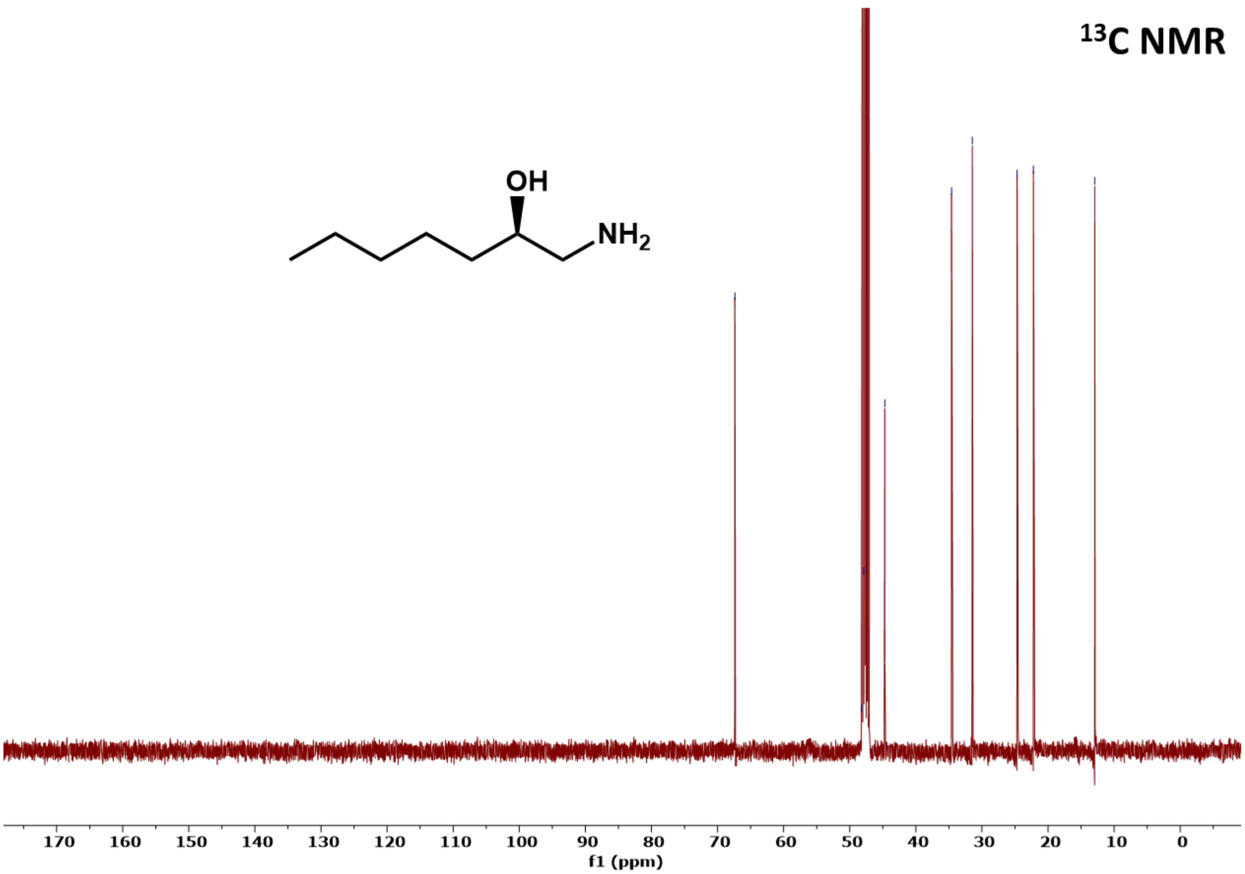

Figure S30. ^{13}C NMR spectrum of (*R*)-1-aminoheptan-2-ol (5c).

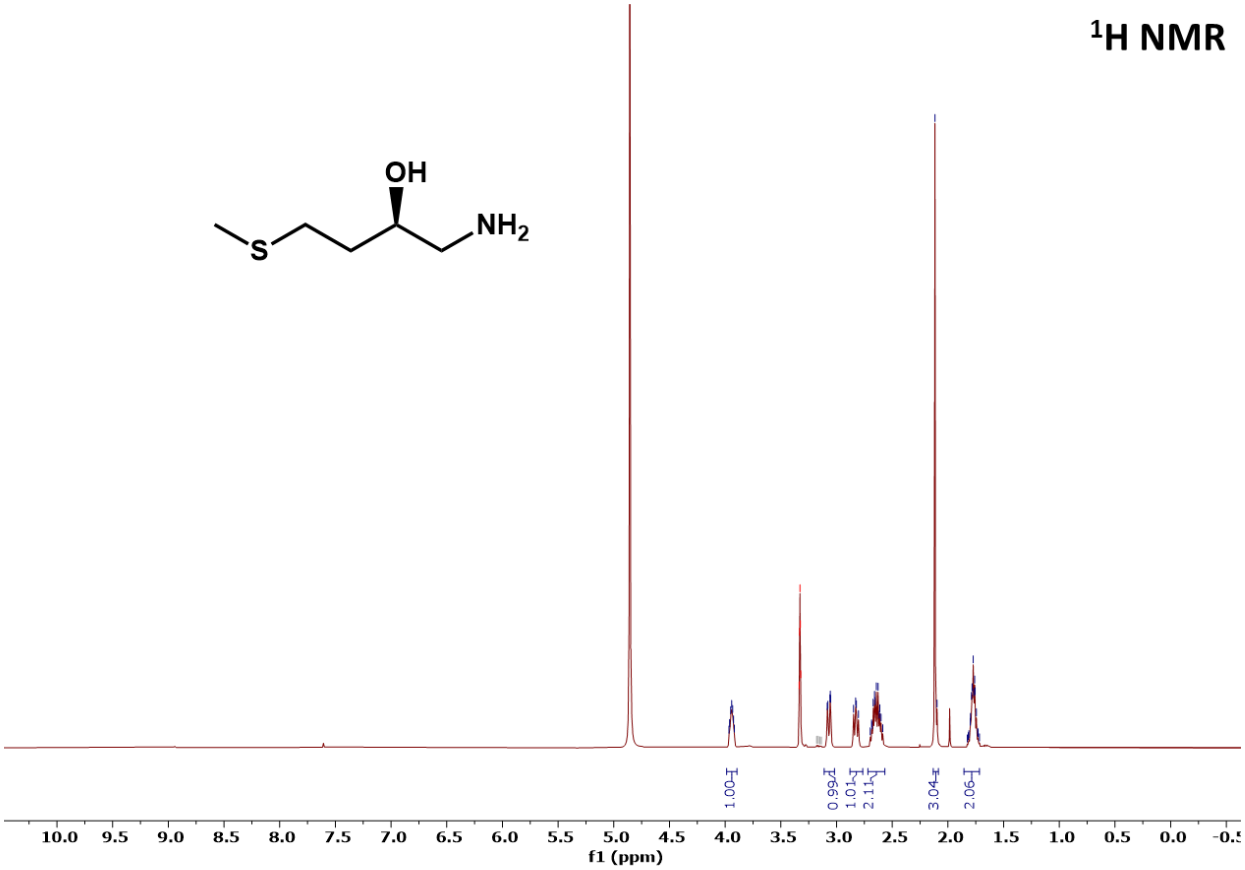

Figure S31. **^{1}H NMR spectrum of (*R*)-1-amino-4-(methylthio)butan-2-ol (14c).**

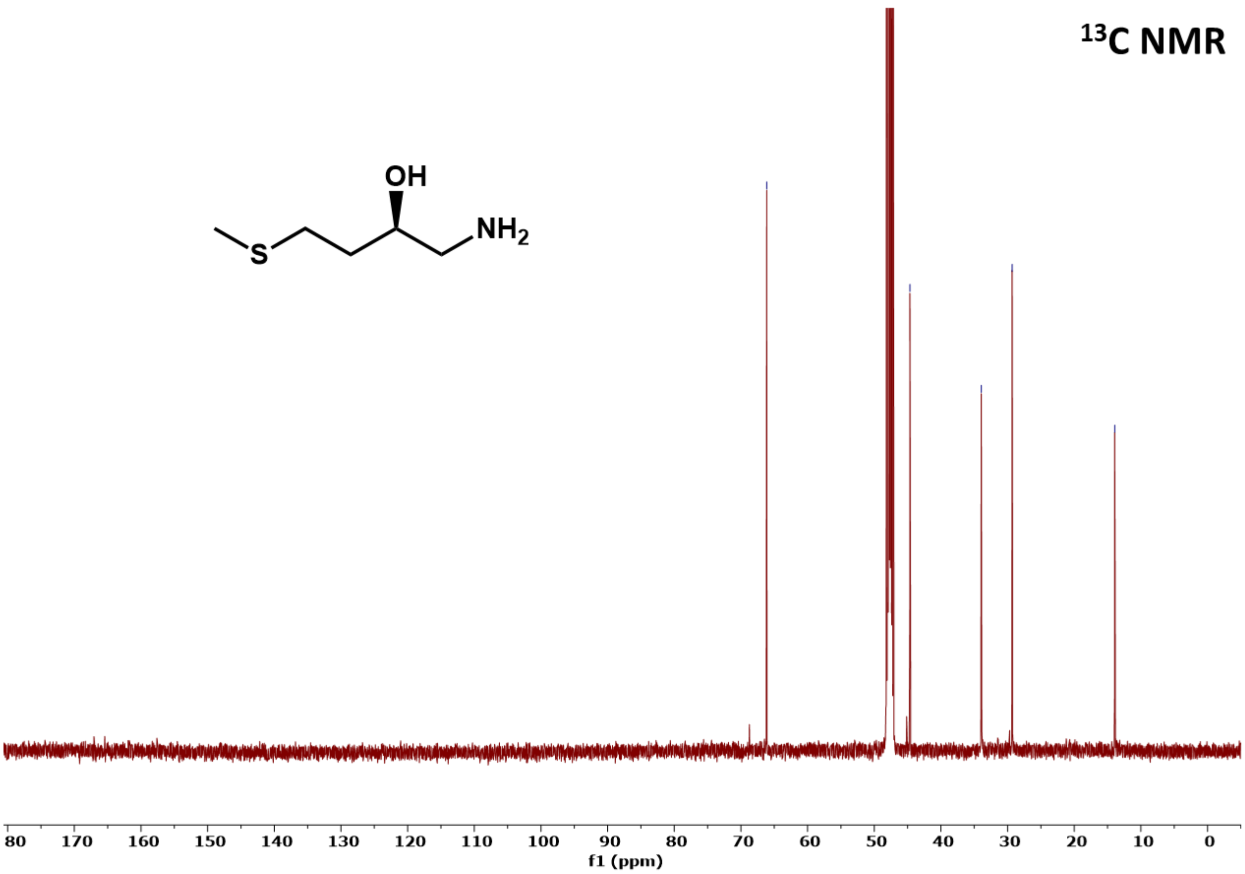

Figure S32. ^{13}**C NMR spectrum of (*R*)-1-amino-4-(methylthio)butan-2-ol (14c).**

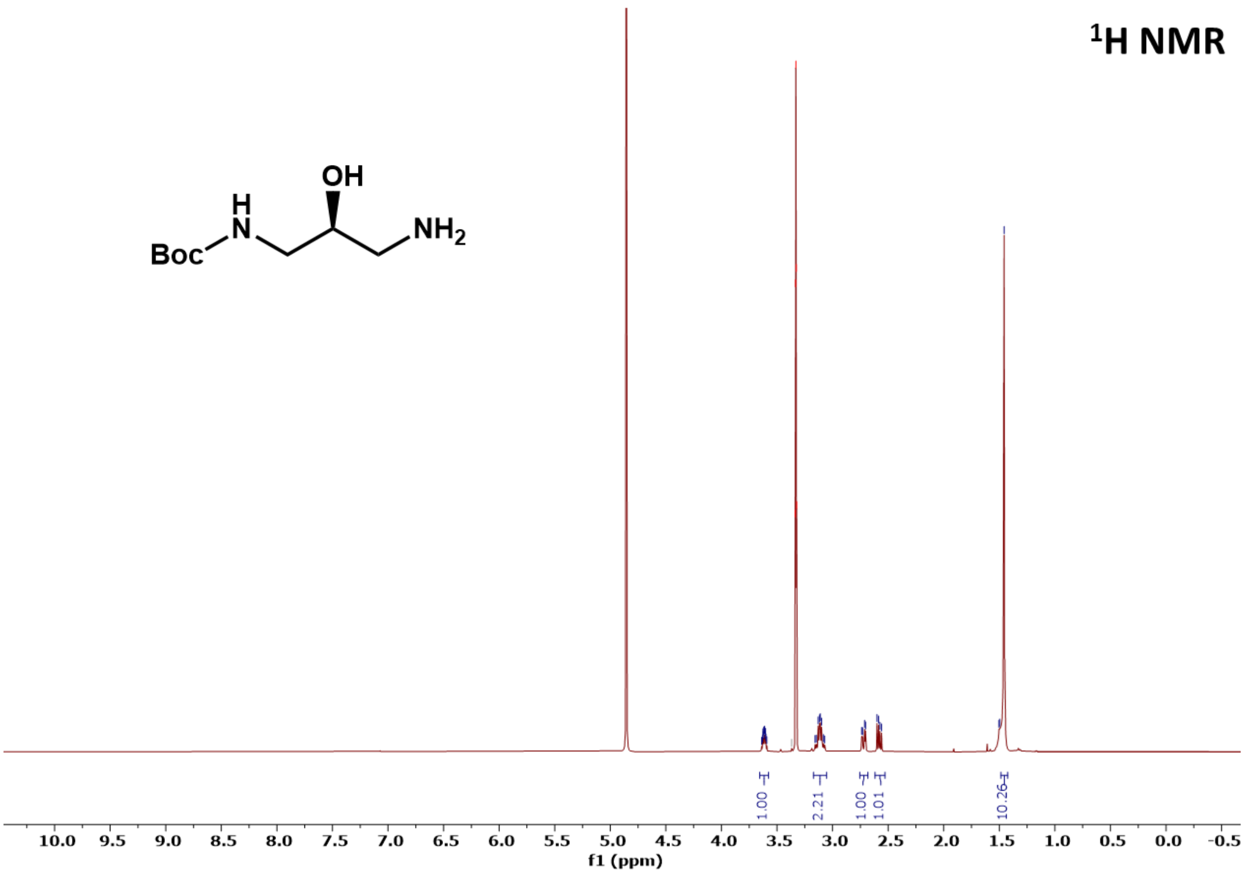

Figure S33. ^{1}H NMR spectrum of tert-butyl (*S*)-(3-amino-2-hydroxypropyl)carbamate (15c).

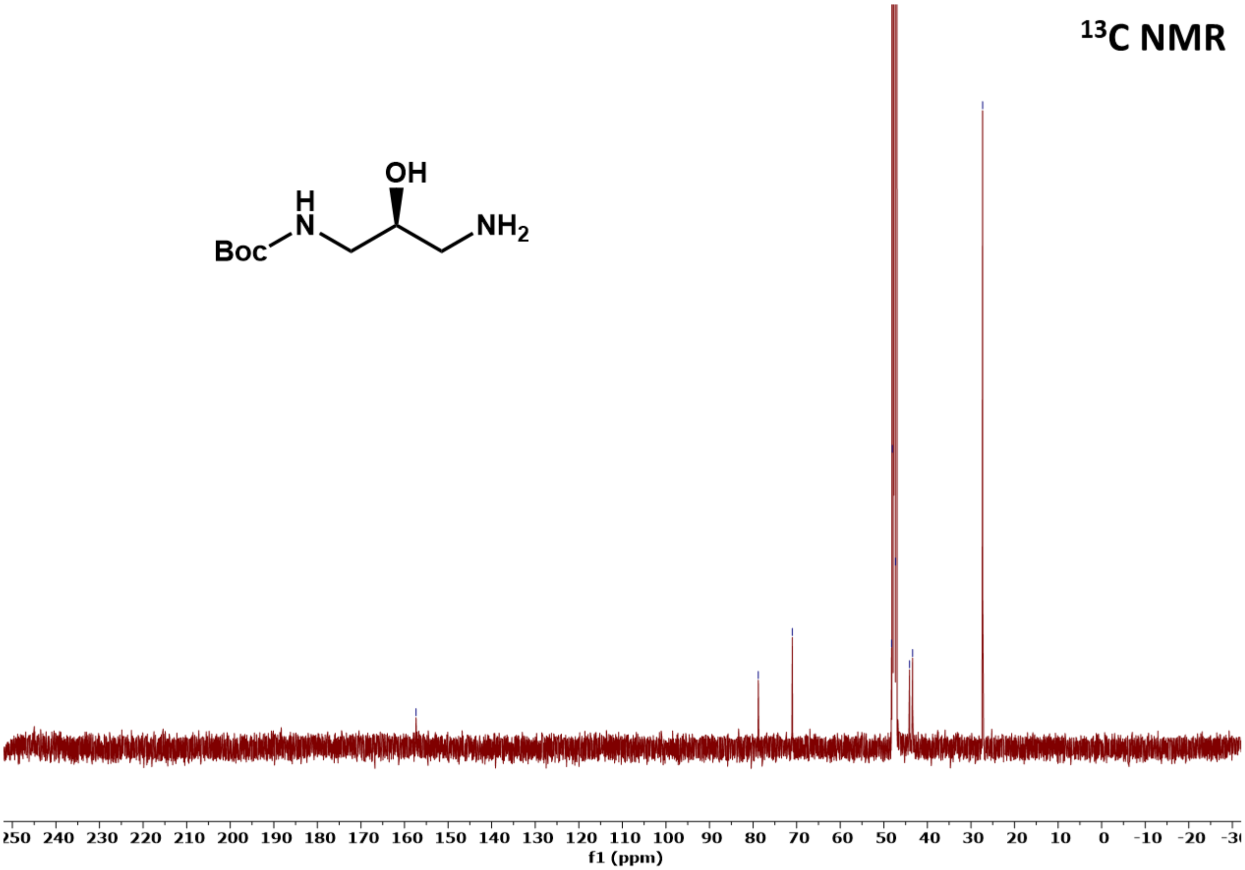

Figure S34. ^{13}**C NMR spectrum of tert-butyl (*S*)-(3-amino-2-hydroxypropyl)carbamate (15c).**

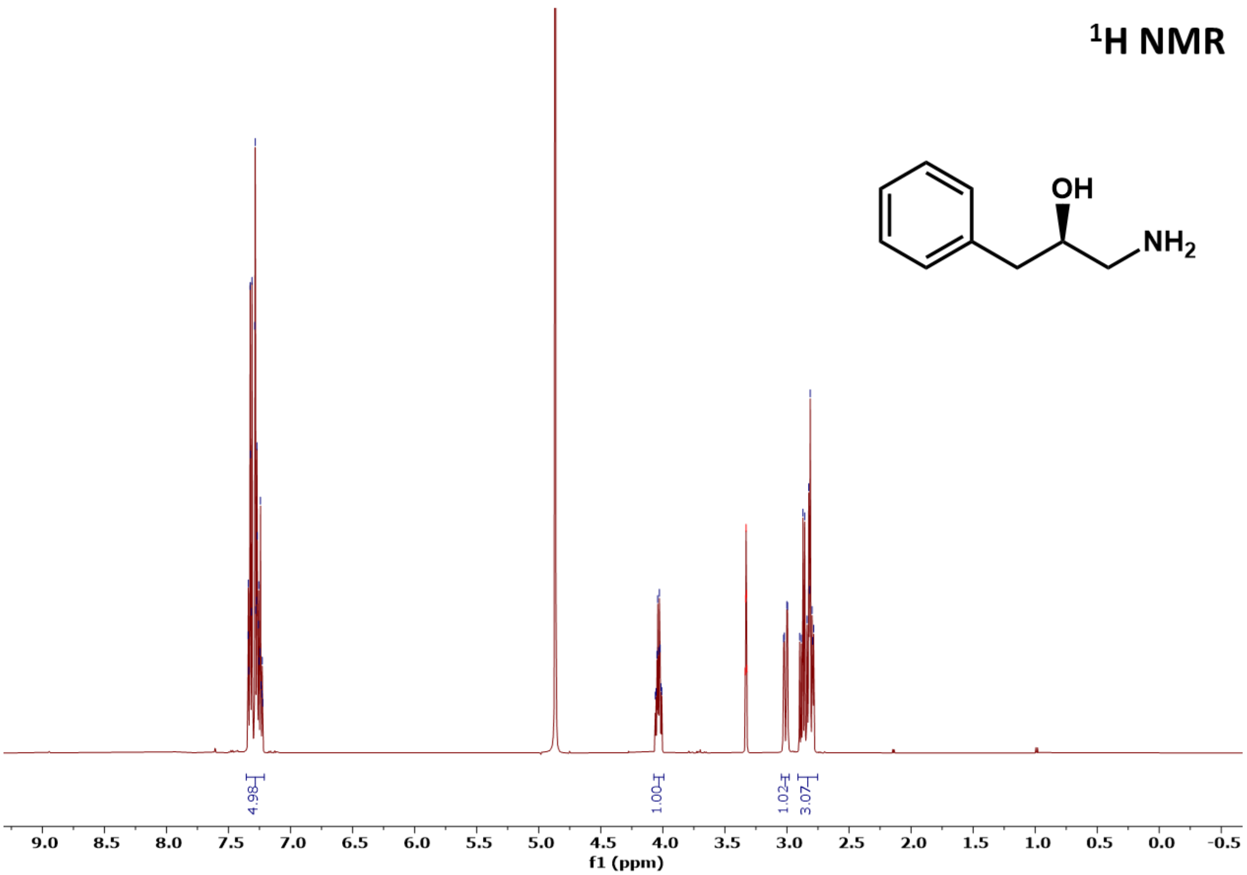

Figure S35. ^{1}H NMR spectrum of (*R*)-1-amino-3-phenylpropan-2-ol (3c).

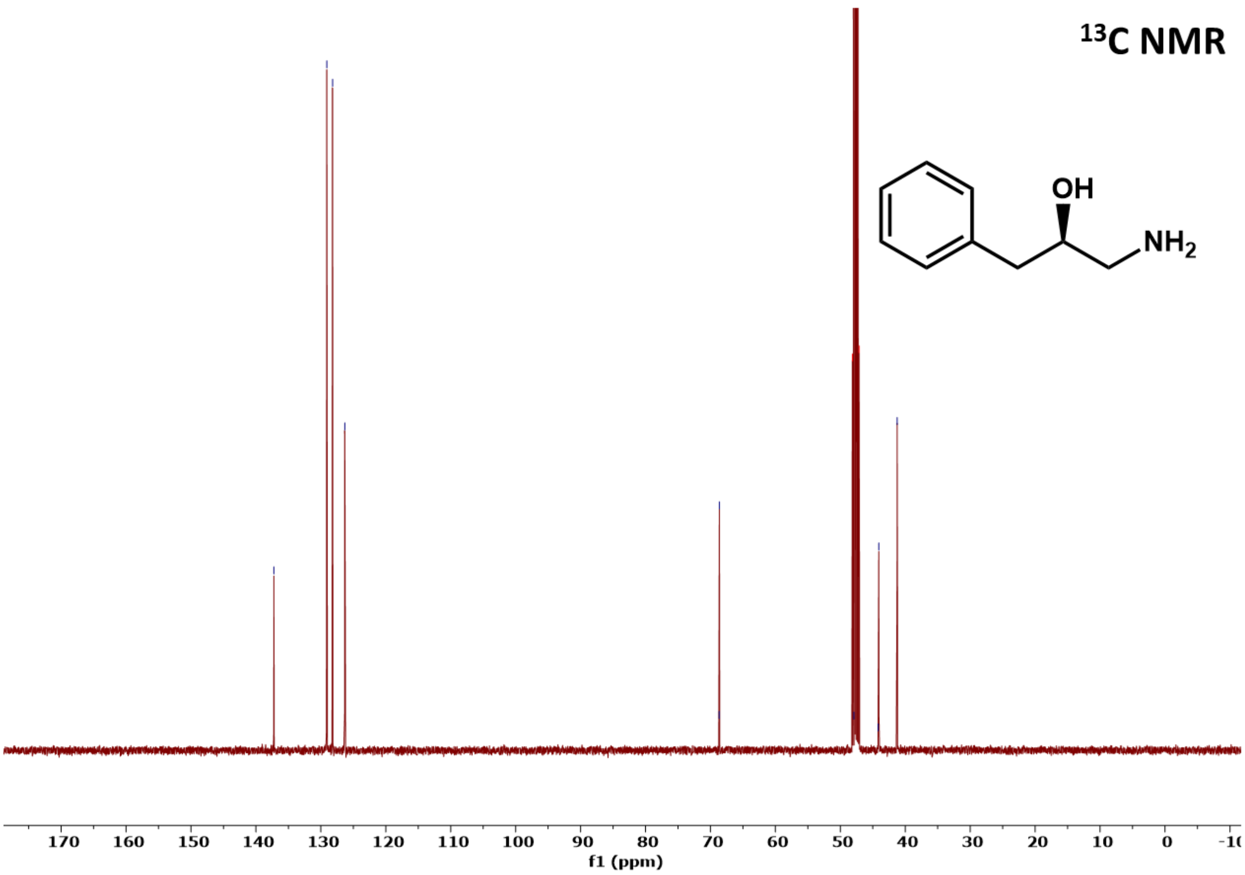

Figure S36. **^{13}C NMR spectrum of (*R*)-1-amino-3-phenylpropan-2-ol (3c).**

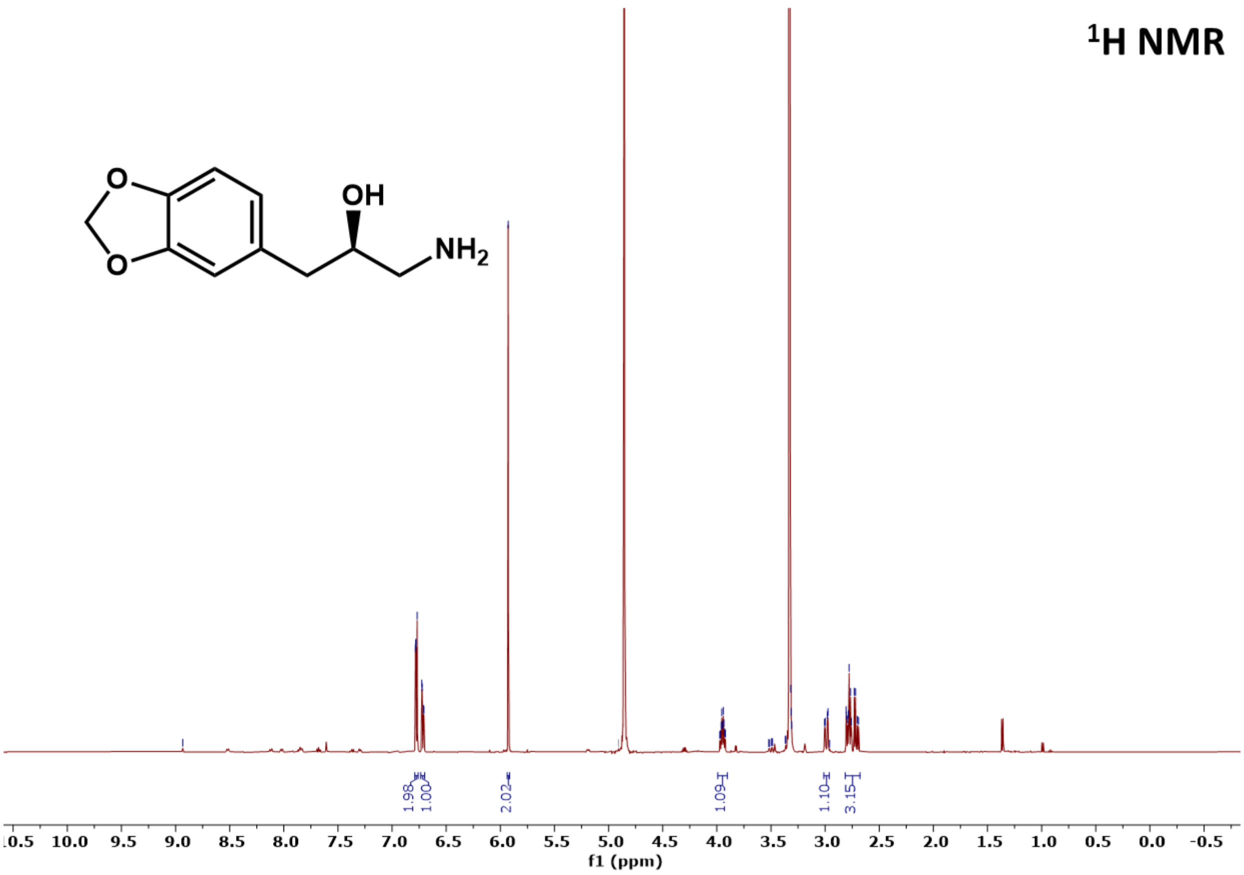

Figure S37. ^{1}**H NMR spectrum of (*R*)-1-amino-3-(benzo[d][1,3]dioxol-5-yl)propan-2-ol (20c).**

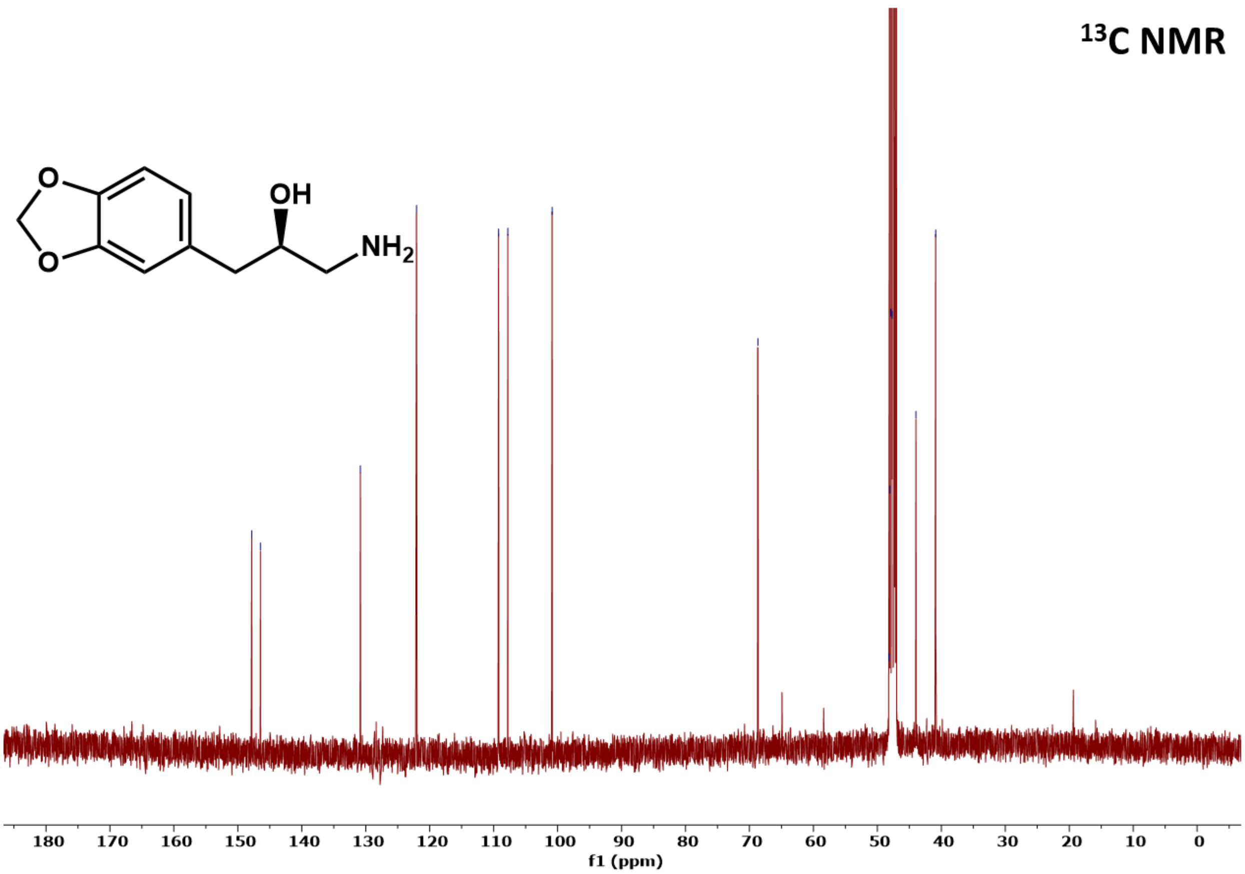

Figure S38. ^{13}**C NMR spectrum of (*R*)-1-amino-3-(benzo[d][1,3]dioxol-5-yl)propan-2-ol (20c).**

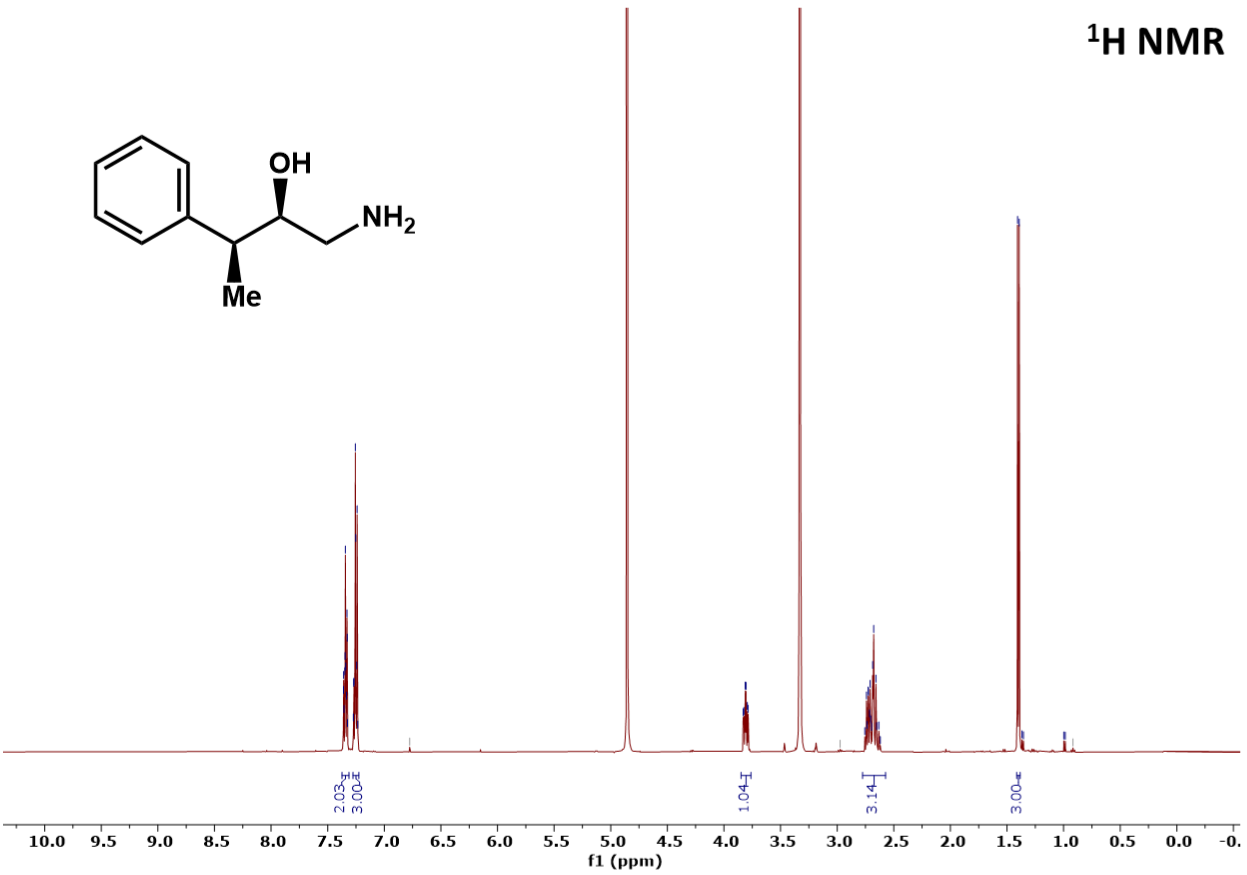

Figure S39. ^{1}**H NMR spectrum of (2*R*)-1-amino-3-phenylbutan-2-ol (21c).**

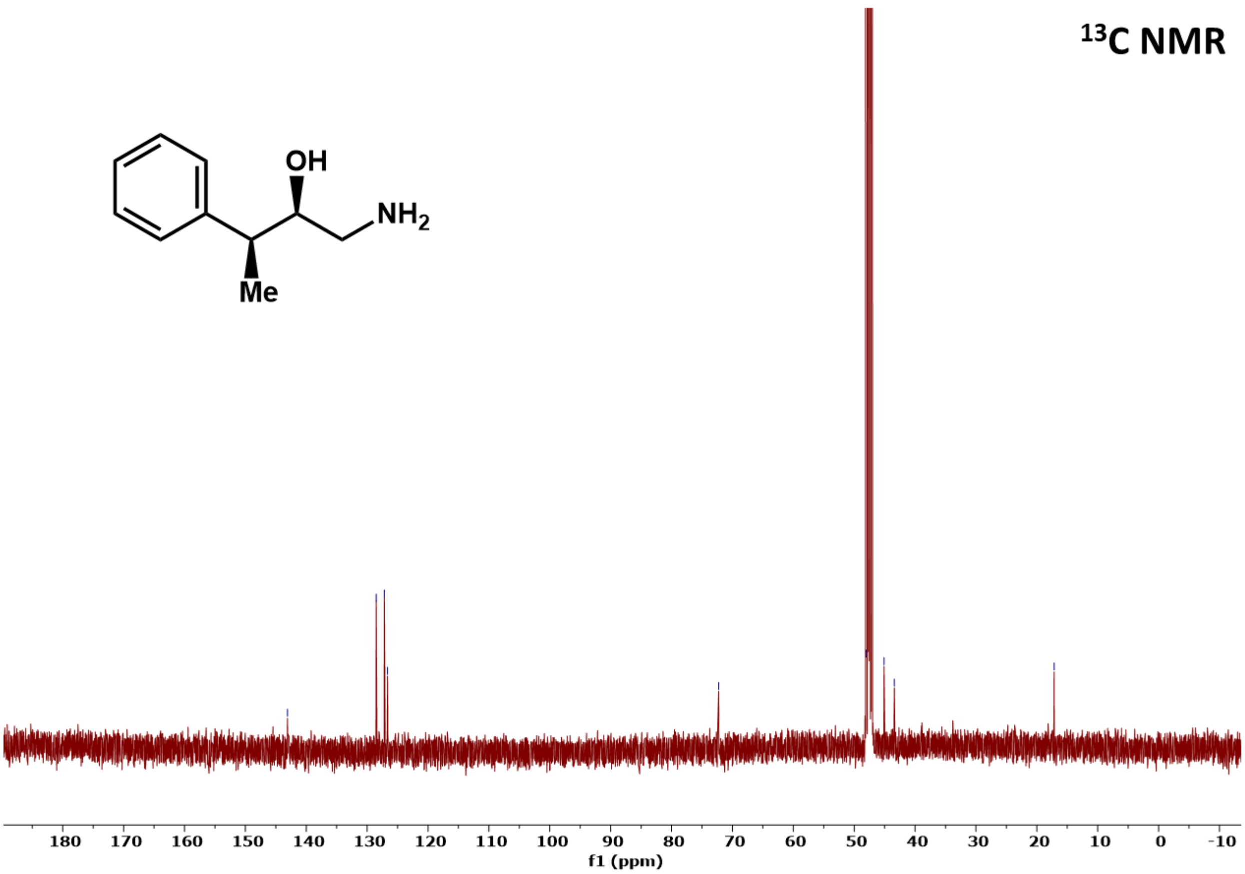

Figure S40. **^{13}C NMR spectrum of (2*R*)-1-amino-3-phenylbutan-2-ol (21c).**

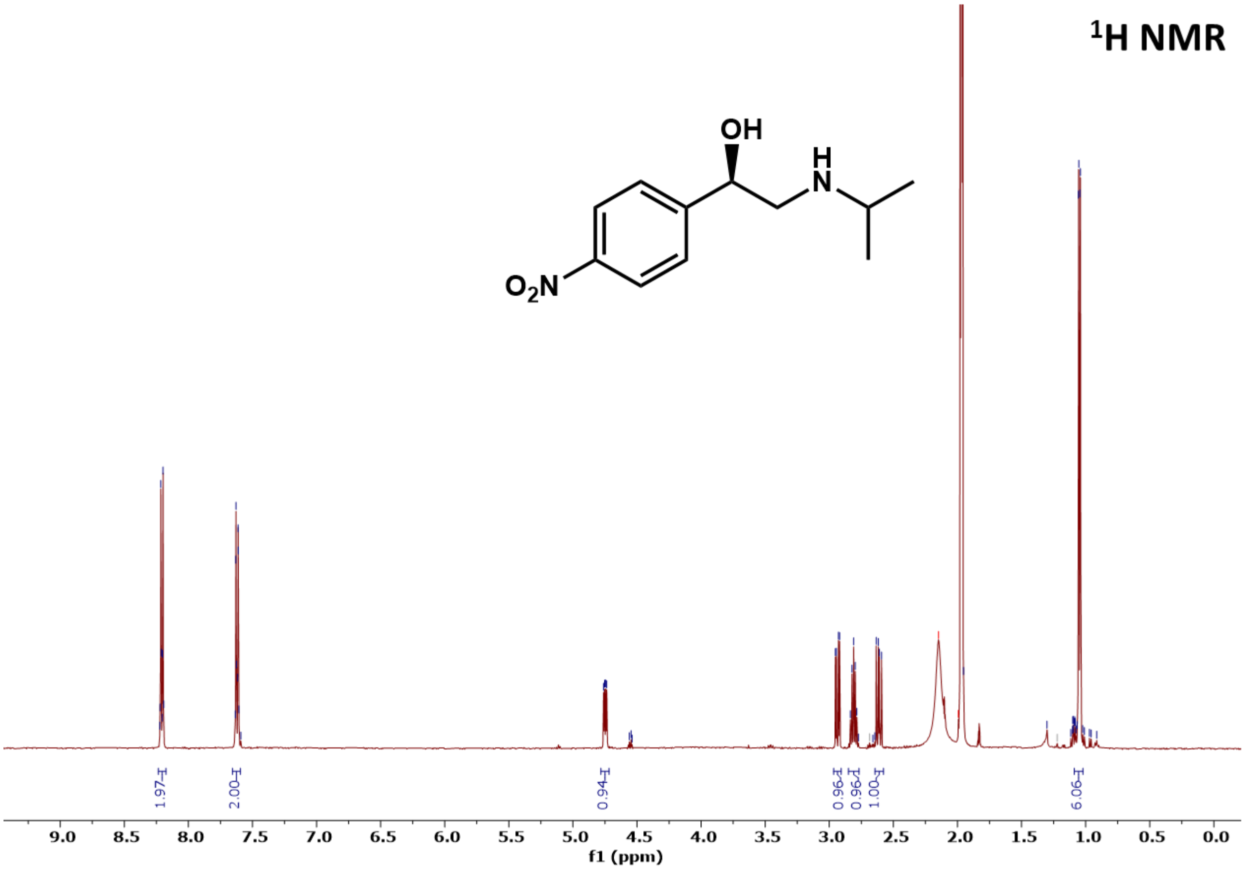

Figure S41. ^{1}H NMR spectrum of (*R*)-2-(isopropylamino)-1-(4-nitrophenyl)ethan-1-ol (7d).

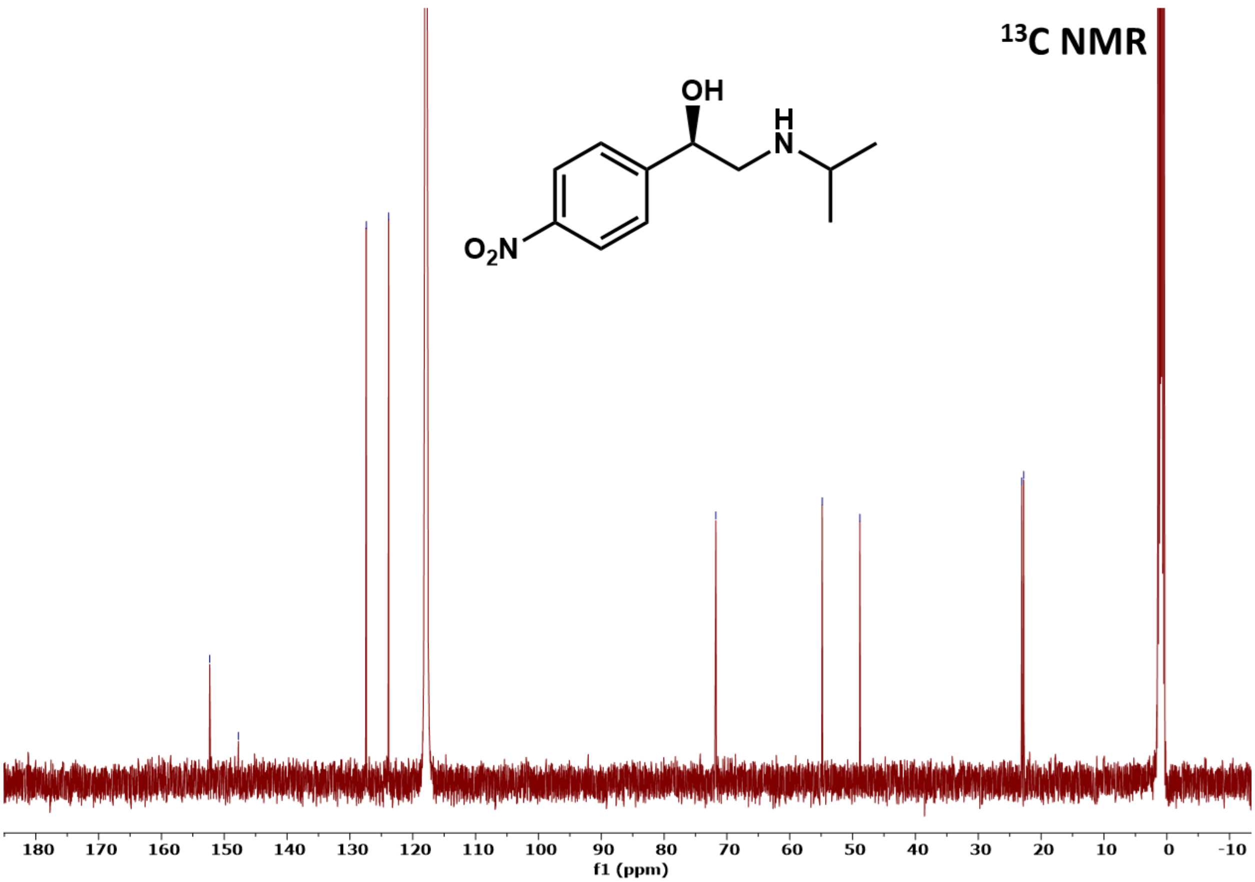

Figure S42. ^{13}**C NMR spectrum of (*R*)-2-(isopropylamino)-1-(4-nitrophenyl)ethan-1-ol (7d).**

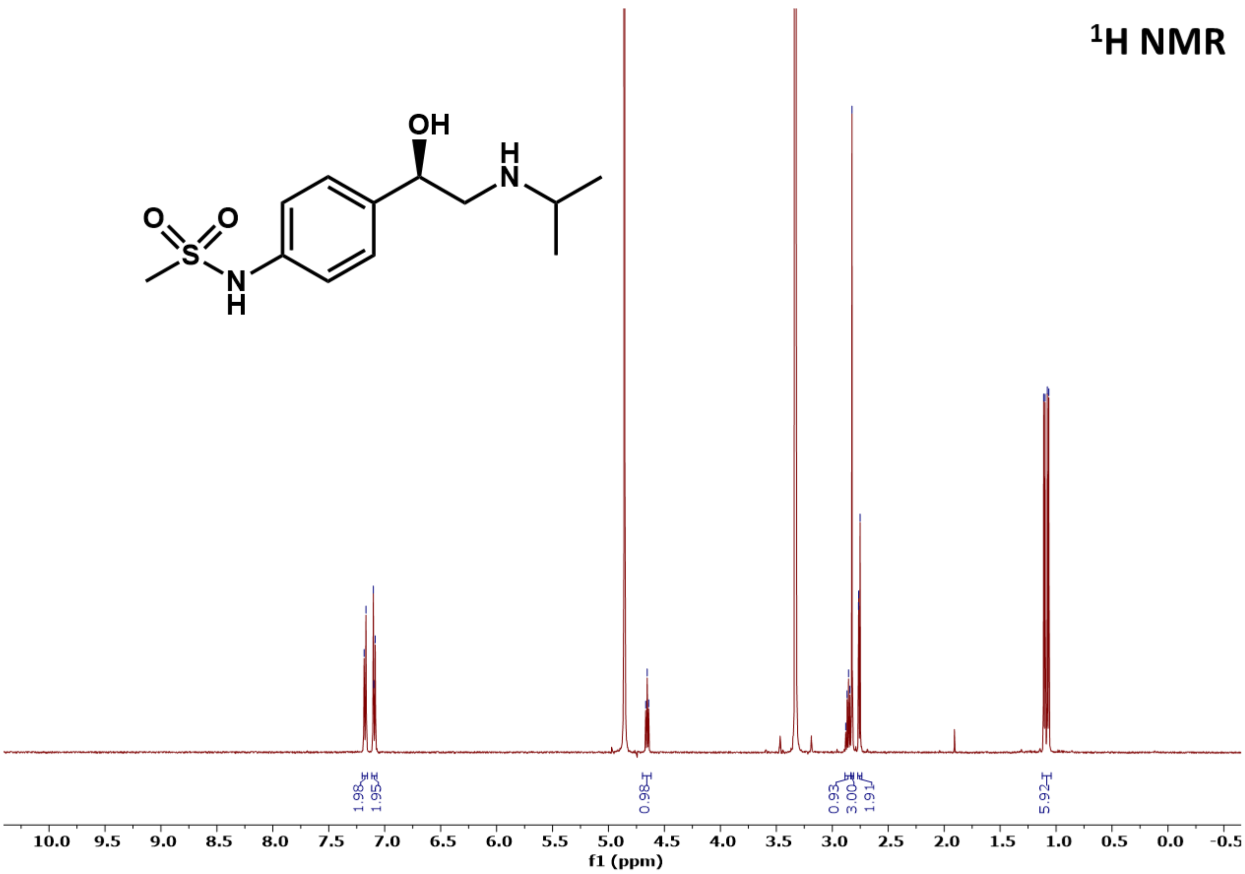

Figure S43. ^{1}**H NMR spectrum of (*R*)-N-(4-(1-hydroxy-2-(isopropylamino)ethyl)phenyl)methanesulfonamide (8d).**

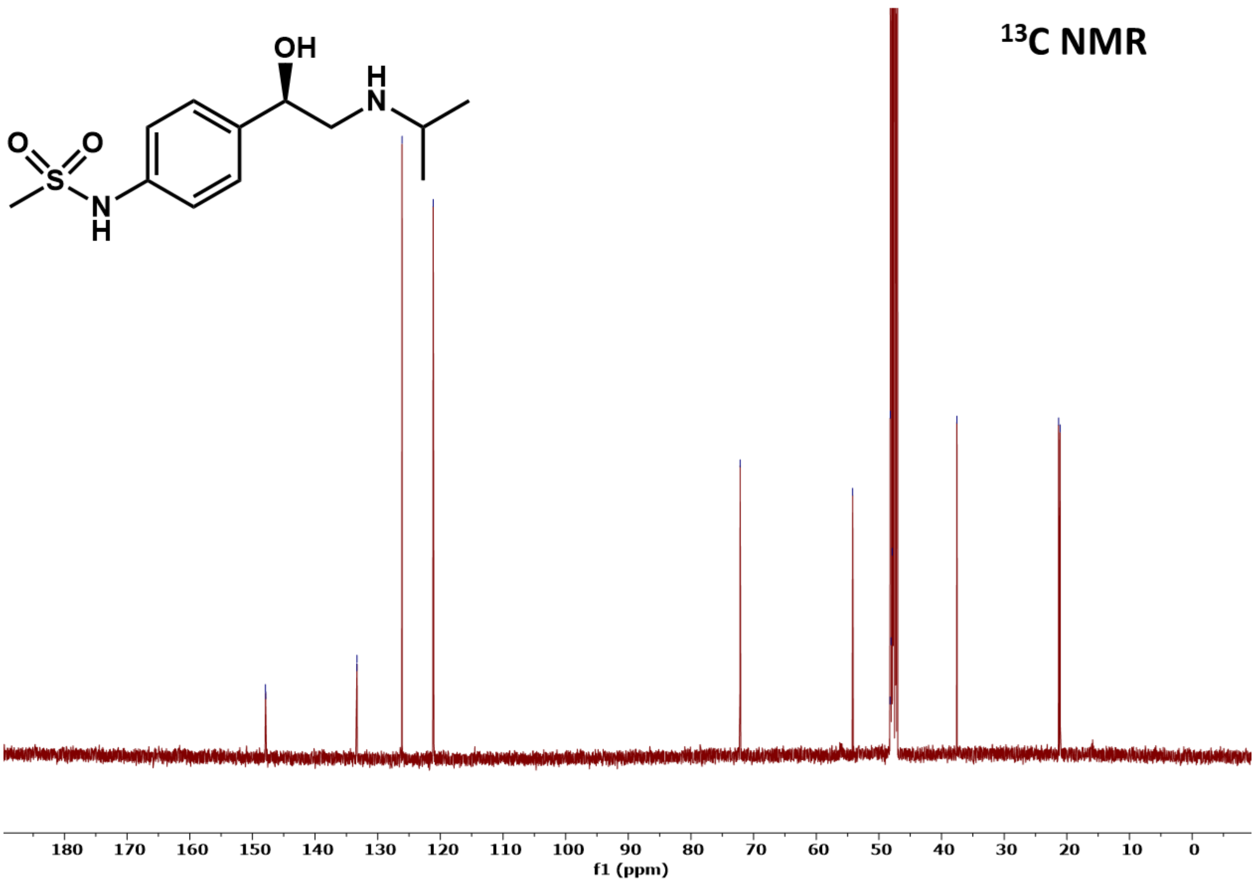

Figure S44. ^{13}C NMR spectrum of (*R*)-N-(4-(1-hydroxy-2-(isopropylamino)ethyl)phenyl)methanesulfonamide (8d).

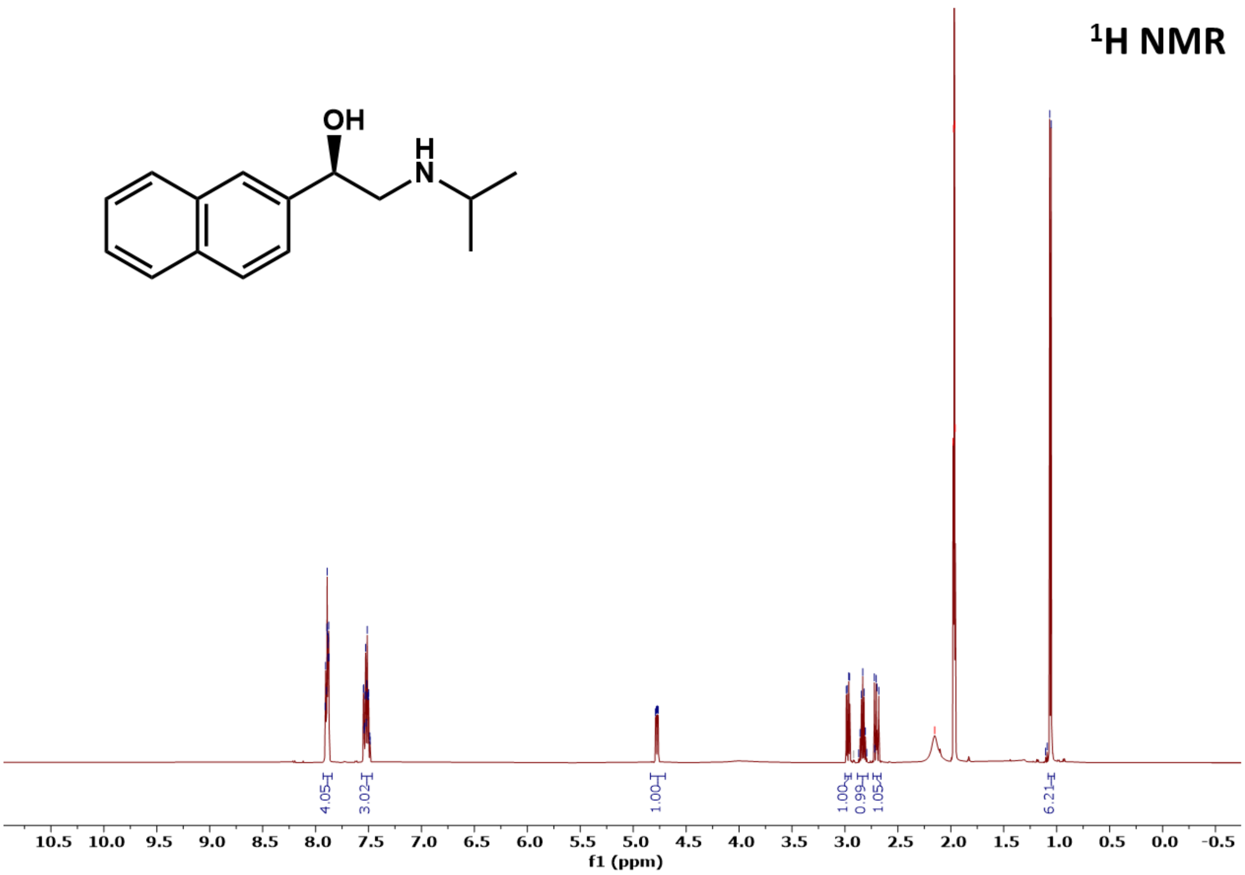

Figure S45. ^{1}H NMR spectrum of (*R*)-2-(isopropylamino)-1-(naphthalen-2-yl)ethan-1-ol (11d).

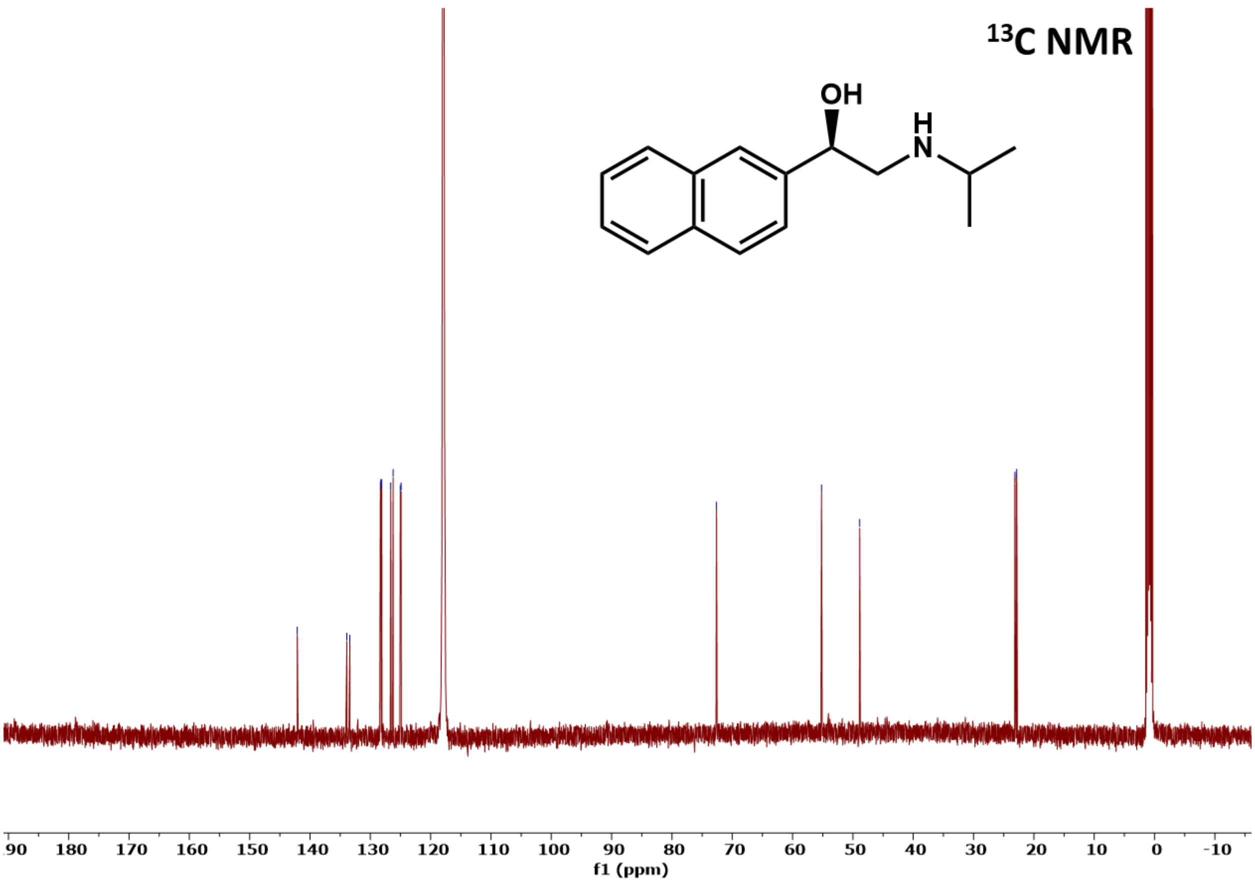

Figure S46. ^{13}**C NMR spectrum of (*R*)-2-(isopropylamino)-1-(naphthalen-2-yl)ethan-1-ol (11d).**

Supporting References

[1] T. J. Doyon, P. Kumar, S. Thein, M. Kim, A. Stitgen, A. M. Grieger, C. Madigan, P. H. Willoughby, A. R. Buller, *ChemBioChem*. **2022**, *23*, e202100577.

[2] A. Meza, M. E. Campbell, A. Zmich, S. A. Thein, A. M. Grieger, M. J. McGill, P. H. Willoughby, A. R. Buller, *ACS Catal*. **2022**, *12*, 10700-10710.

[3] J. R. Termaat, E. Pienaar, S. E. Whitney, T. G. Mamedov, A. Subramanian, *Microbiol Methods*. **2009**, *79*, 295-300.

[4] S. Kille, C. G. Acevedo-Rocha, L. P. Parra, Z. G. Zhang, D. J. Opperman, M. T. Reetz, J. P. Acevedo, *ACS Synth. Biol*. **2013**, *2*, 83-92.

[5] H. Brückner, C. Keller-Hoehl, *Chromatographia*. **1990**, *30*, 621-629.

[6] M. Tajbakhsh, R. Hosseinzadeh, H. Alinezhad, S. Ghahari, A. Heydari, S. Khaksar, *Synthesis (Stuttg)*. **2011**, *3*, 490-469.
